# Supplementary material for: Bump-and-Hole Engineering Identifies Specific Substrates of Glycosyltransferases in Living Cells
Source: Mol Cell. 2020 Jun 4;78(5):824–834.e15. doi: 10.1016/j.molcel.2020.03.030 (PMC7276986; doi:10.1016/j.molcel.2020.03.030)

Molecular Cell, Volume *78*

Supplemental Information

Bump-and-Hole Engineering

Identifies Specific Substrates

of Glycosyltransferases in Living Cells

Benjamin Schumann, Stacy Alyse Malaker, Simon Peter Wisnovsky, Marjoke Froukje Debets, Anthony John Agbay, Daniel Fernandez, Lauren Jan Sarbo Wagner, Liang Lin, Zhen Li, Junwon Choi, Douglas Michael Fox, Jessie Peh, Melissa Anne Gray, Kayvon Pedram, Jennifer Jean Kohler, Milan Mrksich, and Carolyn Ruth Bertozzi

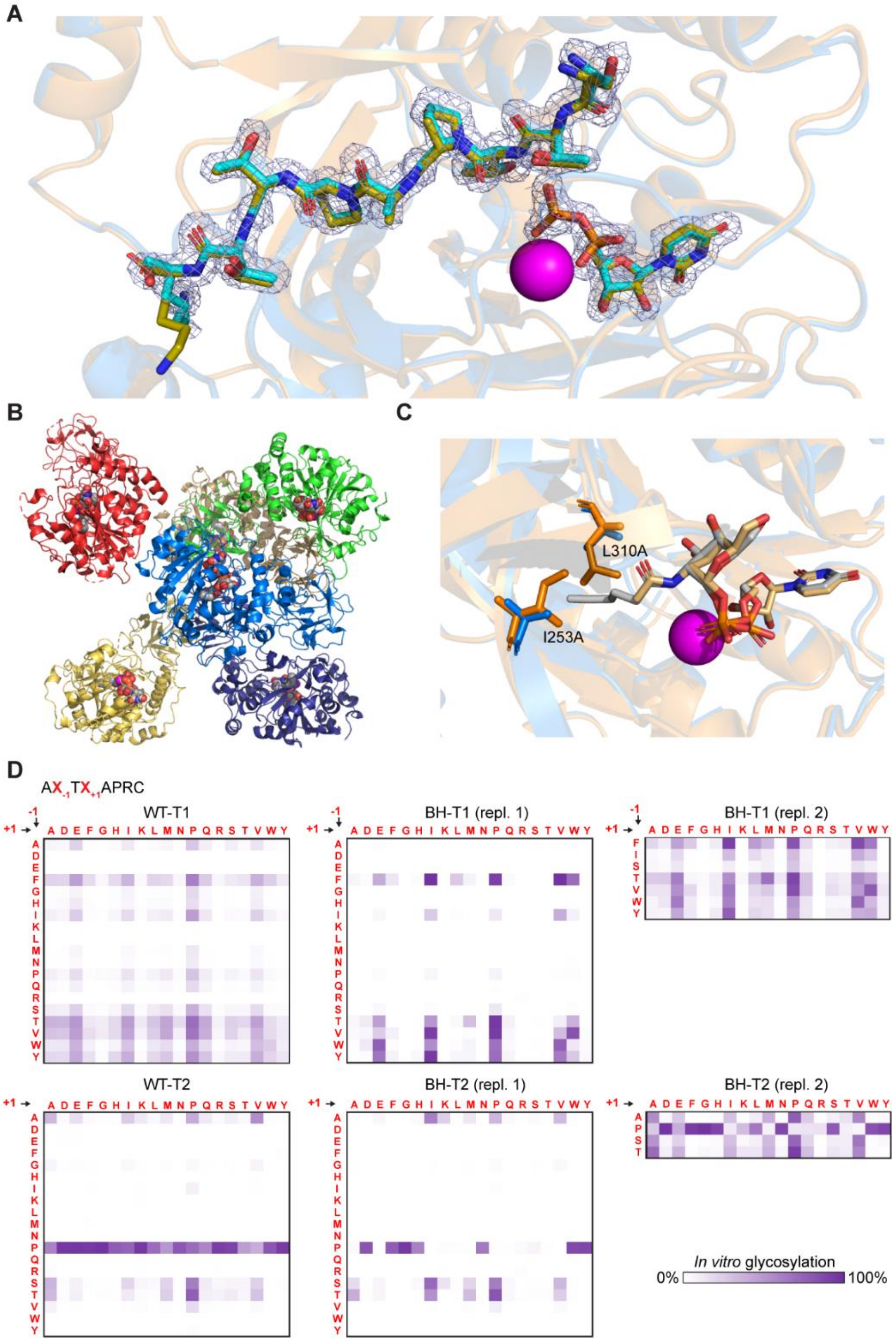

Figure S1. Validation of BH-GalNAc-Ts, Related to Figure 2 and Table 1.

(A) Overlay of ligands bound in BH-T2/EA2/UDP/Mn2+ (ligands cyan) and a published crystal structure of WT-T2/EA2/UDP/Mn2+ (ligands olive, PDB 2FFU), with electron density (map rendered at 1 σ and carved at 1.6 Å) taken from the BH-T2 co-crystal structure. Mn2+ ions are colored in magenta and overlay completely.

(B) Crystal structure of BH-T2/1/Mn2+ in the hexameric unit cell. UDP-GalNAc analog **1** is rendered in sphere representation.

(C) Superposition of the active sites of WT-T2 (PDB 4D0T, orange) with UDP-GalNAc (light brown) and BH-T2 (blue) with UDP-GalNAc analog **1** (grey). Gatekeeper residues are rendered in stick representation. Mn^{2+} ions are magenta and overlay completely.

(D) Substrate specificities of BH-T1 and BH-T2 and comparison with WT enzymes as determined in an *in vitro* glycosylation assay with detection by SAMDI-MS. WT data corresponds with reported substrate specificities, and two replicates are shown for BH enzymes with the full library or a focused sub-library.

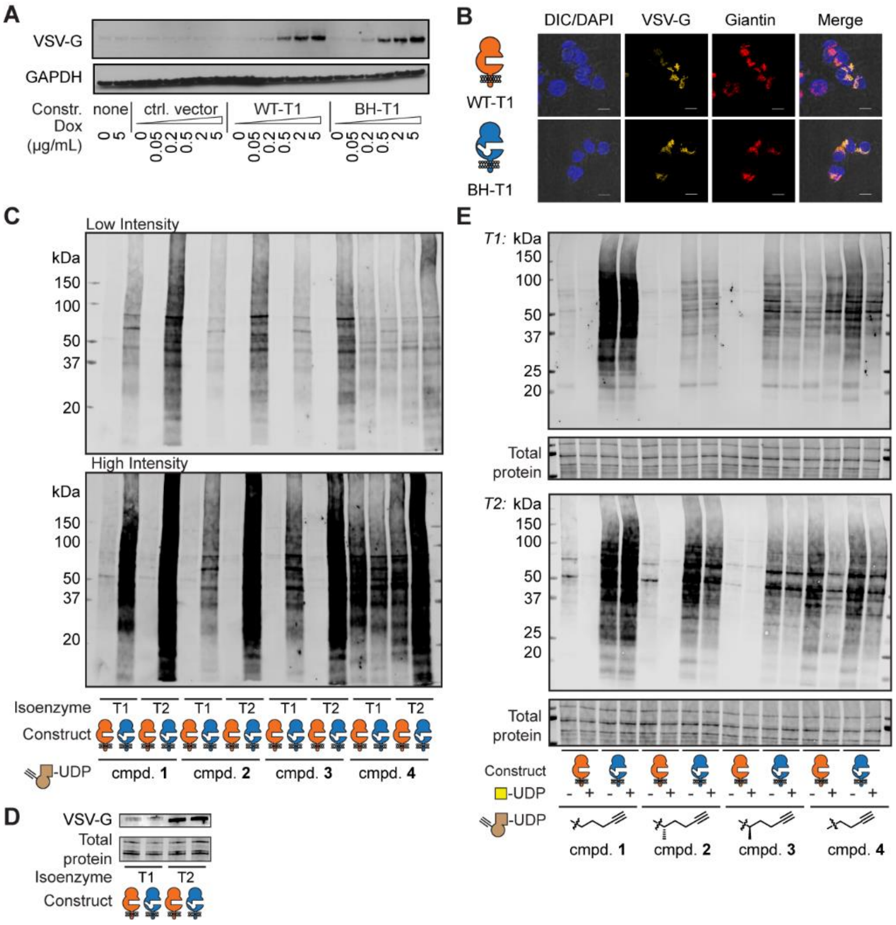

Figure S2. Localization of BH-GalNAc-T1 to the Golgi compartment and glycosylation of proteins, Related to Figure 3.

(A) WT-T1 and BH-T1 are expressed by stably transfected HepG2 cells in a Dox-inducible fashion.

(B) Fluorescence microscopy of HepG2 cells stably transfected with T1 constructs, induced with 2 µg/mL Dox and subsequently stained. Inset: magnification of a single cell. Scale bar, 10 µm.

(C) Streptavidin blot from Figure 3C depicted in two different intensities.

(D) Expression of GalNAc-Ts in lysates used in (C).

(E) *In vitro* glycosylation was repeated with a membrane fraction from untransfected HepG2 cells, using soluble GalNAc-Ts to perform glycosylations (Choi et al., 2019). Reactions were performed with or without a two-fold excess of UDP-GalNAc over UDP-sugars **1**, **2**, **3** and **4**.

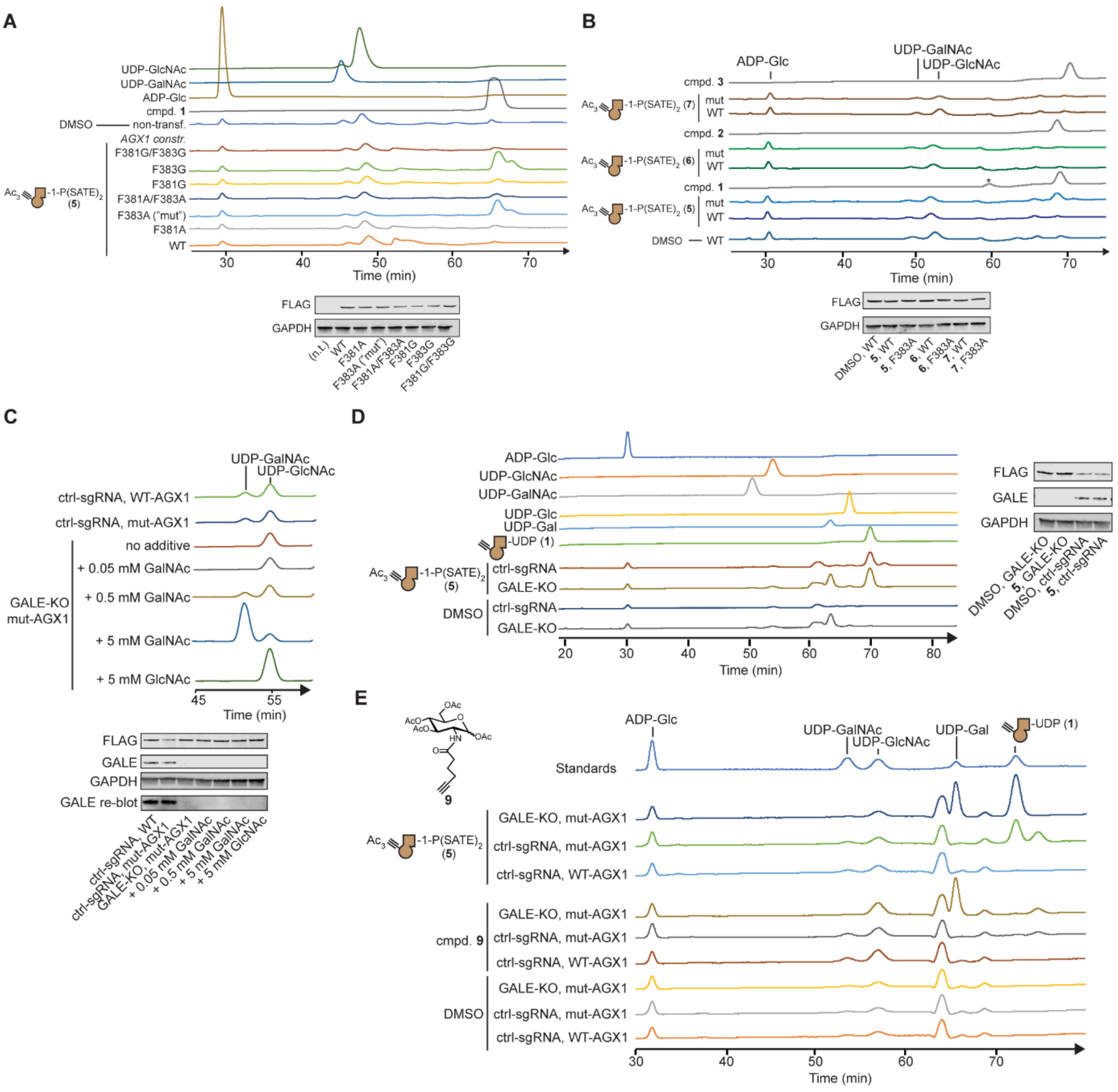

Figure S3. Biosynthesis of UDP-GalNAc analog 1 by mut-AGX1 and epimerization by GALE, Related to Figure 4.

(A) HPAEC-PAD traces of extracts from HEK293T cells transiently expressing AGX1 constructs, and fed with GalNAc-1-phosphate analog **5** or DMSO. All traces are normalized to the retention time of ADP-Glc as an external standard. Data is of a single experiment.

(B) Full traces of the data displayed in Fig. 4B. Asterisk indicates an artefact from solvent filling.

(C) HPAEC-PAD traces of GALE-KO or control sgRNA-transduced K-562 cells stably transfected with the indicated FLAG-tagged AGX1 constructs and fed with different concentrations of GalNAc or GlcNAc. Expression of AGX1 (FLAG) and GALE were analyzed by Western blot. Samples were re-blotted with a higher concentration of GALE antibody to assure absence of GALE in KO cells. Data are from one experiment.

(D) Cells were fed with DMSO or compound **5**, and UDP-sugar production was measured by HPAEC-PAD. GALE-KO contain elevated levels of UDP-Gal as cells are supplemented with galactose to maintain viability and UDP-Gal cannot be epimerized to UDP-Glc. Expression levels of AGX1 (FLAG) and GALE are analyzed by Western blot. Data are of one representative out of two independent experiments.

(E) GALE-KO or control sgRNA-transduced

(F) K-562 cells stably transfected with the indicated AGX1 constructs were fed with the indicated compounds, and UDP-sugar production was measured by HPAEC-PAD. Data are from one experiment.

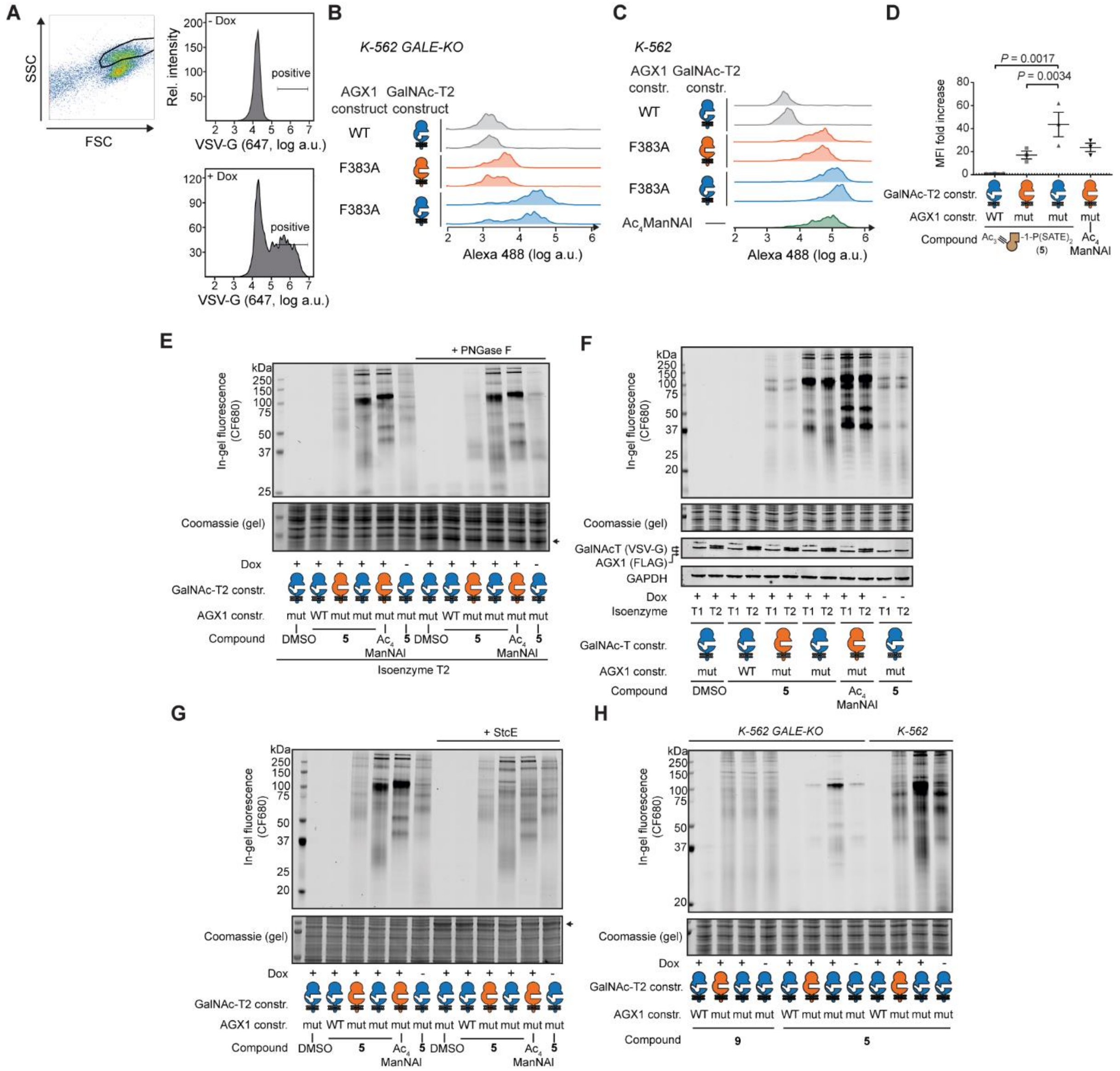

Figure S4. Bioorthogonal, GalNAc-T-selective cell surface labeling, Related to Figure 5.

- (A) Gating scheme for flow cytometry experiments, and in-gel fluorescence experiments.
- (B) Primary flow cytometry data of the experiment in Figure 5C. Two technical replicates are shown.
- (C) Primary flow cytometry data of K-562 cells after induction with 0.5 µg/mL Dox and gating on VSV-G-positive cells. Two technical replicates are shown for cells treated with compound **5**.
- (D) Statistical analysis of the experiment in (C). Data are represented as individual values from three independent experiments, mean ± SEM of MB488 median fluorescence intensity of VSV-G positive cells. Statistical analysis was performed by two-tailed ratio paired t-test.
- (E) Cells expressing AGX1 and GalNAc-T2 constructs were labeled as in Figure 5C and analyzed by in-gel fluorescence. Treatment of lysates with PNGase F significantly shifts certain background bands to lower molecular weight. Arrow indicates PNGase F band in Coomassie stain.
- (F) Full gel of the experiment depicted in Figure 5C. The sialic acid precursor Ac_4ManNAl was used as a positive control, and the effect of omitting Dox was investigated. In-gel fluorescence and Coomassie staining are from one gel and expression analyses are from one separate Western Blot.
- (G) Treatment of lysates prepared as in (A) with the glycoprotease StcE. Arrow indicates StcE band in Coomassie stain.
- (H) Dissecting GlcNAc vs. GalNAc labeling by using probes **5** and **9**. K-562 GALE-KO cells expressing AGX1 and GalNAc-T2 constructs were treated with Dox or left untreated, and fed with compounds **5** or **9**. Labeling was performed as in Figure 5C. K-562 cells prepared as in (A) were used to compare labeling patterns. Data are from one representative out of three independent experiments.

At least 500 gated cells were used for analysis per sample in (B) and (C).

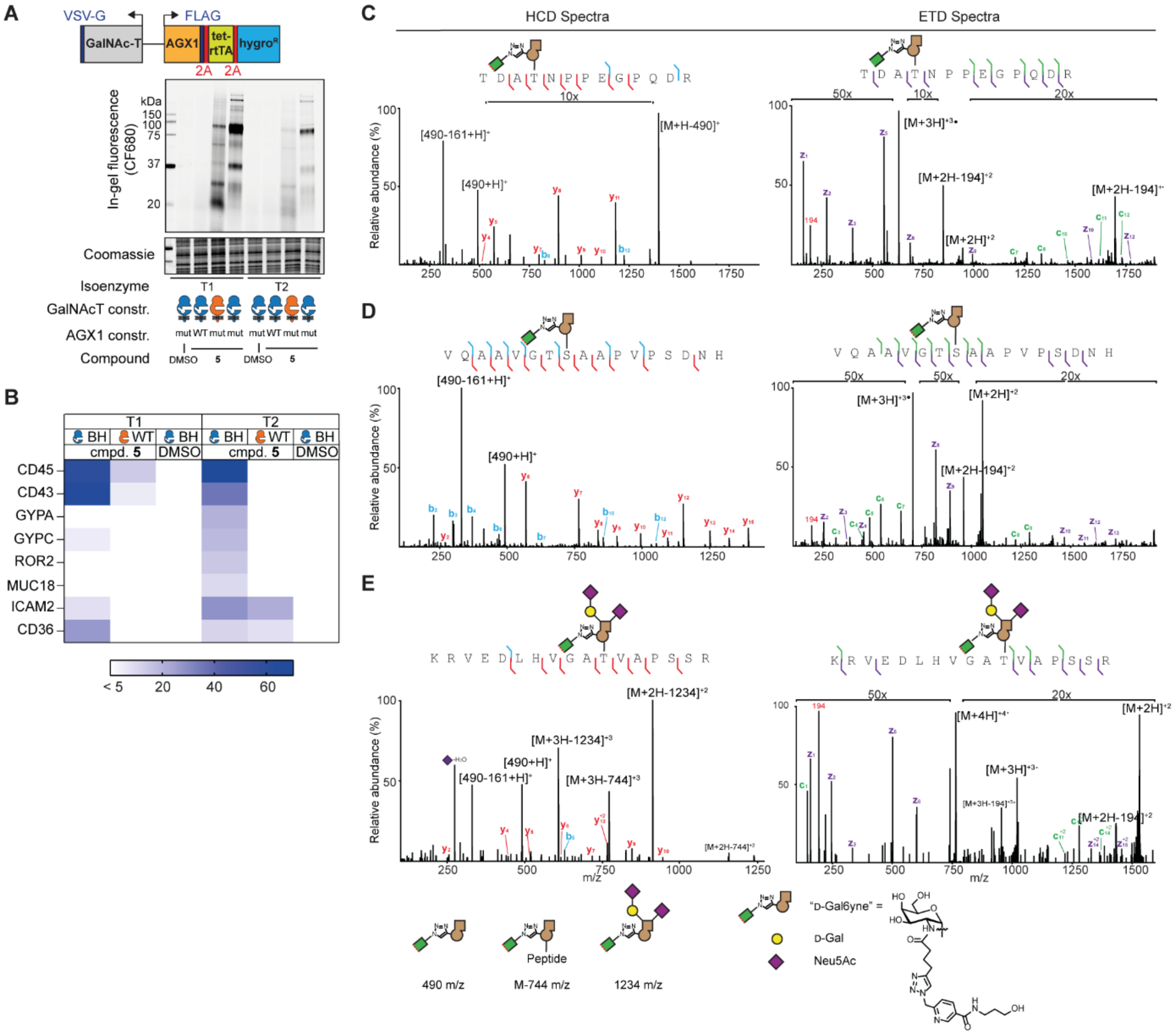

Figure S5. GalNAc-T bump-and-hole pairs selectively label O-glycosylated proteins, Related to Figure 5.

(A) In-gel fluorescence of cell surface proteins in K-562 cells stably expressing GalNAc-T and AGX1 constructs. Cells and samples were treated as in Figure 5C.

(B) Mass spectrometry proteomics. Heat map represents log prob of detected proteins after enrichment of cell surface proteins of K-562 cells expressing mut-AGX1 and indicated GalNAc-T constructs and fed with the indicated compounds.

(C-E) Exemplary mass spectra from glycopeptides after modification by BH-T2. HCD (mainly glycan fragmentation) and ETD (mainly peptide fragmentation) spectra are shown, and ions are annotated. y and b ions in HCD spectra were devoid of glycan.

(C) Glycosylation at Thr28 of STC2 as a newly-identified T2-specific modification (Luo et al., 2005).

(D) Confirmation of Ser308 as a T2-specific modification of ApoE.

(E) Extension of chemically tagged GalNAc by elaborating glycosyltransferases on T39 of SERPIN5A. Legend depicts tentative structural assignments of neutral loss fragments. Loss of 161 m/z in HCD and 194 m/z peak in ETD likely depicts fragment masses of the triazole-based linker.

Methods S1, Related to STAR Methods. Characterization of synthetic compounds.

Bis(*S*-acetyl-2-thioethyl) 3,4,6-tri-*O*-acetyl-2-deoxy-2-(5-hexynoyl)amido-α-D-galactopyranosyl phosphate (5)

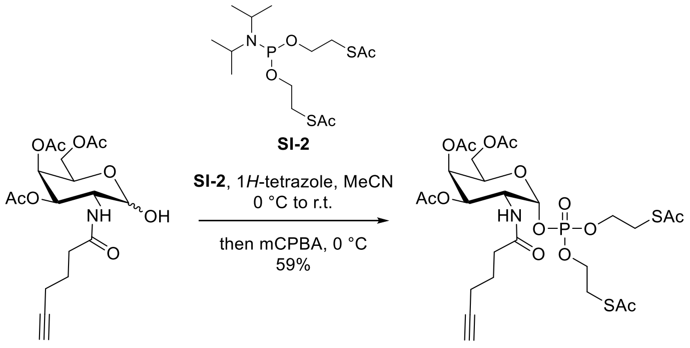

SI-1 **5**

^{1}H NMR (600 MHz, $CDCl_3$) δ 6.24 (d, *J* = 9.2 Hz, 1H), 5.75 (dd, *J* = 5.6, 3.3 Hz, 1H), 5.44 (dd, *J* = 3.2, 1.4 Hz, 1H), 5.18 (dd, *J* = 11.5, 3.2 Hz, 1H), 4.81 – 4.60 (m, 1H), 4.40 (td, *J* = 6.6, 1.4 Hz, 1H), 4.29 – 3.93 (m, 4H), 3.34 – 3.06 (m, 6H), 2.37 – 2.30 (m, 8H), 2.28 – 2.17 (m, 2H), 2.15 (s, 3H), 2.03 (s, 3H), 1.99 (s, 3H), 1.95 (t, *J* = 2.6 Hz, 1H), 1.88 – 1.76 (m, 2H); ^{13}C NMR (150 MHz, $CDCl_3$) δ 195.3, 194.9, 172.8, 170.7, 170.4, 170.2, 97.3, 97.3, 83.4, 69.5, 69.3, 68.8, 67.4, 67.3, 66.9, 66.8, 66.6, 66.5, 66.5, 66.5, 61.5, 47.6, 47.6, 47.5, 47.4, 34.8, 34.7, 30.8, 30.7, 30.6, 29.2, 29.2, 29.2, 24.1, 24.0, 20.8, 20.7, 20.6, 17.8, 17.7; HRMS (ESI) calcd. for $C_{26}H_{38}NO_{14}PS_2Na$ ($M+Na^+$) 706.1369 found 706.1349 *m/z*.

Bis(*S*-acetyl-2-thioethyl) 3,4,6-tri-*O*-acetyl-2-deoxy-2-(2-(*S*)-methyl-5-hexynoyl)amido-α-D-galactopyranosyl phosphate (6)

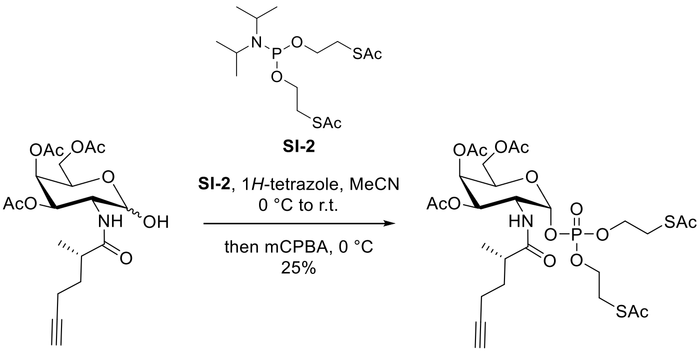

SI-3 **6**

^{1}H NMR (400 MHz, acetone-D_6) δ 7.36 (d, *J* = 8.3 Hz, 1H), 5.78 (dd, *J* = 6.0, 3.4 Hz, 1H), 5.50 (dd, *J* = 3.2, 1.4 Hz, 1H), 5.17 (dd, *J* = 11.8, 3.2 Hz, 1H), 4.71 – 4.44 (m, 2H), 4.31 – 4.03 (m, 6H), 3.22 (t, *J* = 6.5 Hz, 4H), 2.62 – 2.44 (m, 1H), 2.44 – 2.28 (m, 7H), 2.19 – 2.11 (m, 5H), 2.01 (s, 3H), 1.94 (s, 3H), 1.90 – 1.75 (m, 1H), 1.61 – 1.49 (m, 1H), 1.12 (d, *J* = 6.9, 1.6 Hz, 3H); ^{13}C NMR (100 MHz, acetone-D_6) δ 195.2, 177.5, 176.5, 170.7, 170.6, 170.4, 97.6, 84.3, 70.2, 69.4, 67.9, 67.7, 66.9, 62.4, 48.1, 40.3, 33.8, 20.7, 20.6, 18.0, 16.8; HRMS (ESI) calcd. for $C_{27}H_{40}NO_{14}PS_2Na$ ($M+Na^+$) 720.1526 found 720.1523 *m/z*.

Bis(*S*-acetyl-2-thioethyl) 3,4,6-tri-*O*-acetyl-2-deoxy-2-(2-(*R*)-methyl-5-hexynoyl)amido-α-D-galactopyranosyl phosphate (7)

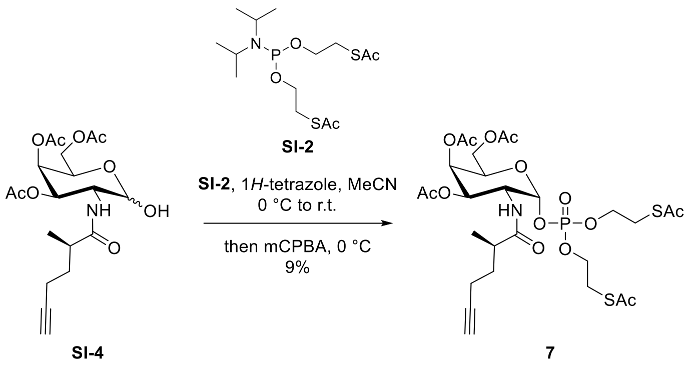

^{1}H NMR (600 MHz, acetone-D_6) δ 7.35 (d, *J* = 8.2 Hz, 1H), 5.81 (m, 1H), 5.50 (s, 1H), 5.18 (m, 1H), 4.59 – 4.43 (m, 2H), 4.33 – 4.01 (m, 6H), 3.31 – 3.07 (m, 4H), 2.55 – 2.45 (m, 1H), 2.40 – 2.30 (m, 7H), 2.25 – 2.12 (m, 5H), 2.01 (s, 3H), 1.97 – 1.85 (m, 4H), 1.57 – 1.48 (m, 1H), 1.09 (d, *J* = 7.0 Hz, 3H); ^{13}C NMR (150 MHz, acetone-D_6) δ 195.2, 176.6, 170.6, 170.4, 97.6, 97.6, 84.7, 70.1, 69.5, 68.0, 67.8, 67.1, 66.9, 62.4, 48.3, 40.4, 33.4, 30.6, 30.6, 20.3, 20.6, 18.3, 16.9; HRMS (ESI) calcd. for $C_{27}H_{40}NO_{14}PS_2Na$ (M+Na$^+$) 720.1526 found 720.1515 *m/z*.

3-Hydroxypropyl (6-azidomethyl)nicotinate (SI-7)

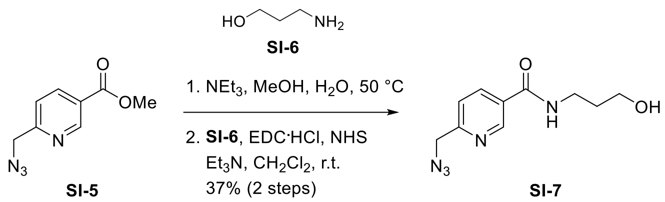

R_f (CH_2Cl_2/MeOH 20:1) = 0.3. ^{1}H NMR (400 MHz, $CDCl_3$) δ 9.28 (d, *J* = 2.3 Hz, 1H), 8.49 (dd, *J* = 8.1, 2.3 Hz, 1H), 7.77 (d, *J* = 8.1 Hz, 1H), 7.44 (br s, 1H), 4.88 (s, 2H), 4.13 (t, *J* = 5.5 Hz, 2H), 3.99 (q, *J* = 5.9 Hz, 2H), 2.70 (br s, 1H), 2.25 – 2.11 (m, 2H); ^{13}C NMR (100 MHz, $CDCl_3$) δ 165.9, 158.8, 147.9, 136.4, 129.4, 121.8, 60.8, 55.4, 38.2, 31.7. HRMS (ESI) calcd. for $C_{10}H_{13}N_5O_2Na$ (M+Na$^+$) 258.0966 found 258.0968 *m/z*.

1,3,4,6-Tri-*O*-acetyl-2-deoxy-2-(5-hexynoyl)amido-αβ-D-glucopyranoside (9)

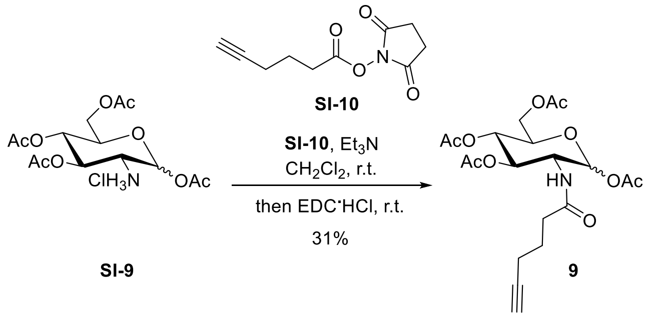

^{1}H NMR (400 MHz, $CDCl_3$) δ 5.88 (d, *J* = 10.2 Hz, 1H), 5.69 (d, *J* = 8.7 Hz, 1H), 5.30 – 5.01 (m, 2H), 4.43 – 4.20 (m, 2H), 4.11 (dd, *J* = 12.5, 2.2 Hz, 1H), 3.83 (ddd, *J* = 9.9, 4.7, 2.2 Hz, 1H), 2.32 – 2.15 (m, 4H), 2.10 (s, 3H), 2.07 (s, 3H), 2.03 (d, *J* = 1.5 Hz, 6H), 1.95 (t, *J* = 2.6 Hz, 1H), 1.77 (p, *J* = 7.0 Hz, 2H). ^{13}C NMR (100 MHz, $CDCl_3$) δ 172.4, 171.3, 170.8, 169.6, 169.5, 92.6, 83.2, 72.9, 72.6, 69.6, 68.1, 61.8, 52.8, 34.9, 24.0, 21.0, 20.8, 20.8, 20.7, 17.6. HRMS (ESI) calcd. for $C_{20}H_{27}NO_{10}Na$ (M+Na$^+$) 464.1532 found 464.1513 *m/z*.

Biotin-PEG$_4$-dialkoxydiphenylsilane-picolyl azide (10)

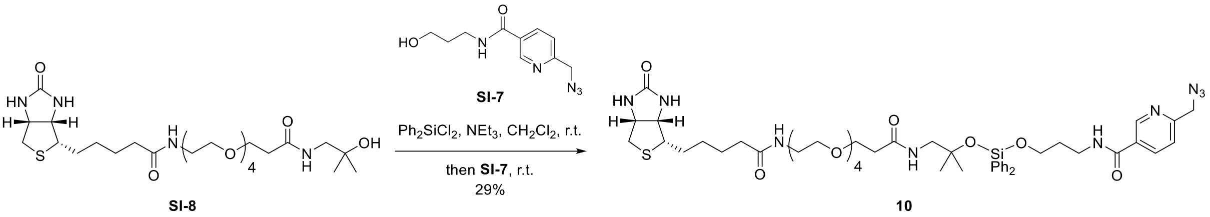

R_f (CH_2Cl_2/MeOH 10:1 with 1% NEt_3) = 0.55. ^1H NMR (600 MHz, $CDCl_3$) δ 8.93 (d, *J* = 2.2 Hz, 1H), 8.05 (dd, *J* = 8.1, 2.3 Hz, 1H), 7.66 – 7.59 (m, 4H), 7.44 – 7.40 (m, 2H), 7.39 – 7.33 (m, 4H), 7.28 (d, *J* = 7.5 Hz, 1H), 7.24 – 7.20 (m, 1H), 6.68 (s, 1H), 6.58 (s, 1H), 5.55 (s, 1H), 4.68 (s, 1H), 4.52 (s, 2H), 4.49 – 4.43 (m, 1H), 4.32 – 4.25 (m, 1H), 3.90 (t, *J* = 5.8 Hz, 2H), 3.70 (t, *J* = 6.1 Hz, 2H), 3.61 – 3.52 (m, 16H), 3.43 – 3.38 (m, 2H), 3.36 (d, *J* = 6.1 Hz, 2H), 3.13 (td, *J* = 7.3, 4.5 Hz, 1H), 2.90 (dd, *J* = 12.8, 5.0 Hz, 1H), 2.69 (d, *J* = 12.7 Hz, 1H), 2.43 (t, *J* = 6.0 Hz, 2H), 2.20 (td, *J* = 7.3, 3.1 Hz, 2H), 1.99 – 1.90 (m, 2H), 1.78 – 1.67 (m, 4H), 1.49 – 1.41 (m, 2H), 1.25 (s, 6H). HRMS (ESI) calcd. for $C_{47}H_{67}N_9O_{10}SSiNa$ (M+Na$^+$) 1000.4399 found 1000.4363 *m/z*.

NMR Spectra

^{1}H NMR, CDCl$_3$, 600 MHz

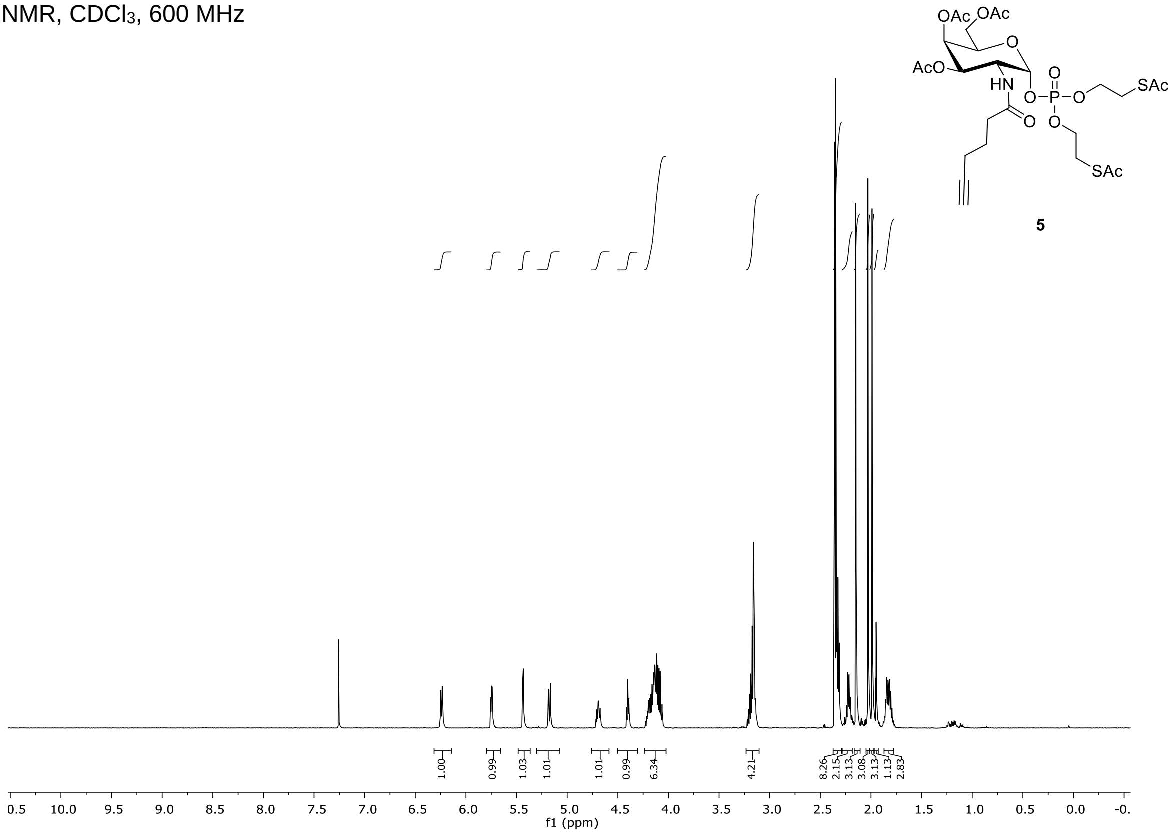

^{13}C NMR, CDCl$_3$, 150 MHz

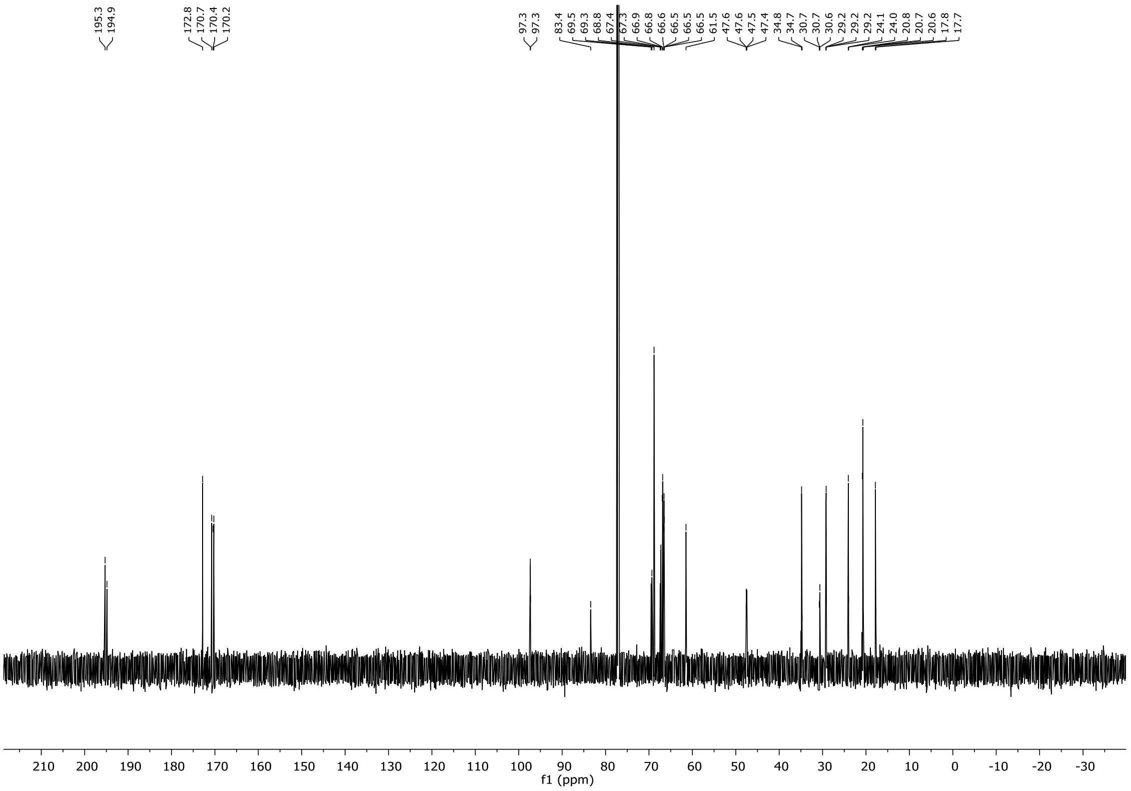

^{1}H NMR, acetone-D_6, 400 MHz

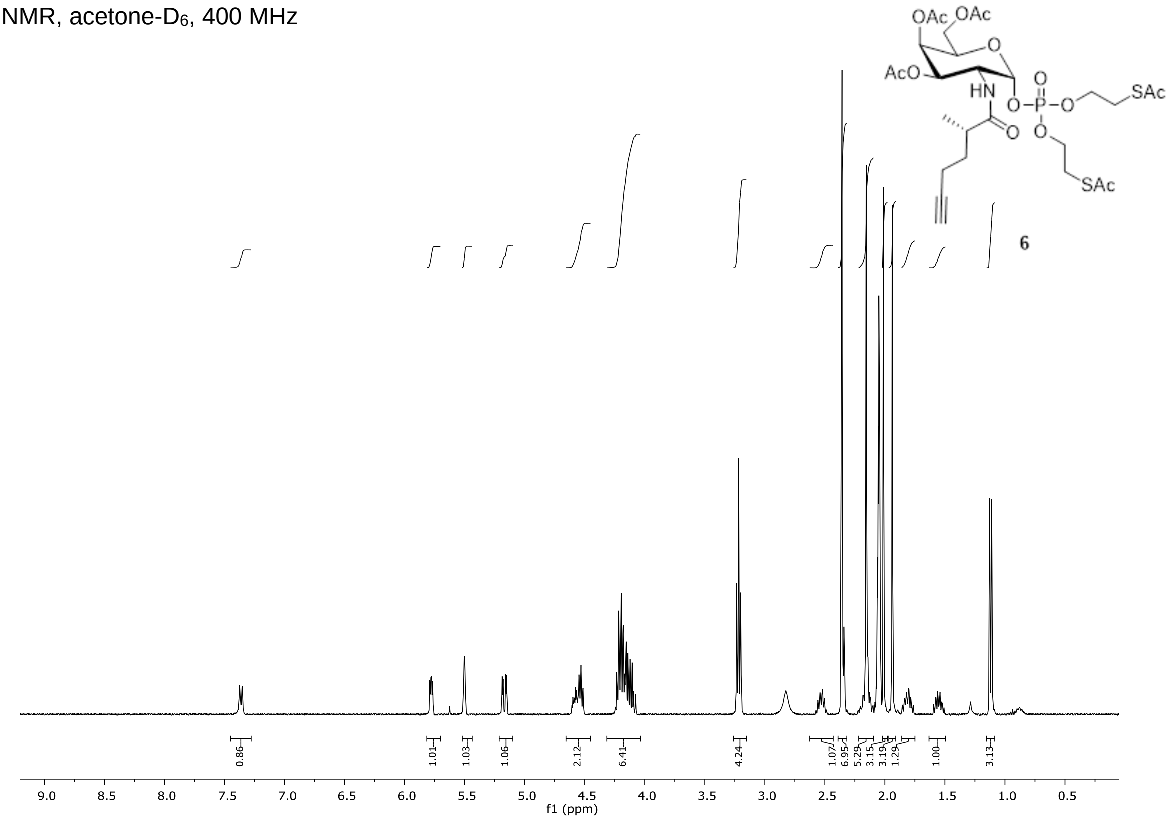

^{13}C NMR, acetone-D_6, 100 MHz

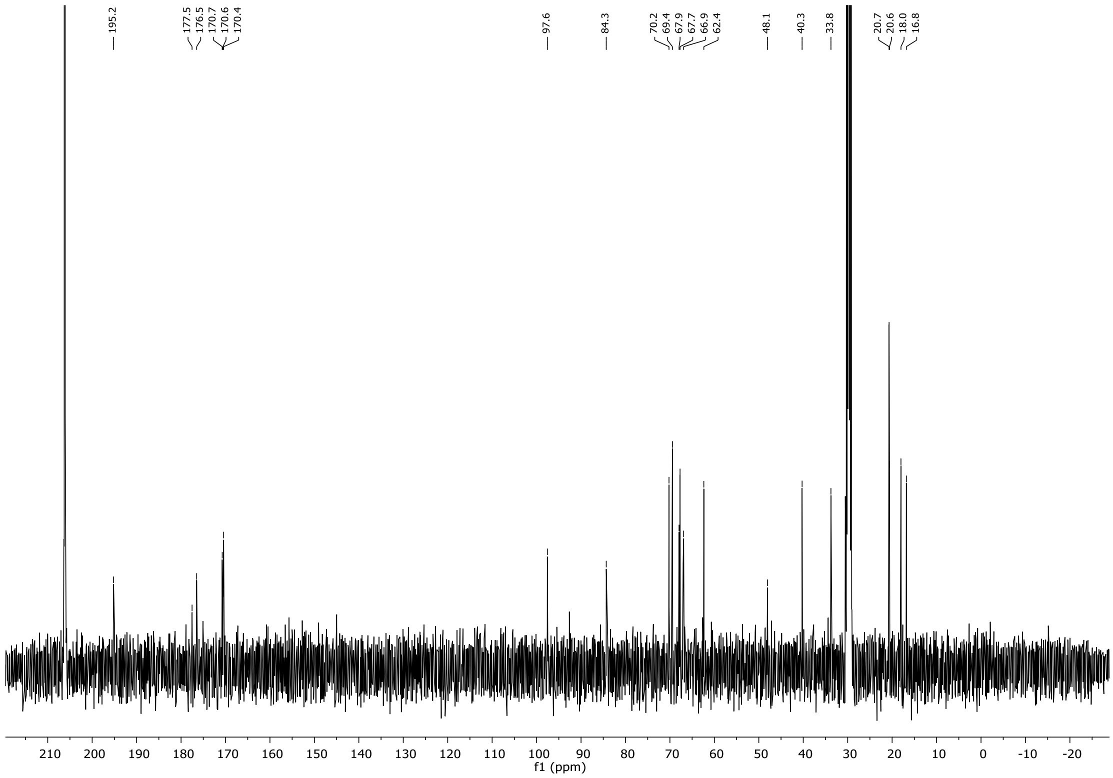

^{1}H NMR, acetone-D_6, 400 MHz

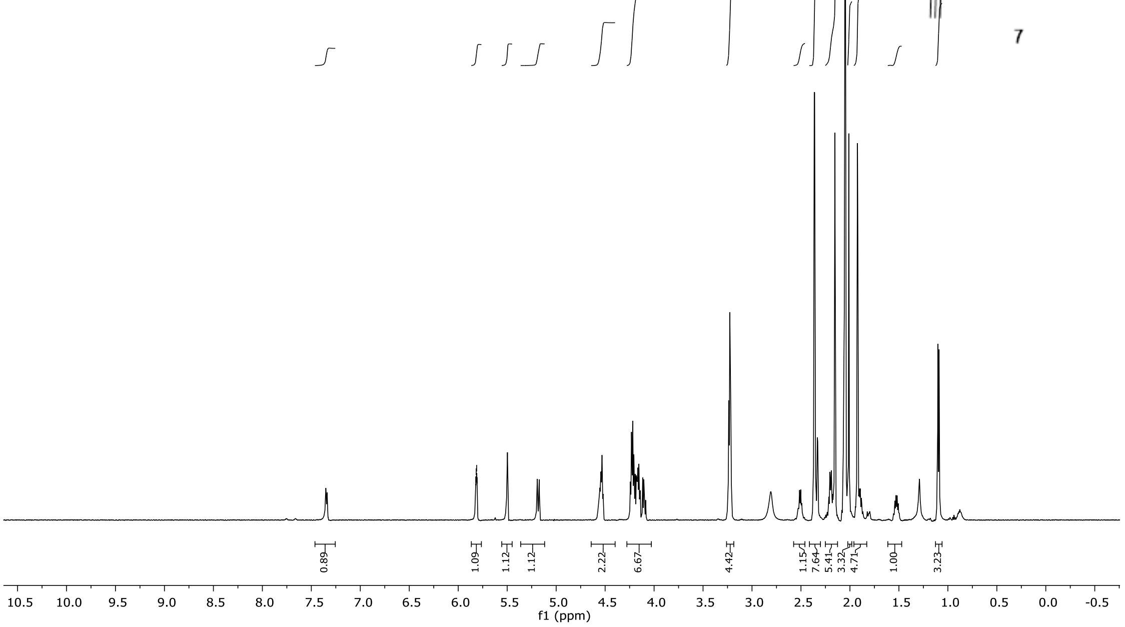

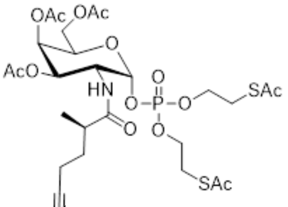

7

^{13}C NMR, acetone-D_6, 100 MHz

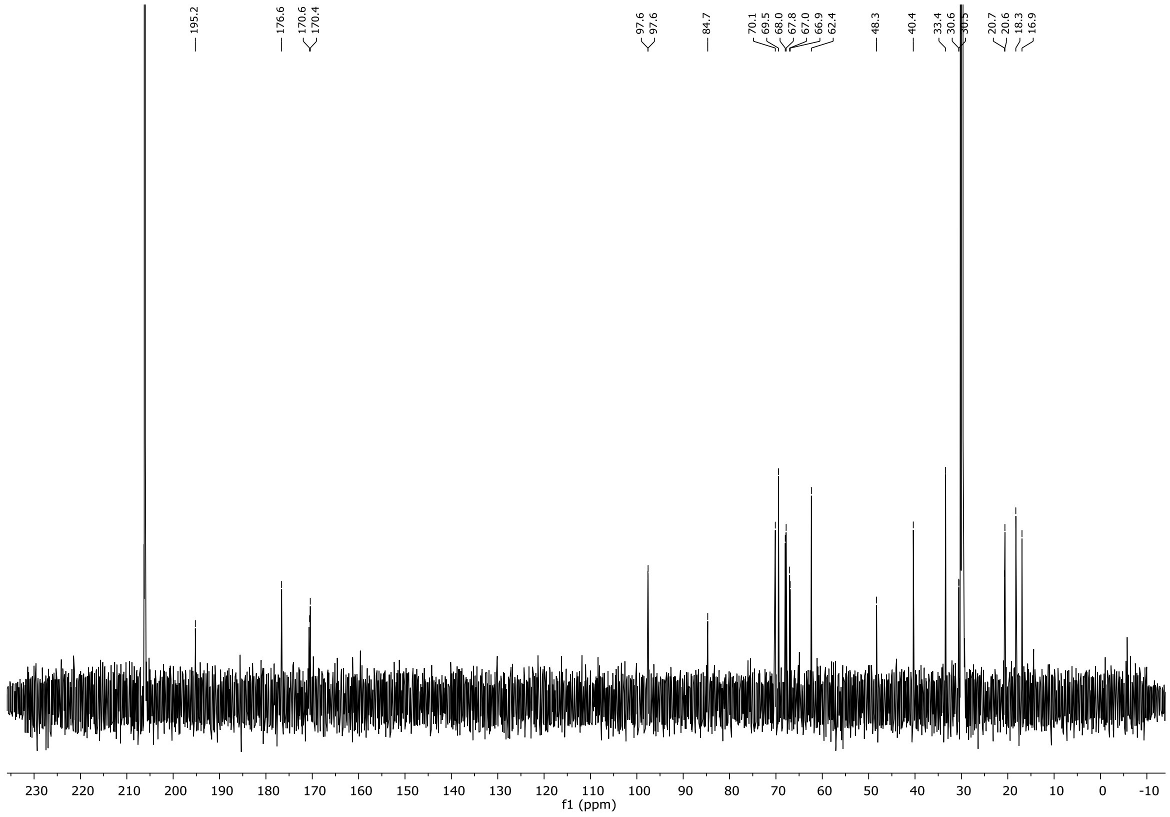

^{1}H NMR, $CDCl_3$, 400 MHz

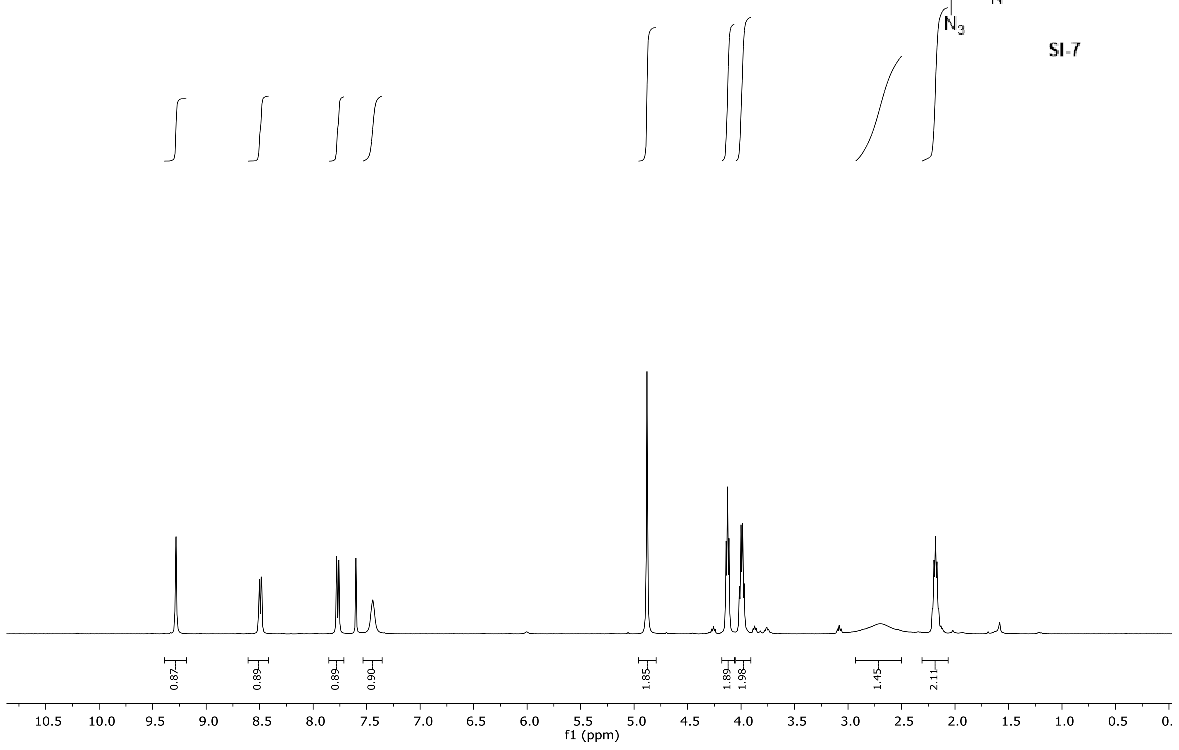

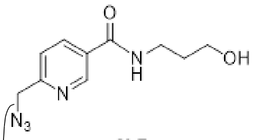

SI-7

^{1}H NMR, $CDCl_3$, 100 MHz

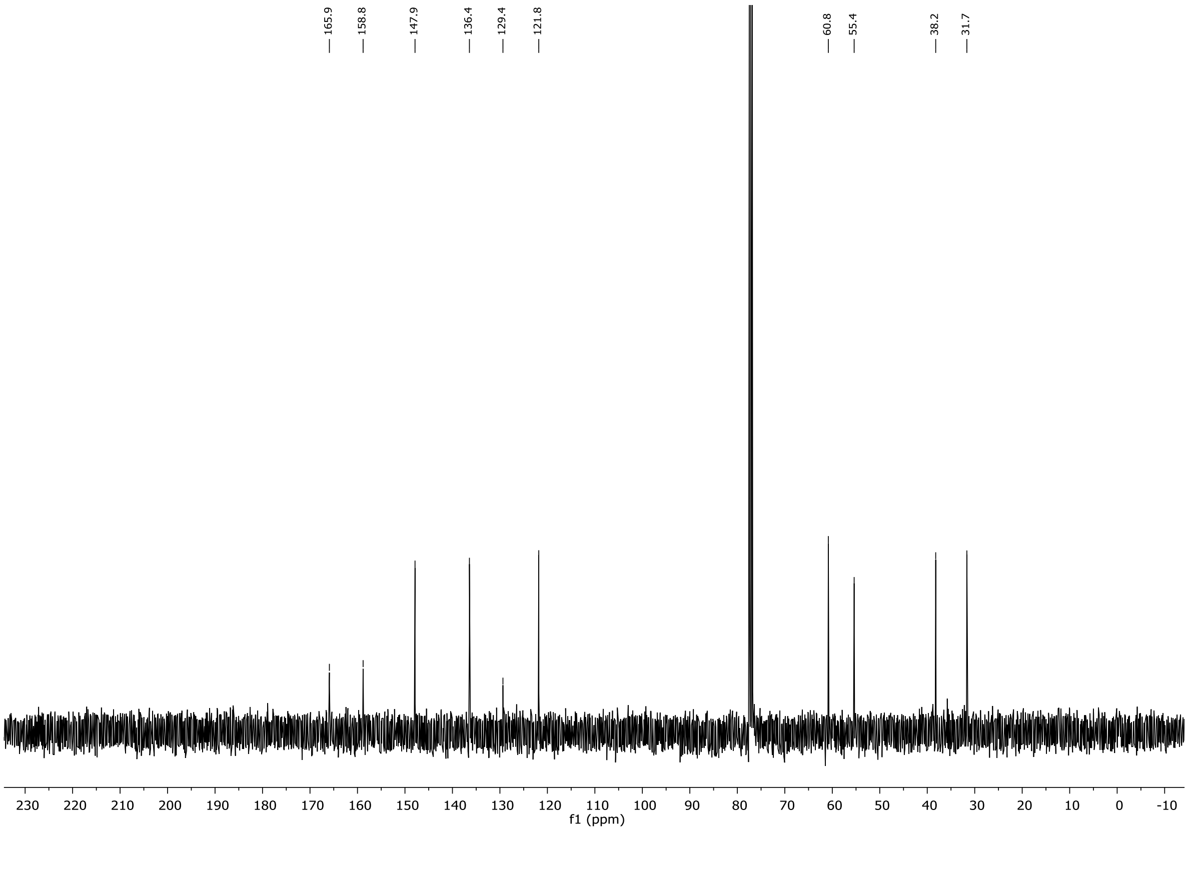

^{1}H NMR, $CDCl_3$, 400 MHz

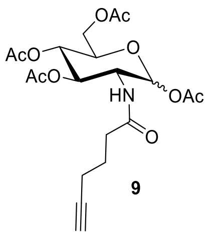

9

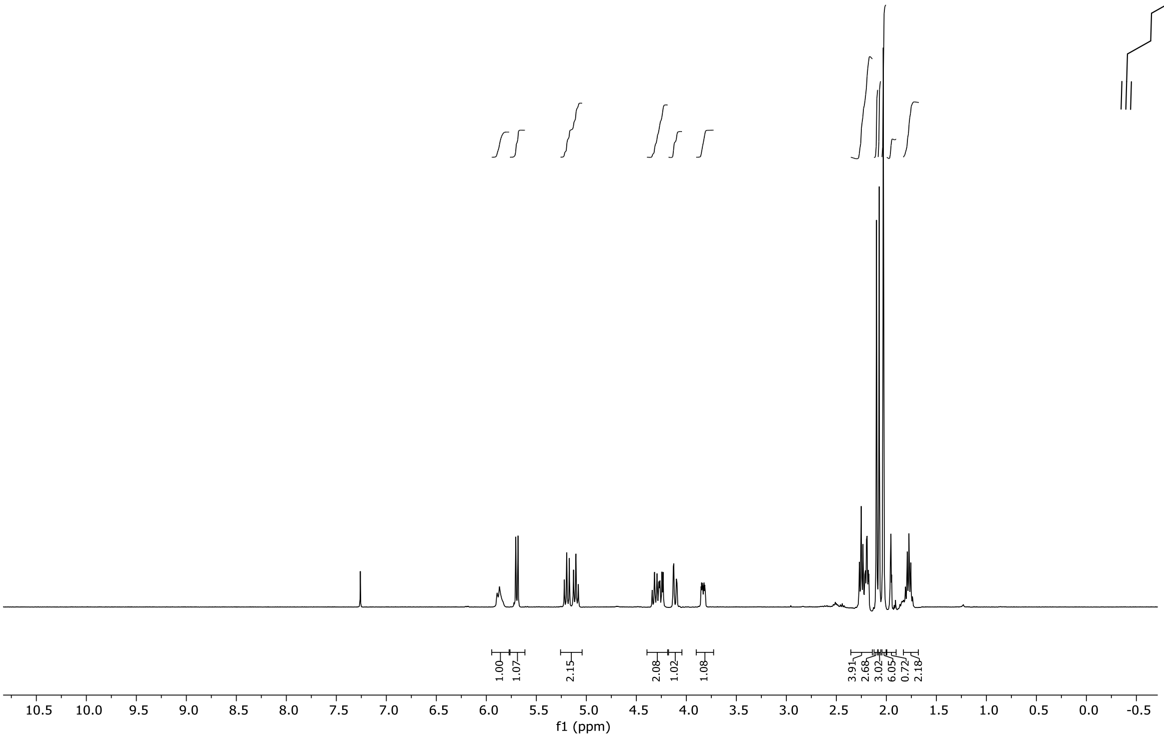

^{1}H NMR, $CDCl_3$, 600 MHz

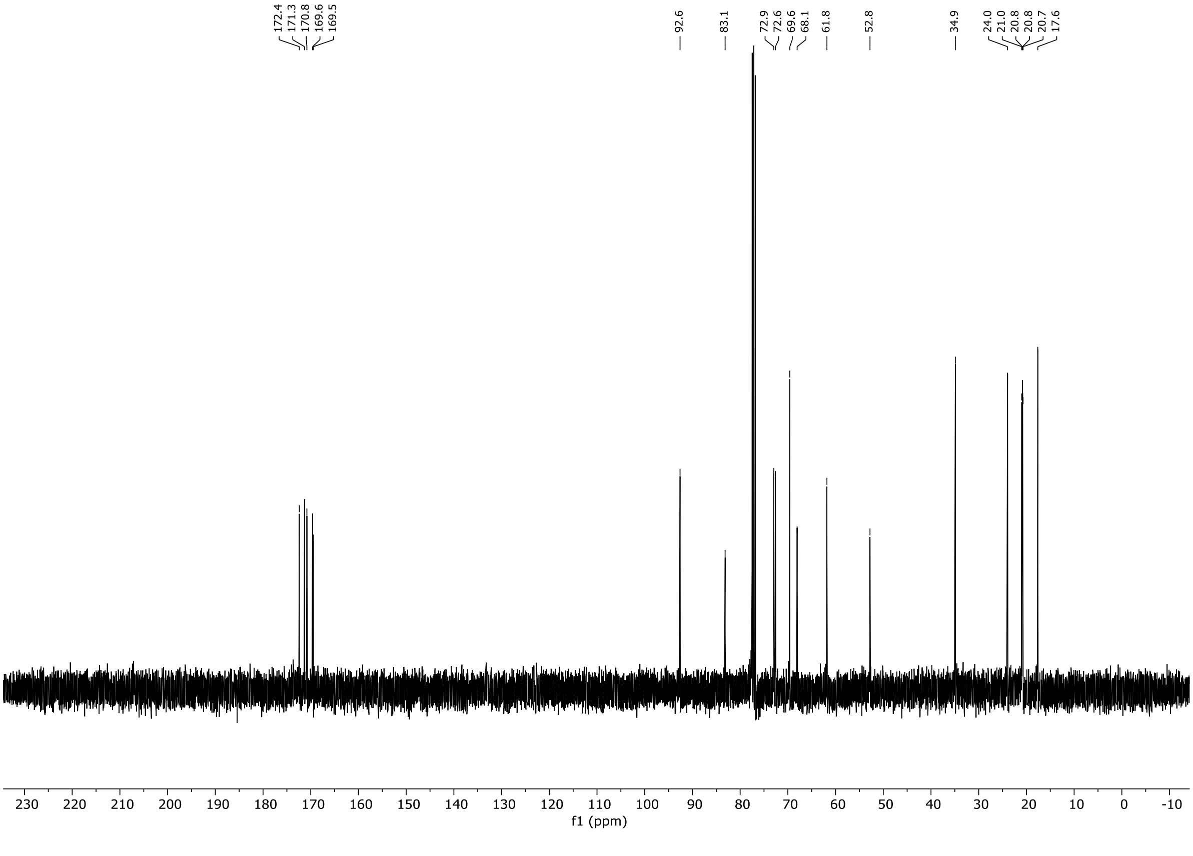

^{1}H NMR, $CDCl_3$, 600 MHz

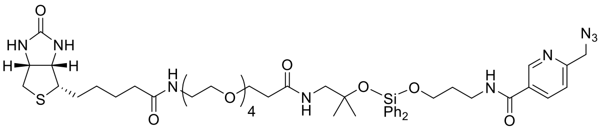

10

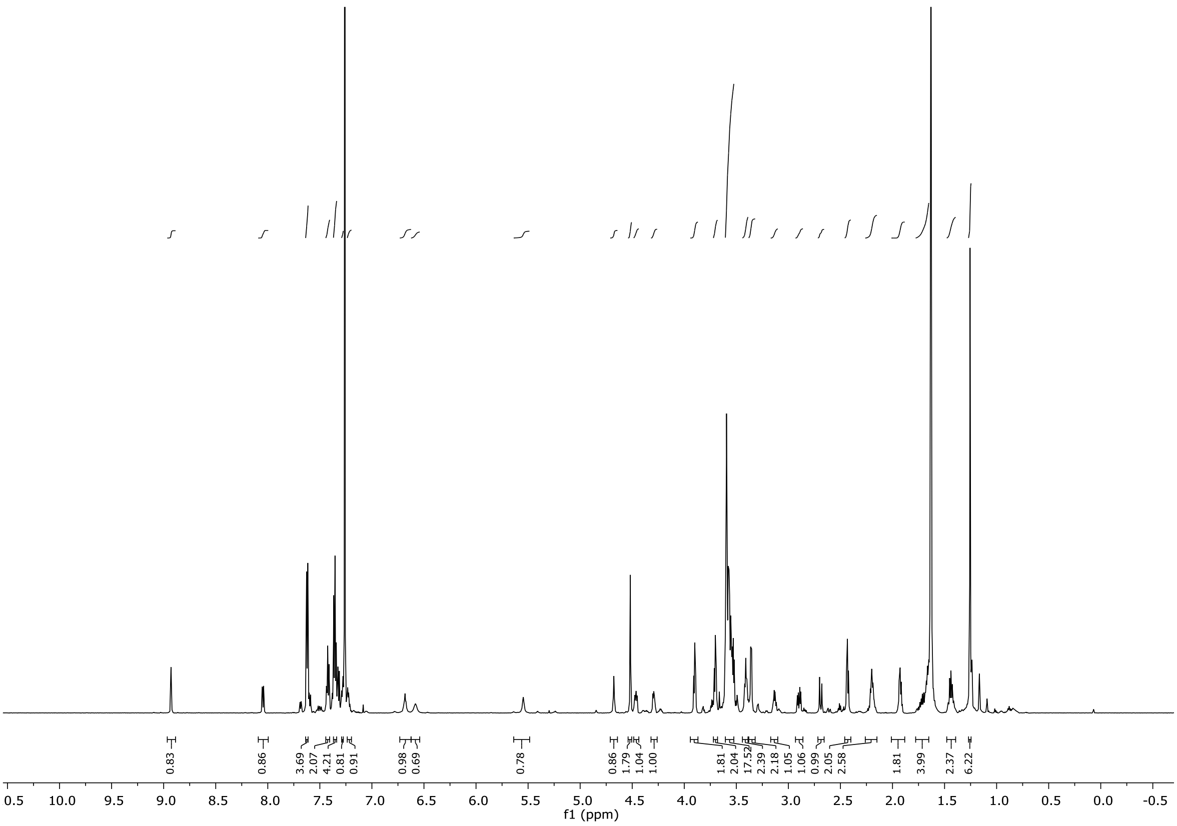

Supplement: Document S1. Figures S1–S5 and Methods S1 [file mmc1.pdf]
