# Supplementary material for: Bump-and-Hole Engineering Identifies Specific Substrates of Glycosyltransferases in Living Cells
Source: Mol Cell. 2020 Jun 4;78(5):824–834.e15. doi: 10.1016/j.molcel.2020.03.030 (PMC7276986; doi:10.1016/j.molcel.2020.03.030)

Molecular Cell

Article

Bump-and-Hole Engineering Identifies Specific Substrates of Glycosyltransferases in Living Cells

Graphical Abstract

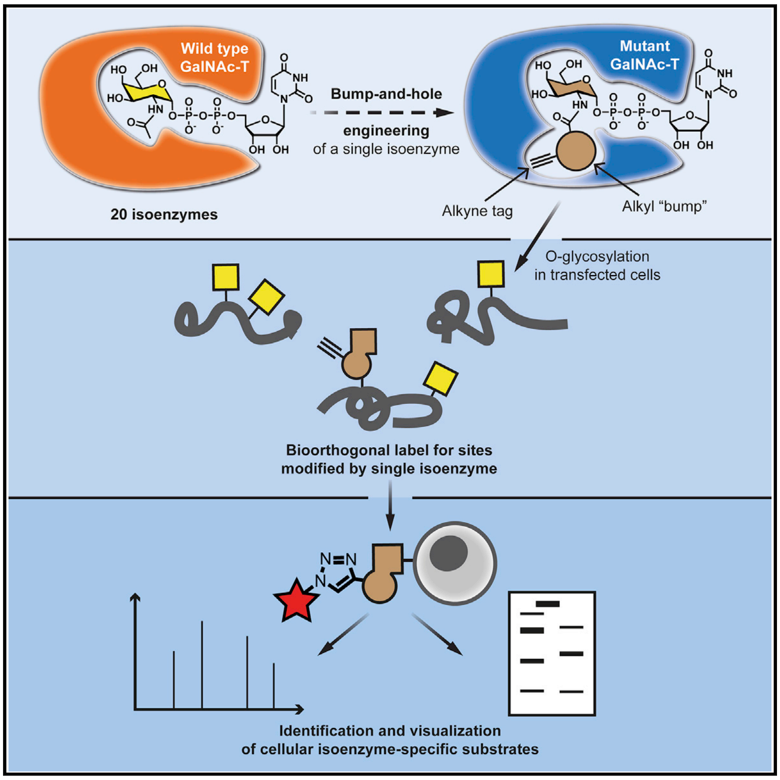

Authors

Benjamin Schumann, Stacy Alyse Malaker, Simon Peter Wisnovsky, ..., Jennifer Jean Kohler, Milan Mrksich, Carolyn Ruth Bertozzi

Correspondence

b.schumann@imperial.ac.uk (B.S.), bertozzi@stanford.edu (C.R.B.)

In Brief

Schumann et al. establish a cellular glycosyltransferase bump-and-hole system to chemically tag the substrates of individual isoenzymes of the GalNAc-T family.

Highlights

- GalNAc-T glycosyltransferase bump-and-hole engineering retains structure and substrate specificity
- Bumped UDP-GalNAc analogs can be delivered to the living cell
- Cellular substrates of individual GalNAc-Ts can be bioorthogonally tagged
- Glycoproteomics identifies GalNAc-T-specific glycosylation sites

Schumann et al., 2020, Molecular Cell *78*, 824–834
June 4, 2020 © 2020 The Author(s). Published by Elsevier Inc.
https://doi.org/10.1016/j.molcel.2020.03.030

CellPress

CellPress

Molecular Cell

Article

Bump-and-Hole Engineering Identifies Specific Substrates of Glycosyltransferases in Living Cells

Benjamin Schumann,[1,2,3,9,*] Stacy Alyse Malaker,[1] Simon Peter Wisnovsky,[1] Marjoke Froukje Debets,[1,11] Anthony John Agbay,[1] Daniel Fernandez,[4] Lauren Jan Sarbo Wagner,[5] Liang Lin,[6] Zhen Li,[2,3] Junwon Choi,[1,12] Douglas Michael Fox,[1] Jessie Peh,[1] Melissa Anne Gray,[1] Kayvon Pedram,[1] Jennifer Jean Kohler,[7] Milan Mrksich,[6] and Carolyn Ruth Bertozzi[1,8,10,13,*]

[1]Department of Chemistry, Stanford University, Stanford, CA 94305, USA
[2]Chemical Glycobiology Laboratory, The Francis Crick Institute, NW1 1AT London, United Kingdom
[3]Department of Chemistry, Imperial College London, W12 0BZ London, United Kingdom
[4]Stanford ChEM-H Macromolecular Structure Knowledge Center, Stanford, CA 94305, USA
[5]Department of Chemistry, University of California, Berkeley, CA 94720, USA
[6]Department of Biomedical Engineering, Northwestern University, Evanston, IL 60208, USA
[7]Department of Biochemistry, University of Texas Southwestern Medical Center, Dallas, TX 75390, USA
[8]Howard Hughes Medical Institute, Stanford, CA 94305, USA
[9]Twitter: @DrGlycoBen
[10]Twitter: @CarolynBertozzi
[11]Present address: Lilly Research Laboratories, Eli Lilly and Company, Indianapolis, IN 46285, USA
[12]Present address: Korea Institute of Science and Technology, Hwarangro 14-gil 5, Seongbuk-gu, Seoul, 02792, Republic of Korea
[13]Lead Contact
*Correspondence: b.schumann@imperial.ac.uk (B.S.), bertozzi@stanford.edu (C.R.B.)
https://doi.org/10.1016/j.molcel.2020.03.030

SUMMARY

Studying posttranslational modifications classically relies on experimental strategies that oversimplify the complex biosynthetic machineries of living cells. Protein glycosylation contributes to essential biological processes, but correlating glycan structure, underlying protein, and disease-relevant biosynthetic regulation is currently elusive. Here, we engineer living cells to tag glycans with editable chemical functionalities while providing information on biosynthesis, physiological context, and glycan fine structure. We introduce a non-natural substrate biosynthetic pathway and use engineered glycosyltransferases to incorporate chemically tagged sugars into the cell surface glycome of the living cell. We apply the strategy to a particularly redundant yet disease-relevant human glycosyltransferase family, the polypeptide *N*-acetylgalactosaminyl transferases. This approach bestows a gain-of-chemical-functionality modification on cells, where the products of individual glycosyltransferases can be selectively characterized or manipulated to understand glycan contribution to major physiological processes.

INTRODUCTION

Posttranslational modifications expand the structural diversity of proteins but are notoriously difficult to study in living systems. Most modifications are refractory to direct genetic manipulation and require reductionist strategies such as *in vitro* systems or simplified cells. Glycans are the prime example for this; the human glycome is constructed by the combinatorial activity of more than 250 glycosyltransferases (GTs) with both hierarchical and competing activities. On the cell surface, glycans play a central role in modulating signal transduction, cell-cell interactions, and biophysical properties of the plasma membrane (Varki, 2017; Varki et al., 2017). Yet, we still lack the methodology to selectively visualize, modify, or sequence either a certain glycan subtype or the product of a certain GT. In a synthetic biology approach, individual GTs could be engineered to accommodate a chemical-functionality that is not found in native substrates and not accommodated by other GTs. This "bump-and-hole" tactic has been applied to a range of enzymes, including but not limited to kinases, methyl transferases, and ADP-ribosyltransferases (Besanceney-Webler et al., 2011; Alaimo et al., 2001; Carter-O'Connell et al., 2014; Gibson et al., 2016; Islam et al., 2011, 2018). We have recently developed the first GT bump-and-hole system that was applicable to multiple members of a GT family *in vitro* (Choi et al., 2019). However, application in the living cell has always been a substantial technical challenge for most bump-and-hole-systems; the nucleotide-based substrate analog must be delivered across the plasma membrane and into the Golgi compartment, and the cell must stably express the correctly localized and folded mutant enzyme. Bump-and-hole engineering is particularly attractive to deconvolve GT families of multiple homologous isoenzymes, as the complex interplay of these isoenzymes in the secretory pathway cannot be probed in sufficient detail in *in vitro* assays.

 © 2020 The Author(s). Published by Elsevier Inc.
This is an open access article under the CC BY license (http://creativecommons.org/licenses/by/4.0/).

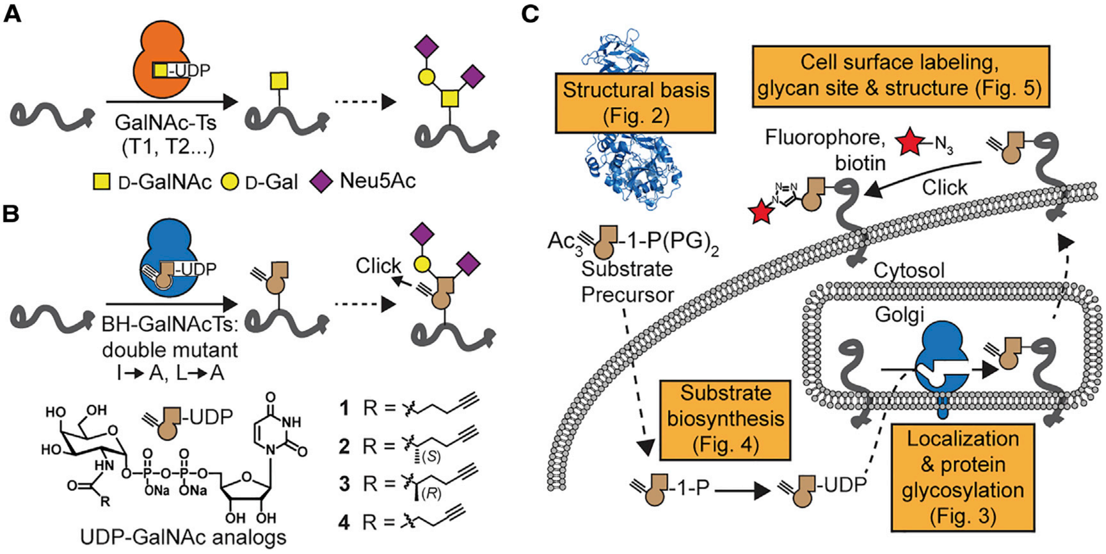

Figure 1. GalNAc-T Bump-and-Hole Engineering

(A) GalNAc-Ts initiate O-GalNAc glycosylation. Transfer of GalNAc to a Ser or Thr side chain is followed by downstream glycan elongation.
(B) The principle of bump-and-hole engineering. Engineered double-mutant (BH) GalNAc-Ts are paired with UDP-GalNAc analogs **1**–**4** to chemically tag GalNAc-T substrates that can be monitored by click chemistry.
(C) Overview of the steps taken in this study toward GalNAc-T bump-and-hole engineering in the living cell.
PG, protecting group.

One of the largest GT families in the human genome is the polypeptide *N*-acetylgalactosaminyl (GalNAc) transferase family (GalNAc-T1…T20, abbreviated T1…T20). Transferring GalNAc to Ser/Thr side chains, GalNAc-Ts initiate abundant O-linked glycosylation in the secretory pathway (Figure 1A) (Bennett et al., 2012; Hang and Bertozzi, 2005). O-GalNAc glycosylation can differ based on cell type or activation stage, and clear disease phenotypes are associated with glycan aberration (Antonopoulos et al., 2012; Radhakrishnan et al., 2014). Unsurprisingly, GalNAc-T expression is often associated with tumorigenesis, sometimes with opposite effects on different types of cancer (Peng et al., 2012; Gaziel-Sovran et al., 2011). However, the absence of a glycosylation consensus sequence and the variability of glycan elaboration render O-GalNAc glycans challenging to study by mass spectrometry (MS)-based glycoproteomics. Thus, the protein substrate specificity of each isoenzyme is poorly studied and largely built on data inferred from synthetic peptide libraries (de Las Rivas et al., 2019). So-called SimpleCells that do not elaborate O-GalNAc glycans make glycoproteins that are easier to enrich and study by MS (Steentoft et al., 2011, 2013; Schjoldager et al., 2012; Narimatsu et al., 2019). Knock-outs (KOs) of single GalNAc-Ts in the SimpleCell background have been profiled by glycoproteomics (Schjoldager et al., 2012, 2015). Similarly, glycopeptides from cells with wild-type (WT) O-glycan elaboration and titratable GalNAc-T knockin have been enzymatically simplified to allow for enrichment and MS (Hintze et al., 2018). These studies have revealed comprehensive glycoproteomics datasets but suffer from a loss of glycan elaboration and laborious genome engineering required for targeted knockin. Further, the activity of GalNAc-T isoenzymes is both redundant and competitive, such that the compensation and/or shift of glycosylation sites occurs upon KO (Schjoldager et al., 2015; Hintze et al., 2018). A gain-of-chemical-functionality strategy to visualize the products of a particular GT on the living cell is currently unavailable.

Here, we equipped living cells with the ability to tag the protein substrates of individual GalNAc-Ts with chemical, editable functionalities. We made use of the fact that a double mutation ("BH," I253A/L310A double mutant in GalNAc-T2; Figure 1B) re-programmed GalNAc-Ts to accept alkyne-containing uridine diphosphate (UDP)-GalNAc analogs such as compounds **1**–**4** instead of the native substrate UDP-GalNAc (Figure 1B; Choi et al., 2019). We endowed living cells with the capacity to biosynthesize a UDP-GalNAc analog that was complementary to engineered BH-GalNAc-Ts. Further, we showed that BH-GalNAc-Ts emulate WT-GalNAc-Ts with regard to structure, localization, and protein substrate specificity. This approach bestowed a bioorthogonal tag (Sletten and Bertozzi, 2009; Prescher et al., 2004; Hang et al., 2003; Zaro et al., 2011; Woo et al., 2015) on the protein substrates of distinct GalNAc-T isoenzymes while displaying the complexity of glycan elaboration in the secretory pathway (Figure 1C). Precision glycome engineering has widespread applications in biomarker discovery, GT profiling, and targeted cell surface engineering.

RESULTS

Structural Basis for GalNAc-T Engineering

As bump-and-hole engineering of a GT family in living cells has no precedent, we first set out to understand the structural implications of this approach. All GalNAc-Ts are type II transmembrane proteins with luminal GT and lectin domains connected through a flexible linker (Bennett et al., 2012; Hang and Bertozzi, 2005; Ten Hagen et al., 2003). We crystallized the luminal part of BH-T2 in complex with the native ligands Mn^{2+}, UDP, and the substrate peptide EA2 (PTTDSTTPAPTTK) at 1.8-Å resolution.

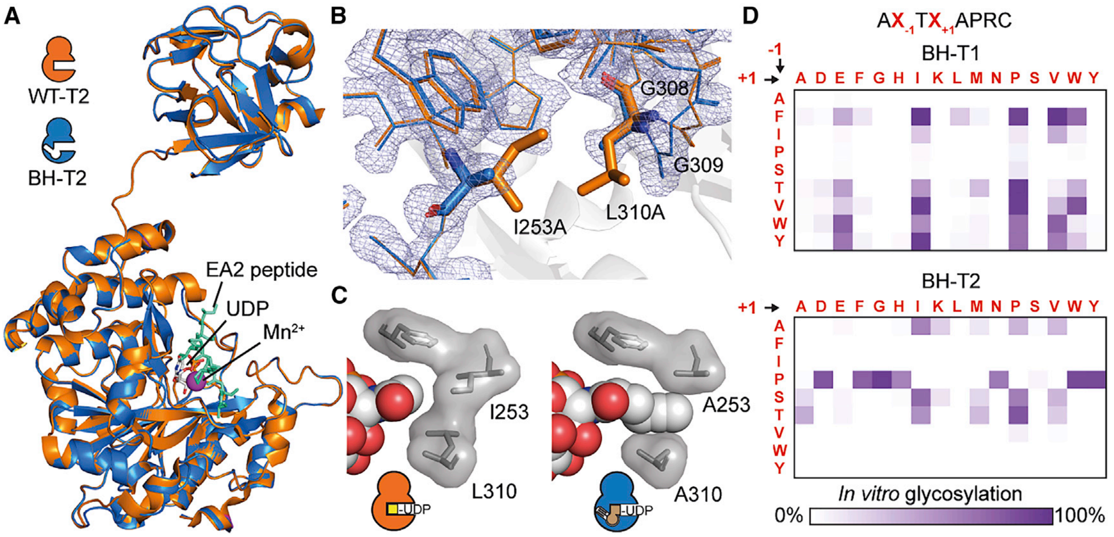

Figure 2. Bump-and-Hole Engineering Conserves Folding and Substrate Binding of GalNAc-T2

(A) Crystal structure of BH-T2 at 1.8 Å superposed with WT-T2 (PDB: 2FFU). Bound EA2 substrate peptide is cyan (sticks), Mn^{2+} is magenta (sphere), and UDP is gray (sticks). Ligands are taken from BH-T2. For superposition with WT-T2 ligands, see Figure S1A.

(B) Superposition of the UDP-sugar binding site of BH-T2 and WT-T2. Electron density is rendered at 1 σ and carved at 1.6 Å.

(C) Depiction of UDP-GalNAc analog **1** in a co-crystal structure with BH-T2 at 3.1 Å and UDP-GalNAc in a co-crystal structure with WT-T2 (PDB: 4D0T) (Lira-Navarrete et al., 2014), as well as WT and mutated gatekeeper residues.

(D) Substrate specificities of BH-T1 and BH-T2 as determined in an *in vitro* glycosylation assay with detection by SAMDI-MS. For comparison with WT-GalNAc-T glycosylation, see Figure S1. Data are from one representative out of two independent experiments.

See also Figure S1D and Table 1.

Comparison of BH-T2 and WT-T2 (PDB: 2FFU) revealed complete conservation of both the three-dimensional enzyme architecture and bound ligand structure (Figures 2A and S1A–S1C; Table 1; Fritz et al., 2006). In BH-T2, the interdomain linker adopts an extended conformation previously found in the catalytically active WT enzyme (Lira-Navarrete et al., 2014, 2015). The mutant A253 and A310 side chains in the BH enzyme are congruent with the WT side chains I253 and L310, respectively (Figure 2B). Two glycine residues, G308 and G309, are slightly shifted by 1.2 Å and 1.7 Å (C_α distances), respectively, likely to account for the changes elsewhere in the active site. BH-T2 thus retains the native structural properties of the WT-GalNAc-T enzyme.

A co-crystal structure of BH-T2, Mn^{2+}, and UDP-GalNAc analog **1** at 3.1-Å resolution helped us visualize how the BH-T2 active site mutations affect enzyme-substrate binding. In comparison with a corresponding WT-T2/UDP-GalNAc/Mn^{2+}/EA2 complex (PDB: 4D0T), UDP-sugar binding is completely conserved (Figures S1B and S1C; Table S1; Lira-Navarrete et al., 2014). BH-T2 indeed contains a hole that accommodates the alkyne side chain bump in UDP-GalNAc analog **1** (Figure 2C). The formation of additional van der Waals interactions between the enzyme and substrate explain why the K_M of BH-T2 toward **1** (2.6 μM) is approximately 10-fold lower than the K_M of WT-T2 toward UDP-GalNAc (30 μM) (Choi et al., 2019). In turn, the fact that the k_{cat} of BH-T2/**1** (0.158 s^{-1}) is approximately 5-fold lower than the k_{cat} of WT-T2/UDP-GalNAc (0.813 s^{-1}) indicates that these additional interactions may slightly slow the ring distortion that is needed to mediate the nucleophilic attack (Lira-Navarrete et al., 2014).

To corroborate our structural interpretation that GalNAc-T bump-and-hole engineering does not substantially alter substrate peptide binding, we profiled the substrate specificities of two engineered GalNAc-Ts: BH-T1 and BH-T2 (Choi et al., 2019; Gerken et al., 2006, 2011; Ji et al., 2018; Revoredo et al., 2016). A peptide library containing a single Thr residue with randomized neighboring residues was used in *in vitro* glycosylation experiments and analyzed by self-assembled monolayers for matrix-assisted desorption/ionization MS (SAMDI-MS; Figures 2D and S1D; Kightlinger et al., 2018). We found differences in substrate specificity between BH-T1 and BH-T2 that were similar to the differences seen in the corresponding WT-GalNAc-Ts: T1 generally preferred hydrophobic amino acids at −1 position and Glu, Ile, Pro, Val, or Trp at +1 position of the Thr glycan acceptor, whereas T2 showed a preference for Pro, Ala, Ser, and Thr at −1 position (Figure S1D; Kightlinger et al., 2018). We note that BH-GalNAc-Ts glycosylated fewer peptides than the corresponding WT enzymes; we therefore repeated the experiment on a smaller peptide subset that allowed a more detailed view on peptide substrate fine specificities and found that glycosylation profiles by BH-GalNAc-Ts matched the corresponding WT-GalNAc-Ts' profiles (Figure S1D). These data indicate that bump-and-hole engineering faithfully reports on GalNAc-T isoenzyme activity *in vitro* with simple peptide substrates containing a single acceptor Thr, a crucial prerequisite for a cellular bump-and-hole system.

Table 1. Crystallographic Data Statistics

Parameters	BH-GalNAc-T2 with EA2 and UDP	BH-GalNAc-T2 with UDP-GalNAc analog 1
PDB	6E7I	6NQT
Unit Cell Constants		
a, *b* (Å)	69.31	116.58, 120.13
c (Å)	169.78	247.39
α, β, γ (°)	90, 90, 120	90, 90, 90
Resolution range (Å)	56.7-1.80	39.0-3.05
Space group	$P6_1$ (1 mol/ASU)	$P\ 2_1\ 2_1\ 2_1$ (6 mols/ASU)
Wavelength (Å)/ synchrotron source	0.9774/ALS BL5.0.1	0.9753/SSRL BL7-1
Number of measured/ unique reflections	230,556/39,854	286,630/64,645
R_{merge}[a] (%)	10.7 (47.1)	13.5 (77.1)
Completeness (%) and multiplicity	93.6 (93.7)/ 5.8 (5.6)	97.1 (98.3)/3.0 (3.0)
Mean I/σI	9.5 (0.7)	7.7 (1.6)
Mean I half-set correlation coefficient $CC_{1/2}$	0.994 (0.674)	0.989 (0.646)
Refinement Statistics		
Reflections used, total/test set	37,096/1,998	61,083/3,345
Crystallographic R_{factor}[b] R_{free}[c]	16.7/21.6	23.1/28.6
rmsd bond lengths (Å)	0.013	0.016
rmsd bond angles (°)	1.61	2.06
Number of protein atoms/total atoms	3,899/4,314	22,685/23,138
B-Factor Statistics ($Å^2$)		
Overall/Wilson *B*-factor	16.5/11.8	62.9/63.8
Protein, average by chain ID	17.0 (chain A)	54.0 (A), 63.7 (B), 62.7 (C), 62.8 (D), 74.1 (E), 82.1 (F)
Ligand, average by chain ID	peptide (62 atoms): 17.6	butyne (43 atoms): 50.5 (A) 64.9 (B), 57.1 (C), 59.6 (D), 72.7 (E), 77.7 (F)
	UDP (25 atoms): 10.9	manganese (1 atom): 49.3 (A) 30.6 (B), 48.7 (C), 50.8 (D), 47.7 (E), 73.2 (F)
	manganese (1 atom): 10.1	
Solvent/other atoms	22.0 (327 waters)	34.2 (189 waters)
Ramachandran Statistics Protein Geometry[d]		
Most favored and additional allowed (%)	99.3 (434 of 437 non-proline non-glycine residues)	96.4 (2,475 of 2,567 non-proline non-glycine residues)
Generously allowed (%)	0.2 (1 residue)	2.2 (56 residues)
Outliers (%)	0.5 (2 residues)	1.4 (36 residues)
PDB ID	6E7I	6NQT

Figures in parentheses relate to the outer shell.

[a]$R_{merge} = \Sigma_{hkl} \Sigma_{j=1}$ to $N \mid I_{hkl} - I_{hkl}(j) \mid / \Sigma_{hkl} \Sigma_{j=1}$ to $N\, I_{hkl}(j)$, where N is the redundancy of the data. In parentheses, outermost shell statistics at these limiting values: 1.85–1.80 Å in GalNac T2 with EA2 and UDP and 3.21–3.05 Å in GalNAc-T2 UDP-GalNAc analog 1.

[b]$R_{factor} = \Sigma_{hkl} ||F_{obs}| - |F_{calc}|| / \Sigma_{hkl} |F_{obs}|$, where the F_{obs} and F_{calc} are the observed and calculated structure factor amplitudes of reflection hkl.

[c]R_{free} is equal to R_{factor} for a randomly selected 5.0% subset of the total reflections that were held aside throughout refinement for cross-validation.

[d]According to Procheck.

BH-GalNAc-Ts Glycosylate Membrane Proteins

We next sought to confirm that BH-GalNAc-Ts localize to the Golgi compartment and glycosylate proteins in a membrane environment. Full-length WT- and BH-T1 and T2 with a C-terminal vesicular stomatitis virus G protein (VSV-G) epitope tag exhibited doxycycline (Dox) dose-dependent expression under the control of a tetracycline-responsive promoter in stably transfected HepG2 cells (Figures 3A and S2A; Kowarz et al., 2015). All tested GalNAc-Ts co-localized with the Golgi marker giantin, confirming the native localization of engineered GalNAc-Ts (Figures 3B and S2B). To assess GalNAc-T activity, membrane protein fractions were prepared from WT- and BH-GalNAc-T-expressing HepG2 cells and used for *in vitro* glycosylation experiments. After incubation with alkyne-containing UDP-GalNAc analog **1**, alkyne-tagged glycoproteins were derivatized with azide-biotin using Cu^I-catalyzed [3+2] "click" cycloaddition and characterized on a streptavidin blot. Compounds **1**, **2**, and **3** were specific substrates for BH- but not WT-GalNAc-Ts. In the presence of these substrates, BH-T1 and BH-T2 labeled overlapping and also unique glycoprotein species (Figures 3C, S2C, and S2D). In contrast, treatment of lysates from WT-T1- and WT-T2-expressing cells with compound **4** with a shorter alkyne chain led to substantial labeling, consistent with **4** being a substrate for WT-GalNAc-Ts (Choi et al., 2019; Zaro et al., 2011). These results were confirmed using soluble GalNAc-Ts and a membrane fraction of non-transfected cells (Figure S2E). Importantly, profiling both enzyme activity and protein specificity of single GalNAc-T isoenzymes has been impossible to date even in cell lysates. We next targeted a GalNAc-T bump-and-hole platform in the living cell.

Biosynthesis of a Bumped UDP-GalNAc Analog

Among the insightful bump-and-hole studies that have probed various enzyme families, few have been performed in living cells due to the inability to deliver negatively charged substrates across the plasma membrane (Carter-O'Connell et al., 2014; Gibson et al., 2016; Islam, 2018; Wang et al., 2013). GalNAc analogs have been fed to cells as membrane-permeable per-acetylated precursors that are deprotected by esterases and converted to UDP-GalNAc analogs via the kinase GALK2 and the pyrophosphorylase AGX1 (Figure 4A; Zaro et al., 2011; Woo et al., 2015; Boyce et al., 2011; Pouilly et al., 2012). By delivery of the corresponding protected GalNAc-1-phosphate analog, a GalNAc analog can bypass GALK2, such that only AGX1 is necessary for biosynthesis to the UDP-GalNAc analog. However, in accordance with previous findings (Pouilly et al.,

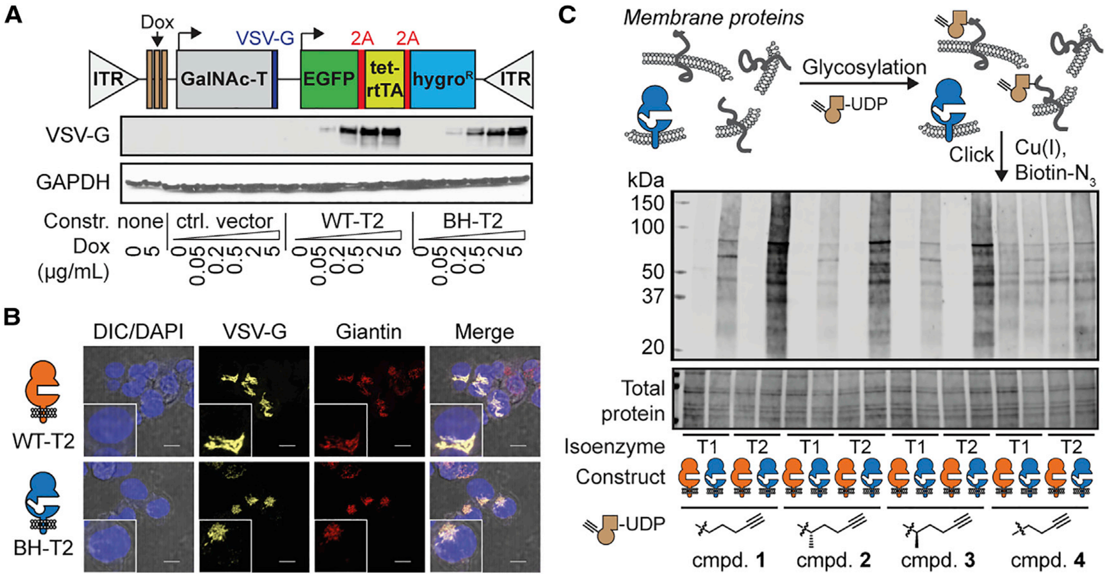

Figure 3. Engineered GalNAc-Ts Localize to the Golgi Compartment and Glycosylate Protein Substrates

(A) Expression construct for full-length GalNAc-Ts under the control of a Dox-inducible promoter. Inverted tandem repeats (ITRs) are recognized by Sleeping Beauty transposase. WT-T2 and BH-T2 were expressed by stably transfected HepG2 cells in a Dox-inducible fashion.

(B) Fluorescence microscopy of HepG2 cells stably transfected with T2 constructs, induced with 0.2 µg/mL Dox, and subsequently stained. Inset: magnification of a single cell. Scale bar, 10 µm.

(C) *In vitro* glycosylation of proteins in a membrane fraction by full-length GalNAc-Ts using UDP-GalNAc analogs. Data are from one representative out of two independent experiments. Experiments were repeated with the membrane fraction of non-transfected cells and soluble, purified GalNAc-Ts as an enzyme source.

DIC, differential interference contrast; rtTA, reverse tetracycline transcriptional activator. See also Figure S2.

2012), bumped UDP-GalNAc analogs **1**, **2**, and **3** were not biosynthesized from their sugar-1-phosphate precursors in the living cell (Figures 4A and 4B).

A lack of AGX1 activity toward synthetic *N*-acetylglucosamine (GlcNAc) analogs has previously prompted the engineering of AGX1 to recognize the corresponding GlcNAc-1-phosphate analog as a substrate (Yu et al., 2012). As WT-AGX1 accepts both GlcNAc-1-phosphate and GalNAc-1-phosphate as substrates, we investigated whether AGX1 mutants could biosynthesize UDP-GalNAc analogs **1**, **2**, and **3** from the corresponding GalNAc-1-phosphate analogs in the living cell. We mutated the gatekeeper residues Phe381 and Phe383 to Gly or Ala in FLAG-tagged AGX1 expression constructs (Yu et al., 2012). GalNAc-1-phosphate analogs were delivered to stably transfected HEK293T cells by virtue of caged precursors **5**, **6**, and **7**. UDP-sugar biosynthesis was then determined by high-performance anion exchange chromatography with pulsed amperometric detection (HPAEC-PAD) of cell lysates. Gly and Ala mutants of Phe383 efficiently biosynthesized bumped UDP-GalNAc analog **1**, while neither Phe381 single mutants nor any Phe381/Phe383 double mutants produced **1** despite equal expression levels (Figure S3A; Yu et al., 2012). In contrast to linear alkyne **1**, neither of the methylated alkynes (**2** or **3**) was biosynthesized by engineered AGX1^{F383A}, hereby called mut-AGX1 (Figures 5B and S3B). We thus concluded that bumped GalNAc-1-P(SATE)$_2$ precursor **5** can be used in conjunction with mut-AGX1 to deliver UDP-GalNAc analog **1** to the living cell and establish a GalNAc-T bump-and-hole system.

In HPAEC-PAD chromatograms of cells biosynthesizing UDP-GalNAc analog **1**, we consistently found a satellite peak that eluted after **1** (arrowhead in Figure 4B). The epimerase UDP-galactose/UDP-GalNAc-4'-epimerase (GALE) maintains a cytosolic equilibrium between UDP-GalNAc and UDP-GlcNAc and has been shown to also accept azide-containing analogs (Boyce et al., 2011; Schulz et al., 2004). We found that GALE epimerizes UDP-GalNAc analog **1** to the corresponding UDP-GlcNAc analog **8** (Figure 4A): first, HPAEC-MS confirmed that the satellite peak was caused by an isomer of UDP-GalNAc analog **1** with 328 m/z $[M-2H]^{2-}$. Second, CRISPR-mediated GALE-KO in the K-562 background abrogated the satellite peak (Figures 4B and S3C–S3E). In contrast, epimerization was still observed in GALE-containing cells. Alkyne-containing UDP-GlcNAc analog **8** might result in the labeling of GlcNAc-containing glycans on the cell surface, such as N-linked glycans. We thus concluded that epimerization of **1** to **8** must be accounted for in glycoproteomics experiments (e.g., by enzymatic abrogation of cell surface N-glycans during sample processing).

A GT Bump-and-Hole System in Living Cells

With a GalNAc-T bump-and-hole system and a method for cellular substrate delivery of **1** in hand, we set out to probe isoenzyme-dependent glycosylation in the living cell. We cloned FLAG-tagged WT- or mut-AGX1 under a constitutive promoter into an expression vector containing VSV-G-tagged WT or BH versions of GalNAc-T1 or T2 under the control of a Dox-inducible promoter (Figure 5A). This setup allowed us to systematically

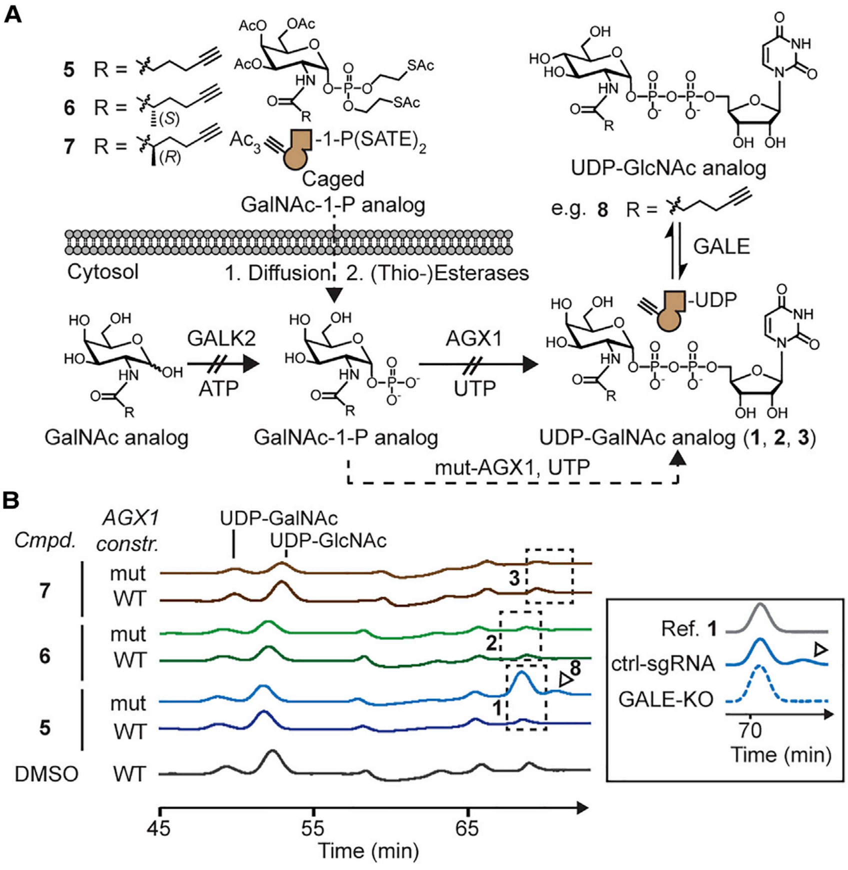

Figure 4. Substrate Delivery to the Cytosol of Living Cells

(A) Schematic of substrate delivery. Non-permissive steps are indicated by crossed arrows. The epimerase GALE interconverts UDP-GlcNAc and UDP-GalNAc.

(B) HPAEC-PAD traces of extracts from HEK293T cells stably expressing WT-AGX1 or mut-AGX1 and fed with the indicated compounds. Dashed boxes indicate retention times of standards in separate reference runs. The product of potential epimerization of **1** by GALE, compound **8**, is marked with an arrowhead. Data are of one experiment and were repeated for compound **5** in HEK293T cells transiently transfected with AGX1 constructs, as well as stably transfected K-562 cells. Insert: epimerization to **8** is suppressed in GALE-deficient K-562 cells expressing mut-AGX1 and fed with **5**, but not cells carrying a control single guide RNA (sgRNA). A reference trace of compound **1** is shown. Data are of one representative out of two independent experiments.

See also Figure S3.

assess any potential background protein labeling when UDP-GalNAc analog **1** could not be biosynthesized or when **1** was biosynthesized but BH-GalNAc-Ts were absent. To preclude any labeling due to epimerization of **1** to UDP-GlcNAc analog **8**, we first used K-562 GALE-KO cells for labeling. Cells were supplemented with GalNAc to compensate for the loss of UDP-GalNAc biosynthesis (Figure S3C). GalNAc-T expression was induced (Figure S4A), cells were fed with caged GalNAc-1-phosphate analog **5**, and the cell surface was reacted with clickable Alexa488-picolyl azide in the presence of a non-membrane-permissive Cu(I) complex (Besanceney-Webler et al., 2011; Uttamapinant et al., 2012). After gating for positive VSV-G signal, flow cytometry showed a more-than-15-fold fluorescence increase when both BH-T2 and substrate **1** were present over controls lacking either component (Figures 5B and S4B). In GALE-containing cells, a more-than-2-fold-higher signal was still measured over control cells when a functional bump-and-hole system was present (Figures S4C and S4D).

In order to better visualize the scope of protein labeling by our bump-and-hole system, we reacted cell surfaces with a clickable version of the infrared dye CF680 to profile labeled cell surface glycoproteins by in-gel fluorescence. We compared protein labeling patterns of GALE-containing K-562 cells stably expressing T1 or T2 constructs. Here, N-linked glycans were removed by PNGase F treatment prior to analysis to reduce background fluorescence (Figure S4E). A profound band pattern was observed when functional bump-and-hole pairs (**5**, mut-AGX1, and BH-GalNAc-Ts) were present (Figures 5C and S4F). Mut-AGX1 was required for labeling, confirming that our experiments probe enzymatic glycosylation rather than non-specific protein modification (Qin et al., 2018). Fluorescence was of similar intensity as in cells treated with well-characterized alkyne-containing *N*-acetylneuraminic acid precursor Ac_4ManNAl (Chang et al., 2009). Furthermore, the presence of BH-GalNAc-T protein was essential, as the omission of Dox induction prevented fluorescent labeling (Figure S4F). Importantly, BH-T1 and BH-T2 produced slightly different band patterns, especially between 25 and 37 kDa. The most intense 110-kDa band migrated at slightly higher molecular weight when glycosylated by BH-T1 instead of BH-T2, indicating that T1 potentially labeled more sites of this particular protein. Digestion with the mucin-selective protease StcE completely removed this band in T2 labeled samples (Figure S4G), confirming labeling of mucin-type proteins that are rich in O-GalNAc glycosylation (Malaker et al., 2019). Discrete band patterns were obtained from in-gel fluorescence experiments when GALE-KO cells were used and a functional bump-and-hole pair was present. In contrast, pulsing the same cells with GlcNAc analog **9**, a precursor of UDP-GlcNAc analog **8**, led to a diffuse background whenever mut-AGX1 was present, but independent of the GalNAc-T construct used (Figure S4H). We re-cloned GalNAc-T and AGX1 constructs into an expression vector with one constitutive promoter for both genes and showed that the labeling pattern after feeding **5** is similar to the pattern using the Dox-inducible constructs (Figure S5A). To profile the identity of labeled cell surface glycoproteins, we synthesized clickable, acid-cleavable biotin-picolyl azide **10** as an enrichment handle of cell surface glycoproteins (Figure 5D). Following lysis and PNGase F treatment, O-

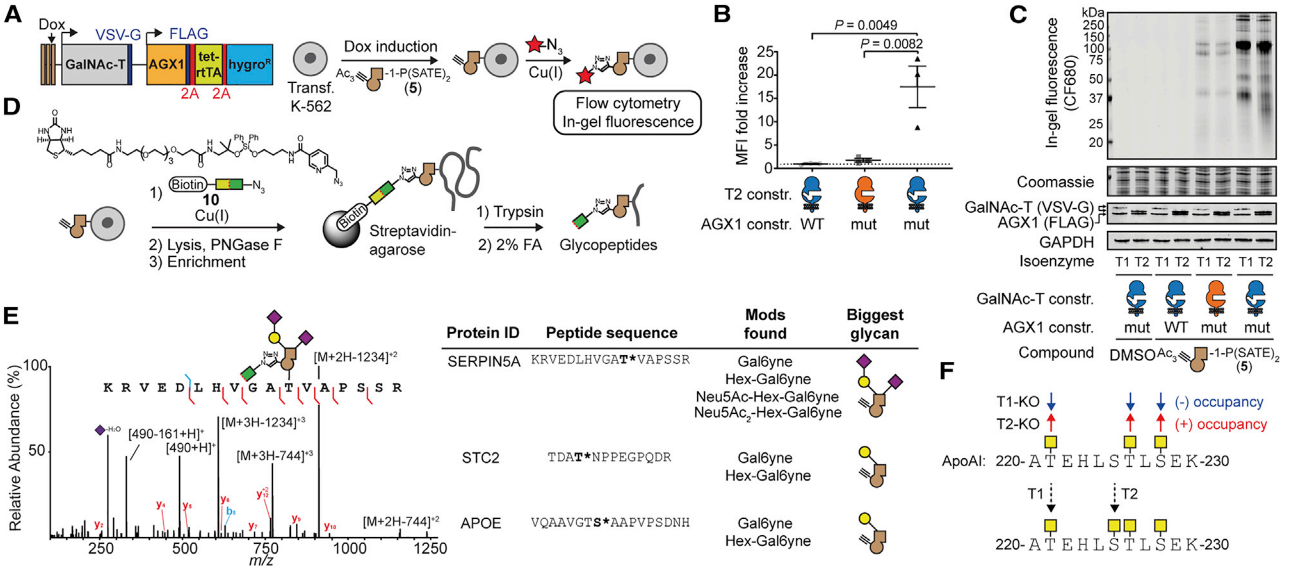

Figure 5. Selective Bioorthogonal Labeling of the Living Cell Surface with Bump-and-Hole Engineered GalNAc-Ts

(A) GalNAc-T and AGX1 co-expression construct and workflow of cell surface labeling. Red star depicts a fluorophore.

(B) Labeling analysis of K-562 GALE-KO cells by flow cytometry of MB488-picolyl azide labeled and intracellular VSV-G-stained cells. Data are represented as individual values from three independent experiments, mean ± SEM of MB488 median fluorescence intensity of VSV-G-positive cells. Statistical analysis was performed by two-tailed ratio paired t test.

(C) Labeling analysis by in-gel fluorescence of PNGase F-treated lysates from metabolically labeled K-562 cells. In-gel fluorescence and Coomassie staining are from one gel, and expression analyses are from one separate western blot. Data are representative of three independent experiments.

(D) Schematic of glycoprotein enrichment and on-bead digest. The bifunctional molecule **10** bears an acid-labile diphenyldisiloxane moiety.

(E) Exemplary MS data: mass spectrum (HCD) of a fully elaborated glycopeptide from SERPIN5A (site Thr39) and further examples from T2-specific sites from STC2 (Thr28) and APOE (Ser308).

(F) Upper panel: previous data on ApoAI$^{220\text{-}230}$ glycosylation in GalNAc-T1 and T2 KO HepG2 cells (Schjoldager et al., 2015); lower panel: glycosylation sites of GalNAc-T1 and T2 uncovered by bump-and-hole engineering.

A, formic acid; MFI, mean fluorescence intensity. See also Figures S4 and S5 and Data S1 and S2.

glycoproteins were enriched and digested on-bead with trypsin to profile the corresponding unmodified peptides by MS. The myelogenous K-562 cell line expresses mucins and mucin-like proteins that are rich in O-GalNAc glycans such as CD36, CD43, CD45, Glycophorins A and C, and MUC18 (https://www.proteinatlas.org/). We found all these proteins enriched (log prob > 5 for BH and < 5 for WT samples, or at least 40 units higher for BH than controls) from lysates of cells carrying functional T1 or T2 bump-and-hole systems (**5**, mut-AGX1, and BH-GalNAc-T) over control cells lacking any component (Figure S5B). It is noteworthy that we found mainly cell surface glycoproteins with abundant O-glycosylation in these experiments, probably because BH-GalNAc-Ts were in constant competition for protein substrates with the corresponding endogenous WT-GalNAc-Ts in these cells.

The use of GalNAc-T-KO SimpleCells has enabled the mapping of glycosylation sites that are introduced by several GalNAc-Ts including T1 and T2, but by design it is not suited to profile the diversity of glycan structures beyond the initiating GalNAc. Our bump-and-hole approach can contribute this information if the bumped GalNAc analog is recognized and extended by downstream GTs. To probe this, we used bump-and-hole pairs to profile the glycoproteome of T1-KO or T2-KO HepG2 cells (Schjoldager et al., 2015). Cells were transfected with GalNAc-T1 or T2 and Dox-induced before labeling with GalNAc-1-phosphate analog **5**. As glycoproteins are abundant in the HepG2 secretome (Schjoldager et al., 2015), we clicked acid-labile enrichment handle **10** onto proteins in conditioned cell culture supernatants. Following on-bead tryptic digest to release non-glycosylated peptides, glycopeptides were liberated by acidic cleavage of the enrichment handle and analyzed by MS. We used the presence of a 491.2238 m/z ion in higher-energy collisional dissociation (HCD) as an indicator for a bumped glycan to trigger peptide sequencing by electron-transfer dissociation (ETD; Figures 5E and S5C–S5E). Following an automated search for glycopeptides containing the GalNAc analog alone or as a part of longer glycans, we obtained raw glycopeptide hits, which we manually validated. Seventy-seven spectra were found of BH-T2-modified glycopeptides that corresponded to 37 peptides with 27 glycosylation sites and the presence of up to 4 different glycan compositions per glycopeptide, the structures of which were inferred based on biosynthetic considerations (Data S1;Schjoldager et al., 2012). In contrast, four glycopepetides were found as modified by BH-T1. Cells fed with DMSO alone gave zero glycopeptide hits. WT-T1- and WT-T2-expressing cells fed with GalNAc-1-phosphate analog **5** showed one and two glycopeptides, respectively. Several known glycosylation sites in the SimpleCell glycoproteomics dataset by Schjoldager et al. (2015) were confirmed herein, and new sites were revealed (Data S1). For

instance, we found hitherto unannotated T2 glycosylation sites in the proteins ITIL2, AMBP, Complement C3 and C4, GPC3 and STC2, and Laminin subunit gamma-1, among others (Figure 5E; Data S1) (Luo et al., 2005). T2 is involved in lipid homeostasis and was found to glycosylate certain apolipoproteins, including ApoE at Thr307 and Ser308, in HepG2 cells (Steentoft et al., 2013; Schjoldager et al., 2015). Both sites were confirmed herein and, additionally, found to be elaborated up to the trisaccharide Neu5Acα-Galβ-GalNAc-Ser/Thr or tetrasaccharide Neu5Acα--Galβ-(Neu5Acα)GalNAc-Ser/Thr structures (Data S1; Schjoldager et al., 2012). Elaboration of GalNAc-T isoenzyme-specific sites cannot be probed with conventional glycoproteomic techniques, highlighting the usefulness of our gain-of-chemical-functionality method to contribute another dimension to the glycoproteome. Our data further resolved ambiguity about the O-glycosylation of apolipoprotein A-1 (ApoA1) in the peptide sequence ATEHLSTLSEK ($ApoA1^{220\text{-}230}$): previous data showed that upon T1-KO, glycosylation of Thr221, Thr226, and Ser228 is slightly decreased, while upon T2-KO, all three sites had increased occupancy (Schjoldager et al., 2015). However, the reasons for these changes in glycosylation were elusive (Schjoldager et al., 2015). Our gain-of-chemical-functionality approach revealed that BH-T2 glycosylated Ser225, while BH-T1 glycosylated Thr221, indicating that T2-KO cells lose Ser225 glycosylation that is compensated by increased glycosylation at the other sites (Figure 5F). We repeated the experiment using BH-GalNAc-Ts and treatment with caged GalNAc-1-phosphate analog **5** only and found similar results (Data S2). Such detailed analysis is facilitated by the chemical tagging strategy that emulates GalNAc-T activity as a result of our bump-and-hole approach.

DISCUSSION

The cellular glycoproteome is subject to disease-related alterations. Bump-and-hole engineering has been artfully employed to obtain insight into the protein substrates of single kinases (Alaimo et al., 2001; Bishop et al., 2000), ADP-ribosyltransferases (Carter-O'Connell et al., 2014; Gibson et al., 2016), and methyltransferases (Wang et al., 2013; Islam et al., 2013; Li et al., 2011), among others (Islam, 2019). Only a few of these systems have been established in the living cell, as cellular delivery of bumped substrates is rarely straightforward, especially when substrates are nucleotide based. Herein, we established the first GT bump-and-hole system in the living cell to profile GalNAc-Ts, the largest GT family in the human genome. The fascinating biology of GalNAc-Ts, with multiple isoenzymes and a profound cross-talk between glycosylation and proteolysis (Revoredo et al., 2016; King et al., 2018), prompted us to develop an isoenzyme-specific chemical reporter. This approach allowed us to expand our knowledge on T1 and T2 glycosylation sites by adding information on the nature of the mature glycans after extension by other GTs.

The era of quantitative biology demands great specificity in tools to probe cellular processes or expressed antigens (Purcell and Godula, 2019). Biological tools, including antibodies, lectins, engineered binding proteins, or silenced hydrolases, have been employed to profile or isolate cellular glycans (Malaker et al., 2019; Chen et al., 2017). While powerful probes, these proteins suffer from various drawbacks, including the dependence of binding on the structural context or the requirement to simplify glycans before performing binding studies. Additionally, all conventional strategies for glycan pull-down or visualization rely on post-glycosylation derivatization or binding. As a consequence, information on GT isoenzyme specificity is already lost or—in the context of KO cell lines—may have been obscured by partial redundancy. This "specificity space" is covered by our bump-and-hole approach that enables GT- and glycosite-specific labeling. As GalNAc-T engineering retains both three-dimensional structure and peptide glycosylation preferences, this strategy probes glycosylation in an unbiased fashion without context dependence. Thereby, we obtained for the first time information on both the site and structure of glycans initiated by a single GT isoenzyme in a single experiment.

Taken together, we now have a strategy in hand to specifically modify GT isoenzyme-dependent glycosylation sites in the living cell.

STAR★METHODS

Detailed methods are provided in the online version of this paper and include the following:

- KEY RESOURCES TABLE
- RESOURCE AVAILABILITY
  - Lead Contact
  - Materials Availability
  - Data and Code Availability
- EXPERIMENTAL MODEL AND SUBJECT DETAILS
- METHOD DETAILS
  - Cloning of full length GalNAc-T1 and T2
  - Cloning, expression and crystallization of BH-T2
  - Screening peptide substrate selectivities of GalNAc-Ts using SAMDI-MS
  - Cloning and site-directed mutagenesis of AGX1
  - Cloning of AGX1 into pSBtet and pSBbi plasmids
  - Generation of K-562 GALE-KO cells
  - Cell transfection
  - GalNAc-T expression analysis and membrane lysate labeling
  - Fluorescence microscopy
  - Analysis of nucleotide-sugar biosynthesis by High Performance Anion Exchange Chromatography
  - Cell surface labeling, flow cytometry and in-gel fluorescence
  - Proteomics
  - Click & enrichment of HepG2 secretome:
  - General organic synthesis
  - Synthesis of bis(*S*-acetyl-2-thioethyl) 3,4,6-tri-*O*-acetyl-2-deoxy-2-(5-hexynoyl)amido-α-D-galactopyranosyl phosphate (5)
  - Synthesis of Bis(*S*-acetyl-2-thioethyl) 3,4,6-tri-*O*-acetyl-2-deoxy-2-(2-(*S*)-methyl-5-hexynoyl)amido-α-D-galactopyranosyl phosphate (6)
  - Synthesis of Bis(*S*-acetyl-2-thioethyl) 3,4,6-tri-*O*-acetyl-2-deoxy-2-(2-(*R*)-methyl-5-hexynoyl)amido-α-D-galactopyranosyl phosphate (7)

- 3-Hydroxypropyl (6-azidomethyl)nicotinate (SI-7)
- 1,3,4,6-Tri-*O*-acetyl-2-deoxy-2-(5-hexynoyl)amido-αβ-D-glucopyranoside (9)
- Biotin-PEG$_4$-dialkoxydiphenylsilane-picolyl azide (10)

- QUANTIFICATION AND STATISTICAL ANALYSIS

SUPPLEMENTAL INFORMATION

Supplemental Information can be found online at https://doi.org/10.1016/j.molcel.2020.03.030.

ACKNOWLEDGMENTS

The authors thank Katrine T. Schjoldager and Hans H. Wandall (both University of Copenhagen, Denmark) for HepG2-T1-KO and HepG2-T2-KO cells and helpful discussions. We thank Lawrence Tabak (National Institutes of Health, Bethesda, MD) for full-length human GalNAc-T2 in the plasmid pCMV-NTAP, Tyler J. Stewart and Matthew Renfrow for advice on GalNAc-T expression, Michael C. Bassik (Stanford University, USA) for the K-562-spCas9 cell line, Jonathan S. Weissman (University of California, San Francisco, USA) for lentiviral plasmids, Jon Agirre (University of York) for help on crystal structure optimization using Privateer, and Ramón Hurtado-Guerrero (University of Zaragoza, Spain) for helpful discussions. We thank David Spiciarich, Yi-Chang Liu, and Christina M. Woo for help with designing experiments. We thank the Cell Services Science Technology Platform at the Francis Crick Institute for support in cell culture. We thank Mia I. Zol-Hanlon for Graphical Abstract image design. The authors are grateful for generous funding by Stanford University, Stanford ChEM-H, University of California, Berkeley, and Howard Hughes Medical Institute. A portion of this work was performed at the Stanford ChEM-H Macromolecular Structure Knowledge Center. This work was supported by the National Institutes of Health (R01 CA200423 to C.R.B. and R21 DK112733 to J.J.K.), the Defense Threat Reduction Agency (GRANT11631647 to M.M.) and the Francis Crick Institute (to B.S. and Z. L.) which receives its core funding from Cancer Research UK (FC001749), the UK Medical Research Council (FC001749), and the Wellcome Trust (FC001749). Use of the Stanford Synchrotron Radiation Lightsource, SLAC National Accelerator Laboratory, is supported by the U.S. Department of Energy, Office of Science, Office of Basic Energy Sciences under Contract No. DE-AC02-76SF00515. The SSRL Structural Molecular Biology Program is supported by the DOE Office of Biological and Environmental Research, and by the National Institutes of Health, National Institute of General Medical Sciences (including P41GM103393). The contents of this publication are solely the responsibility of the authors and do not necessarily represent the official views of NIGMS or NIH. Beamline 5.0.1 of the Advanced Light Source, a U.S. DOE Office of Science User Facility under Contract No. DE-AC02-05CH11231, is supported in part by the ALS-ENABLE program funded by the National Institutes of Health, National Institute of General Medical Sciences, grant P30 GM124169-01. B.S. was supported by a Feodor Lynen Fellowship by the Alexander von Humboldt Foundation. S.P.W. was supported by a Banting Postdoctoral Fellowship from the Canadian Institutes of Health Research. S.A.M. was supported by a National Institute of General Medical Sciences F32 Postdoctoral Fellowship (F32-GM126663-01). M.F.D. was supported by an NWO Rubicon Postdoctoral Fellowship. A.J.A. was supported by a Stanford ChEM-H undergraduate scholarship. J.P. was supported by National Institutes of Health Postdoctoral Fellowship 5F32CA224985. M.A.G. and K.P. were supported by the National Science Foundation Graduate Research Fellowship. M.A.G. was supported by the Stanford ChEM-H Chemistry/Biology Interface Predoctoral Training Program. K.P. was supported by a Stanford Graduate Fellowship.

AUTHOR CONTRIBUTIONS

B.S., M.F.D., S.P.W., S.A.M., L.L., L.J.S.W., J.J.K., M.M., and C.R.B. designed research; B.S., S.A.M., S.P.W., M.F.D., A.J.A., D.M.F., L.J.S.W., Z.L., L.L., D.F., J.P., and M.A.G. ran experiments; B.S., S.A.M., S.P.W., M.F.D., A.J.A., D.M.F., Z.L., L.L., M.M., and C.R.B. analyzed data; J.C., J.P., K.P., and J.J.K. made reagents and cell lines and contributed protocols; B.S. and C.R.B. wrote the paper with input from all authors.

DECLARATION OF INTERESTS

The authors declare no competing interests.

Received: July 16, 2019
Revised: February 18, 2020
Accepted: March 24, 2020
Published: April 22, 2020

REFERENCES

Agirre, J., Iglesias-Fernández, J., Rovira, C., Davies, G.J., Wilson, K.S., and Cowtan, K.D. (2015). Privateer: software for the conformational validation of carbohydrate structures. Nat. Struct. Mol. Biol. *22*, 833–834.

Alaimo, P.J., Shogren-Knaak, M.A., and Shokat, K.M. (2001). Chemical genetic approaches for the elucidation of signaling pathways. Curr. Opin. Chem. Biol. *5*, 360–367.

Antonopoulos, A., Demotte, N., Stroobant, V., Haslam, S.M., van der Bruggen, P., and Dell, A. (2012). Loss of effector function of human cytolytic T lymphocytes is accompanied by major alterations in N- and O-glycosylation. J. Biol. Chem. *287*, 11240–11251.

Bennett, E.P., Mandel, U., Clausen, H., Gerken, T.A., Fritz, T.A., and Tabak, L.A. (2012). Control of mucin-type O-glycosylation: a classification of the polypeptide GalNAc-transferase gene family. Glycobiology *22*, 736–756.

Berrow, N.S., Alderton, D., Sainsbury, S., Nettleship, J., Assenberg, R., Rahman, N., Stuart, D.I., and Owens, R.J. (2007). A versatile ligation-independent cloning method suitable for high-throughput expression screening applications. Nucleic Acids Res. *35*, e45.

Besanceney-Webler, C., Jiang, H., Zheng, T., Feng, L., Soriano del Amo, D., Wang, W., Klivansky, L.M., Marlow, F.L., Liu, Y., and Wu, P. (2011). Increasing the efficacy of bioorthogonal click reactions for bioconjugation: a comparative study. Angew. Chem. Int. Engl. *50*, 8051–8056.

Bishop, A.C., Ubersax, J.A., Petsch, D.T., Matheos, D.P., Gray, N.S., Blethrow, J., Shimizu, E., Tsien, J.Z., Schultz, P.G., Rose, M.D., et al. (2000). A chemical switch for inhibitor-sensitive alleles of any protein kinase. Nature *407*, 395–401.

Boyce, M., Carrico, I.S., Ganguli, A.S., Yu, S.-H., Hangauer, M.J., Hubbard, S.C., Kohler, J.J., and Bertozzi, C.R. (2011). Metabolic cross-talk allows labeling of O-linked β-N-acetylglucosamine-modified proteins via the N-acetylgalactosamine salvage pathway. Proc. Natl. Acad. Sci. USA *108*, 3141–3146.

Carter-O'Connell, I., Jin, H., Morgan, R.K., David, L.L., and Cohen, M.S. (2014). Engineering the substrate specificity of ADP-ribosyltransferases for identifying direct protein targets. J. Am. Chem. Soc. *136*, 5201–5204.

Chang, P.V., Chen, X., Smyrniotis, C., Xenakis, A., Hu, T., Bertozzi, C.R., and Wu, P. (2009). Metabolic labeling of sialic acids in living animals with alkynyl sugars. Angew. Chem. Int. Engl. *48*, 4030–4033.

Chen, V.B., Arendall, W.B., 3rd, Headd, J.J., Keedy, D.A., Immormino, R.M., Kapral, G.J., Murray, L.W., Richardson, J.S., and Richardson, D.C. (2010). MolProbity: all-atom structure validation for macromolecular crystallography. Acta Crystallogr. D Biol. Crystallogr. *66*, 12–21.

Chen, M., Shi, X., Duke, R.M., Ruse, C.I., Dai, N., Taron, C.H., and Samuelson, J.C. (2017). An engineered high affinity Fbs1 carbohydrate binding protein for selective capture of N-glycans and N-glycopeptides. Nat. Commun. *8*, 15487.

Choi, J., Wagner, L.J.S., Timmermans, S.B.P.E., Malaker, S.A., Schumann, B., Gray, M.A., Debets, M.F., Takashima, M., Gehring, J., and Bertozzi, C.R. (2019). Engineering Orthogonal Polypeptide GalNAc-Transferase and UDP-Sugar Pairs. J. Am. Chem. Soc. *141*, 13442–13453.

de Las Rivas, M., Lira-Navarrete, E., Gerken, T.A., and Hurtado-Guerrero, R. (2019). Polypeptide GalNAc-Ts: from redundancy to specificity. Curr. Opin. Struct. Biol. *56*, 87–96.

Dull, T., Zufferey, R., Kelly, M., Mandel, R.J., Nguyen, M., Trono, D., and Naldini, L. (1998). A third-generation lentivirus vector with a conditional packaging system. J. Virol. *72*, 8463–8471.

Emsley, P., Lohkamp, B., Scott, W.G., and Cowtan, K. (2010). Features and development of Coot. Acta Crystallogr. Sect. D Biol. Crystallogr. *66*, 486–501.

Evans, P. (2006). Scaling and assessment of data quality. Acta Crystallogr. D Biol. Crystallogr. *62*, 72–82.

Fritz, T.A., Raman, J., and Tabak, L.A. (2006). Dynamic association between the catalytic and lectin domains of human UDP-GalNAc:polypeptide α-N-acetylgalactosaminyltransferase-2. J. Biol. Chem. *281*, 8613–8619.

Gaziel-Sovran, A., Segura, M.F., Di Micco, R., Collins, M.K., Hanniford, D., Vega-Saenz de Miera, E., Rakus, J.F., Dankert, J.F., Shang, S., Kerbel, R.S., et al. (2011). miR-30b/30d regulation of GalNAc transferases enhances invasion and immunosuppression during metastasis. Cancer Cell *20*, 104–118.

Gerken, T.A., Raman, J., Fritz, T.A., and Jamison, O. (2006). Identification of common and unique peptide substrate preferences for the UDP-GalNAc:polypeptide α-N-acetylgalactosaminyltransferases T1 and T2 derived from oriented random peptide substrates. J. Biol. Chem. *281*, 32403–32416.

Gerken, T.A., Jamison, O., Perrine, C.L., Collette, J.C., Moinova, H., Ravi, L., Markowitz, S.D., Shen, W., Patel, H., and Tabak, L.A. (2011). Emerging paradigms for the initiation of mucin-type protein O-glycosylation by the polypeptide GalNAc transferase family of glycosyltransferases. J. Biol. Chem. *286*, 14493–14507.

Gibson, B.A., Zhang, Y., Jiang, H., Hussey, K.M., Shrimp, J.H., Lin, H., Schwede, F., Yu, Y., and Kraus, W.L. (2016). Chemical genetic discovery of PARP targets reveals a role for PARP-1 in transcription elongation. Science *353*, 45–50.

Gilbert, L.A., Larson, M.H., Morsut, L., Liu, Z., Brar, G.A., Torres, S.E., Stern-Ginossar, N., Brandman, O., Whitehead, E.H., Doudna, J.A., et al. (2013). CRISPR-mediated modular RNA-guided regulation of transcription in eukaryotes. Cell *154*, 442–451.

Hang, H.C., and Bertozzi, C.R. (2005). The chemistry and biology of mucin-type O-linked glycosylation. Bioorg. Med. Chem. *13*, 5021–5034.

Hang, H.C., Yu, C., Kato, D.L., and Bertozzi, C.R. (2003). A metabolic labeling approach toward proteomic analysis of mucin-type O-linked glycosylation. Proc. Natl. Acad. Sci. USA *100*, 14846–14851.

Hintze, J., Ye, Z., Narimatsu, Y., Madsen, T.D., Joshi, H.J., Goth, C.K., Linstedt, A., Bachert, C., Mandel, U., Bennett, E.P., et al. (2018). Probing the contribution of individual polypeptide GalNAc-transferase isoforms to the *O*-glycoproteome by inducible expression in isogenic cell lines. J. Biol. Chem. *293*, 19064–19077.

Islam, K. (2018). The Bump-and-Hole Tactic: Expanding the Scope of Chemical Genetics. Cell Chem. Biol. *25*, 1171–1184.

Islam, K., Zheng, W., Yu, H., Deng, H., and Luo, M. (2011). Expanding cofactor repertoire of protein lysine methyltransferase for substrate labeling. ACS Chem. Biol. *6*, 679–684.

Islam, K., Chen, Y., Wu, H., Bothwell, I.R., Blum, G.J., Zeng, H., Dong, A., Zheng, W., Min, J., Deng, H., and Luo, M. (2013). Defining efficient enzyme-cofactor pairs for bioorthogonal profiling of protein methylation. Proc. Natl. Acad. Sci. USA *110*, 16778–16783.

Ji, S., Samara, N.L., Revoredo, L., Zhang, L., Tran, D.T., Muirhead, K., Tabak, L.A., and Ten Hagen, K.G. (2018). A molecular switch orchestrates enzyme specificity and secretory granule morphology. Nat. Commun. *9*, 3508.

Kabsch, W. (2010). XDS. Acta Crystallogr. Sect. D Biol. Crystallogr. *66*, 125–132.

Kightlinger, W., Lin, L., Rosztoczy, M., Li, W., DeLisa, M.P., Mrksich, M., and Jewett, M.C. (2018). Design of glycosylation sites by rapid synthesis and analysis of glycosyltransferases. Nat. Chem. Biol. *14*, 627–635.

King, S.L., Goth, C.K., Eckhard, U., Joshi, H.J., Haue, A.D., Vakhrushev, S.Y., Schjoldager, K.T., Overall, C.M., and Wandall, H.H. (2018). TAILS N-terminomics and proteomics reveal complex regulation of proteolytic cleavage by *O*-glycosylation. J. Biol. Chem. *293*, 7629–7644.

Kowarz, E., Löscher, D., and Marschalek, R. (2015). Optimized Sleeping Beauty transposons rapidly generate stable transgenic cell lines. Biotechnol. J. *10*, 647–653.

Li, J., Wei, H., and Zhou, M.M. (2011). Structure-guided design of a methyl donor cofactor that controls a viral histone H3 lysine 27 methyltransferase activity. J. Med. Chem. *54*, 7734–7738.

Lira-Navarrete, E., Iglesias-Fernández, J., Zandberg, W.F., Compañón, I., Kong, Y., Corzana, F., Pinto, B.M., Clausen, H., Peregrina, J.M., Vocadlo, D.J., et al. (2014). Substrate-guided front-face reaction revealed by combined structural snapshots and metadynamics for the polypeptide N-acetylgalactosaminyltransferase 2. Angew. Chem. Int. Engl. *53*, 8206–8210.

Lira-Navarrete, E., de Las Rivas, M., Compañón, I., Pallarés, M.C., Kong, Y., Iglesias-Fernández, J., Bernardes, G.J.L., Peregrina, J.M., Rovira, C., Bernadó, P., et al. (2015). Dynamic interplay between catalytic and lectin domains of GalNAc-transferases modulates protein O-glycosylation. Nat. Commun. *6*, 6937–6948.

Luo, C.W., Pisarska, M.D., and Hsueh, A.J.W. (2005). Identification of a stanniocalcin paralog, stanniocalcin-2, in fish and the paracrine actions of stanniocalcin-2 in the mammalian ovary. Endocrinology *146*, 469–476.

Malaker, S.A., Pedram, K., Ferracane, M.J., Bensing, B.A., Krishnan, V., Pett, C., Yu, J., Woods, E.C., Kramer, J.R., Westerlind, U., et al. (2019). The mucin-selective protease StcE enables molecular and functional analysis of human cancer-associated mucins. Proc. Natl. Acad. Sci. USA *116*, 7278–7287.

Mátés, L., Chuah, M.K.L., Belay, E., Jerchow, B., Manoj, N., Acosta-Sanchez, A., Grzela, D.P., Schmitt, A., Becker, K., Matrai, J., et al. (2009). Molecular evolution of a novel hyperactive Sleeping Beauty transposase enables robust stable gene transfer in vertebrates. Nat. Genet. *41*, 753–761.

McCoy, A.J., Grosse-Kunstleve, R.W., Adams, P.D., Winn, M.D., Storoni, L.C., and Read, R.J. (2007). Phaser crystallographic software. J. Appl. Crystallogr. *40*, 658–674.

Murshudov, G.N., Vagin, A.A., and Dodson, E.J. (1997). Refinement of macromolecular structures by the maximum-likelihood method. Acta Crystallogr. D Biol. Crystallogr. *53*, 240–255.

Narimatsu, Y., Joshi, H.J., Schjoldager, K.T., Hintze, J., Halim, A., Steentoft, C., Nason, R., Mandel, U., Bennett, E.P., Clausen, H., and Vakhrushev, S.Y. (2019). Exploring Regulation of Protein O-Glycosylation in Isogenic Human HEK293 Cells by Differential O-Glycoproteomics. Mol. Cell. Proteomics *18*, 1396–1409.

Peng, R.Q., Wan, H.Y., Li, H.F., Liu, M., Li, X., and Tang, H. (2012). MicroRNA-214 suppresses growth and invasiveness of cervical cancer cells by targeting UDP-N-acetyl-α-D-galactosamine:polypeptide N-acetylgalactosaminyltransferase 7. J. Biol. Chem. *287*, 14301–14309.

Perez-Riverol, Y., Csordas, A., Bai, J., Bernal-Llinares, M., Hewapathirana, S., Kundu, D.J., Inuganti, A., Griss, J., Mayer, G., Eisenacher, M., et al. (2019). The PRIDE database and related tools and resources in 2019: improving support for quantification data. Nucleic Acids Res. *47*, D442–D450.

Pouilly, S., Bourgeaux, V., Piller, F., and Piller, V. (2012). Evaluation of analogues of GalNAc as substrates for enzymes of the mammalian GalNAc salvage pathway. ACS Chem. Biol. *7*, 753–760.

Prescher, J.A., Dube, D.H., and Bertozzi, C.R. (2004). Chemical remodelling of cell surfaces in living animals. Nature *430*, 873–877.

Purcell, S.C., and Godula, K. (2019). Synthetic glycoscapes: addressing the structural and functional complexity of the glycocalyx. Interface Focus *9*, 20180080.

Qin, W., Qin, K., Fan, X., Peng, L., Hong, W., Zhu, Y., Lv, P., Du, Y., Huang, R., Han, M., et al. (2018). Artificial Cysteine S-Glycosylation Induced by Per-O-Acetylated Unnatural Monosaccharides during Metabolic Glycan Labeling. Angew. Chem. Int. Engl. *57*, 1817–1820.

Radhakrishnan, P., Dabelsteen, S., Madsen, F.B., Francavilla, C., Kopp, K.L., Steentoft, C., Vakhrushev, S.Y., Olsen, J.V., Hansen, L., Bennett, E.P., et al. (2014). Immature truncated O-glycophenotype of cancer directly induces oncogenic features. Proc. Natl. Acad. Sci. USA *111*, E4066–E4075.

Revoredo, L., Wang, S., Bennett, E.P., Clausen, H., Moremen, K.W., Jarvis, D.L., Ten Hagen, K.G., Tabak, L.A., and Gerken, T.A. (2016). Mucin-type O-glycosylation is controlled by short- and long-range glycopeptide substrate recognition that varies among members of the polypeptide GalNAc transferase family. Glycobiology *26*, 360–376.

Schjoldager, K.T.-B.G., Vakhrushev, S.Y., Kong, Y., Steentoft, C., Nudelman, A.S., Pedersen, N.B., Wandall, H.H., Mandel, U., Bennett, E.P., Levery, S.B., and Clausen, H. (2012). Probing isoform-specific functions of polypeptide GalNAc-transferases using zinc finger nuclease glycoengineered SimpleCells. Proc. Natl. Acad. Sci. USA *109*, 9893–9898.

Schjoldager, K.T., Joshi, H.J., Kong, Y., Goth, C.K., King, S.L., Wandall, H.H., Bennett, E.P., Vakhrushev, S.Y., and Clausen, H. (2015). Deconstruction of O-glycosylation–GalNAc-T isoforms direct distinct subsets of the O-glycoproteome. EMBO Rep. *16*, 1713–1722.

Schulz, J.M., Watson, A.L., Sanders, R., Ross, K.L., Thoden, J.B., Holden, H.M., and Fridovich-Keil, J.L. (2004). Determinants of function and substrate specificity in human UDP-galactose 4′-epimerase. J. Biol. Chem. *279*, 32796–32803.

Sletten, E.M., and Bertozzi, C.R. (2009). Bioorthogonal chemistry: fishing for selectivity in a sea of functionality. Angew. Chem. Int. Engl. *48*, 6974–6998.

Steentoft, C., Vakhrushev, S.Y., Vester-Christensen, M.B., Schjoldager, K.T.B.G., Kong, Y., Bennett, E.P., Mandel, U., Wandall, H., Levery, S.B., and Clausen, H. (2011). Mining the O-glycoproteome using zinc-finger nuclease-glycoengineered SimpleCell lines. Nat. Methods *8*, 977–982.

Steentoft, C., Vakhrushev, S.Y., Joshi, H.J., Kong, Y., Vester-Christensen, M.B., Schjoldager, K.T.B.G., Lavrsen, K., Dabelsteen, S., Pedersen, N.B., Marcos-Silva, L., et al. (2013). Precision mapping of the human O-GalNAc glycoproteome through SimpleCell technology. EMBO J. *32*, 1478–1488.

Ten Hagen, K.G., Fritz, T.A., and Tabak, L.A. (2003). All in the family: the UDP-GalNAc:polypeptide N-acetylgalactosaminyltransferases. Glycobiology *13*, 1R–16R.

Termini, J.M., Silver, Z.A., Connor, B., Antonopoulos, A., Haslam, S.M., Dell, A., and Desrosiers, R.C. (2017). HEK293T cell lines defective for O-linked glycosylation. PLoS ONE *12*, e0179949.

Uttamapinant, C., Tangpeerachaikul, A., Grecian, S., Clarke, S., Singh, U., Slade, P., Gee, K.R., and Ting, A.Y. (2012). Fast, cell-compatible click chemistry with copper-chelating azides for biomolecular labeling. Angew. Chem. Int. Engl. *51*, 5852–5856.

Varki, A. (2017). Biological roles of glycans. Glycobiology *27*, 3–49.

Varki, A., Cummings, R.D., Esko, J.D., Stanley, P., Hart, G.W., Aebi, M., Darvill, A.G., Kinoshita, T., Packer, N.H., Prestegard, J.H., et al. (2017). Essentials of glycobiology, Third Edition (Cold Spring Harbor Laboratory Press).

Wang, R., Islam, K., Liu, Y., Zheng, W., Tang, H., Lailler, N., Blum, G., Deng, H., and Luo, M. (2013). Profiling genome-wide chromatin methylation with engineered posttranslation apparatus within living cells. J. Am. Chem. Soc. *135*, 1048–1056.

Winn, M.D., Ballard, C.C., Cowtan, K.D., Dodson, E.J., Emsley, P., Evans, P.R., Keegan, R.M., Krissinel, E.B., Leslie, A.G.W., McCoy, A., et al. (2011). Overview of the CCP4 suite and current developments. Acta Crystallogr. Sect. D Biol. Crystallogr. *67*, 235–242.

Woo, C.M., Iavarone, A.T., Spiciarich, D.R., Palaniappan, K.K., and Bertozzi, C.R. (2015). Isotope-targeted glycoproteomics (IsoTaG): a mass-independent platform for intact N- and O-glycopeptide discovery and analysis. Nat. Methods *12*, 561–567.

Yu, S.-H., Boyce, M., Wands, A.M., Bond, M.R., Bertozzi, C.R., and Kohler, J.J. (2012). Metabolic labeling enables selective photocrosslinking of O-GlcNAc-modified proteins to their binding partners. Proc. Natl. Acad. Sci. USA *109*, 4834–4839.

Zaro, B.W., Yang, Y.-Y., Hang, H.C., and Pratt, M.R. (2011). Chemical reporters for fluorescent detection and identification of O-GlcNAc-modified proteins reveal glycosylation of the ubiquitin ligase NEDD4-1. Proc. Natl. Acad. Sci. USA *108*, 8146–8151.

CellPress

STAR★METHODS

KEY RESOURCES TABLE

REAGENT or RESOURCE	SOURCE	IDENTIFIER
Antibodies		
Mouse anti-VSV-G P4D5	Abcam (Cambridge, UK)	Abcam Cat# ab50549; RRID:AB_883494
Mouse anti-FLAG M2	Sigma Aldrich (St. Louis, USA)	Sigma-Aldrich Cat# P2983; RRID:AB_439685
Rabbit anti-GAPDH	Abcam	Abcam Cat# ab128915; RRID:AB_11143050
Rabbit anti-GAPDH-HRP	Abcam	Abcam Cat# ab185059
Rabbit anti-giantin	Abcam	Abcam Cat# ab37266; RRID:AB_880195
Mouse anti-GALE	Santa Cruz (Dallas, USA)	Satna Cruz Cat# sc-390407
Donkey anti-mouse IgG Alexa Fluor® 568	Abcam	Abcam Cat# ab175472; RRID:AB_2636996
Donkey anti-rabbit IgG Alexa Fluor® 647	Abcam	Abcam Cat# ab150075; RRID:AB_2752244
Donkey anti-mouse IgG Alexa Fluor® 647	Jackson ImmunoResearch (Cambridgeshire, UK)	Jackson ImmunoResearch Labs Cat# 715-605-151; RRID:AB_2340863
IRDye® 800CW Donkey anti-mouse IgG	LI-COR Biosciences (Lincoln, USA)	LI-COR Biosciences Cat# 925-32212; RRID:AB_2716622
IRDye® 680RD Donkey anti-rabbit IgG	LI-COR Biosciences	LI-COR Biosciences Cat# 925-68073; RRID:AB_2716687
Chemicals, Peptides, and Recombinant Proteins		
CF680-picolyl azide	Biotium (Fremont, USA)	Cat. No. 96003
MB488 picolyl azide	Click Chemistry Tools (Scottsdale, USA)	Cat. No. 1208
BTTAA	Jena Bioscience (Jena, Germany)	CAS No. 1334179-85-9
Ac_4ManNAl	Chang et al., 2009	CAS No. 935658-93-8
EA2 peptide PTTDSTTPAPTTK	AnaSpec (Fremont, USA)	Cat. No. AS-63841
biotin-DADPS-picolyl azide	This paper; subsequently custom synthesized from Sussex Research Laboratories (Ottawa, CA)	N/A
Biotin-$(PEG)_4$-azide	Thermo Fisher (Waltham, USA)	Cat. No. B10184
3,4,6-tri-O-acetyl-2-deoxy-2-(5-hexynoyl) amido-α-D-galactopyranoside (cmpd. **SI-1**)	Choi et al., 2019	N/A
Bis(S-acetyl-2-thioethyl)-*N*-diisopropylamino phosophoamidite **SI-2**	Yu et al., 2012	N/A
3,4,6-tri-O-acetyl-2-deoxy-2-(2-(*S*)-methyl-5-hexynoyl)amido-α-D-galactopyranoside (cmpd. **SI-3**)	Choi et al., 2019	N/A
3,4,6-tri-O-acetyl-2-deoxy-2-(2-(*R*)-methyl-5-hexynoyl)amido -α-D-galactopyranoside (cmpd. **SI-4**)	Choi et al., 2019	N/A
Methyl 6-(azidomethyl)nicotinate (**SI-5**, Methods S1)	Oakwood Chemical (Estill, USA)	Cat. No. 375798; CAS No. 384831-56-5
Biotin alcohol **SI-8**	Woo et al., 2015	N/A
Uridine 5′-diphospho-2-deoxy-2-(5-hexynoyl)amido-α-D-galactopyranoside disodium salt (cmpd. **1**)	Choi et al., 2019	N/A
Uridine 5′-diphospho-2-deoxy-2-(2-(*S*)-methyl-5-hexynoyl)amido-α-D-galactopyranoside disodium salt (cmpd. **2**)	Choi et al., 2019	N/A

(*Continued on next page*)

Continued

REAGENT or RESOURCE	SOURCE	IDENTIFIER
Uridine 5′-diphospho-2-deoxy-2-(2-(*R*)-methyl-5-hexynoyl)amido-α-D-galactopyranoside disodium salt (cmpd. **3**)	Choi et al., 2019	N/A
Bis(S-acetyl-2-thioethyl) 3,4,6-tri-O-acetyl-2-deoxy-2-(5-hexynoyl)amido-α-D-galactopyranosyl phosphate (cmpd. **5**)	This paper	N/A
Bis(S-acetyl-2-thioethyl) 3,4,6-tri-O-acetyl-2-deoxy-2-(2-(*S*)-methyl-5-hexynoyl) amido-α-D-galactopyranosyl phosphate (cmpd. **6**)	This paper	N/A
Bis(S-acetyl-2-thioethyl) 3,4,6-tri-O-acetyl-2-deoxy-2-(2-(*R*)-methyl-5-hexynoyl) amido-α-D-galactopyranosyl phosphate (cmpd. **7**)	This paper	N/A
1,3,4,6-Tri-O-acetyl-2-deoxy-2-(5-hexynoyl)amido-αβ-D-glucopyranoside (cmpd. **9**)	This paper	N/A
Biotin-PEG_4-dialkoxydiphenylsilane-picolyl azide (cmpd. **10**)	This paper	N/A
Biotinylated *Vicia villosa* lectin	Vector Labs (Burlingame, USA)	Vector Laboratories Cat# B-1235; RRID:AB_2336855
AlexaFluor488 conjugated streptavidin	Thermo Fisher	Cat. No. S11223
IRDye 800CW Streptavidin	LI-COR Biosciences, Lincoln, USA)	Cat. No. 926-32230
Soluble FLAG-tagged WT-GalNAc-T1	Choi et al., 2019	N/A
Soluble FLAG-tagged BH-GalNAc-T1	Choi et al., 2019	N/A
Soluble FLAG-tagged WT-GalNAc-T2	Choi et al., 2019	N/A
Soluble FLAG-tagged BH-GalNAc-T2	Choi et al., 2019	N/A
Soluble His_6-tagged BH-T2 protein	This paper	N/A
Critical Commercial Assays		
Subcellular fractionation kit for cultured cells	Thermo Fisher	Cat. No. 78840
Deposited Data		
Glycoproteomics raw data	This paper	PRIDE accession ID: PXD018048
Crystal structure BH-T2/EA2/UDP/Mn2+	This paper	PDB: 6E7I
Crystal structure BH-T2/UDP-GalNAc analog/Mn2+	This paper	PDB: 6NQT
Proteomics raw data	This paper	PRIDE accession ID: PXD017989
Gel and blot full images	This paper	https://doi.org/10.17632/nh4vww6hxj.2
Fluorescence microscopy imaging data	This paper	https://doi.org/10.17632/nh4vww6hxj.2
Experimental Models: Cell Lines		
K-562	Laboratory of Jonathan Weissman, UCSF	N/A
K-562 pSBtet-WT-hAGX1	This paper	N/A
K-562 pSBtet-mut-hAGX1	This paper	N/A
K-562 pSBtet-WT-hAGX1-BH-T1	This paper	N/A
K-562 pSBtet-mut-hAGX1-WT-T1	This paper	N/A
K-562 pSBtet-mut-hAGX1-BH-T1	This paper	N/A
K-562 pSBtet-WT-hAGX1-BH-T2	This paper	N/A
K-562 pSBtet-mut-hAGX1-WT-T2	This paper	N/A
K-562 pSBtet-mut-hAGX1-BH-T2	This paper	N/A
K-562 pSBbi-WT-hAGX1-BH-T1	This paper	N/A

(*Continued on next page*)

Continued

REAGENT or RESOURCE	SOURCE	IDENTIFIER
K-562 pSBbi-mut-hAGX1-WT-T1	This paper	N/A
K-562 pSBbi-mut-hAGX1-BH-T1	This paper	N/A
K-562 pSBbi-WT-hAGX1-BH-T2	This paper	N/A
K-562 pSBbi-mut-hAGX1-WT-T2	This paper	N/A
K-562 pSBbi-mut-hAGX1-BH-T2	This paper	N/A
K-562-spCas9	Laboratory of Jonathan Weissman, UCSF	N/A
K-562-spCas9 ctrl-sgRNA	This paper	N/A
K-562-spCas9 ctrl-sgRNA pSBtet-WT-hAGX1	This paper	N/A
K-562-spCas9 ctrl-sgRNA pSBtet-mut-hAGX1	This paper	N/A
K-562-spCas9 ctrl-sgRNA pSBtet-WT-hAGX1-BH-T2	This paper	N/A
K-562-spCas9 ctrl-sgRNA pSBtet-mut-hAGX1-WT-T2	This paper	N/A
K-562-spCas9 ctrl-sgRNA pSBtet-mut-hAGX1-BH-T2	This paper	N/A
K-562-spCas9 GALE-KO	This paper	N/A
K-562-spCas9 GALE-KO pSBtet-WT-hAGX1	This paper	N/A
K-562-spCas9 GALE-KO pSBtet-mut-hAGX1	This paper	N/A
K-562-spCas9 GALE-KO pSBtet-WT-hAGX1-BH-T2	This paper	N/A
K-562-spCas9 GALE-KO pSBtet-mut-hAGX1-WT-T2	This paper	N/A
K-562-spCas9 GALE-KO pSBtet-mut-hAGX1-BH-T2	This paper	N/A
HepG2	ATCC	ATCC Cat# HB-8065; RRID:CVCL_0027
HepG2 pSBtet-GH	This paper	N/A
HepG2 pSBtet-GH-WT-T1	This paper	N/A
HepG2 pSBtet-GH-BH-T1	This paper	N/A
HepG2 pSBtet-GH-WT-T2	This paper	N/A
HepG2 pSBtet-GH-BH-T2	This paper	N/A
HepG2-T1-KO	Schjoldager et al., 2015	N/A
HepG2-T1-KO pSBtet-mut-hAGX1-WT-T1	This paper	N/A
HepG2-T1-KO pSBtet-mut-hAGX1-BH-T1	This paper	N/A
HepG2-T2-KO	Schjoldager et al., 2015	N/A
HepG2-T2-KO pSBtet-mut-hAGX1-WT-T1	This paper	N/A
HepG2-T2-KO pSBtet-mut-hAGX1-BH-T1	This paper	N/A
HEK293T	ATCC	ATCC Cat# CRL-3216; RRID:CVCL_0063
HEK293T pIRES-puro-WT-hAGX1	This paper	N/A
HEK293T pIRES-puro-AGX1	This paper	N/A
Oligonucleotides		
GALE_sgRNA1 CCGGGATTACATCCATGTCG	Termini et al., 2017	N/A
GALE_sgRNA2 TCAGCTCCTGGACCCGCCGC	Termini et al., 2017	N/A
GAL4 control sgRNA GAACGACTAGTTAGGCGTGTA	Gilbert et al., 2013	N/A

(*Continued on next page*)

Continued

REAGENT or RESOURCE	SOURCE	IDENTIFIER
Primer T2_F1 CTTGCGGCCGCGATGCGGCG GCGCTCGCGGATGC	This paper	N/A
Primer T2_F2 GCAGAGCTCGTTTAGTGAACCGT CAGAATTGATCTAC	This paper	N/A
Primer T2_F3 AAAGGCCTCTGAGGCCACCATGC GGCGGCGCGCTCG	This paper	N/A
Primer T2_F4 GCGTAGCTGAAACCGGCAAAGTA CGGTGGCCAGACTTTAACCAG	This paper	N/A
Primer T2_R1 GTCATCGTCTTTGTAGT CCTGCTGCAGGTTGAGCGTGAAC TTCCACTGC	This paper	N/A
Primer T2_R2 GATGAATTCCTACTTGTC GTCATCGTCTTTGTAGTCCTGCTG CAGGTTGAGCG	This paper	N/A
Primer T2_R3 TCTCTCGGATCCCTGCTGCAGGT TGAGGGTGAACTTCC	This paper	N/A
Primer T2_R4 TTTGGCCTGACAGGCCCTACTT ACCCAGGCGGTTCATTTCGATA TCAGTGTACTGCTGCAGGTTGA GCGGTG	This paper	N/A
Primer T2_R5 GTGATGGTGATGTTTCTGCTGCAG GTTGAGCGTGAA	This paper	N/A
T1 and T2 site-directed mutagenesis primers	Choi et al., 2019	N/A
GalNAc-T1 gBlock: NCBI GenBank® accession number NM_020474 with overhangs AAATTTGCGGCCGCAGTGCCATGA (5′) and AAATATTCGGATCCGGGCCC (3′)	This paper	N/A
Primer T1_F1 AAATTTGCGGCCGCAGTGCC	This paper	N/A
Primer T1_F2 AAAGGCCTCTGAGGCCACCATGAG AAAATTTGCATACTGCAAG	This paper	N/A
Primer T1_R1 GGGCCCGGATCCGAATATTTCTGG	This paper	N/A
Primer T1_R2 TTTGGCCTGACAGGCCCTACTTACC CAGGCGGTTCATTTCGATATCAGT GTAGAATATTTCTGGCAGG GTGACGTTTC	This paper	N/A
AGX1 mutagenesis primer 383G_F CAAACCCAATGGAATAAA GATGGAAAAAGGTGTC TTTGACATCTTCCAG	This paper	N/A

(*Continued on next page*)

Continued

REAGENT or RESOURCE	SOURCE	IDENTIFIER
AGX1 mutagenesis primer 383G_R CAAAGACACCTTTTTCC ATCTTTATTCCATTGGG TTTGTCTGGCTTAATTAAC	This paper	N/A
AGX1 mutagenesis primer 381G/383G_F CAAACCCAATGGAATAAAGATGGAAA AAGGTGTCGGTGACATCTTCCAG	This paper	N/A
AGX1 mutagenesis primer 381G/383G_R CACCGACACCTTTTTCCATCTTTATTCC ATTGGGTTTGTCTGGCTTAATTAAC	This paper	N/A
AGX1 mutagenesis primer 381A_F GGAAAAAGCTGTCTTTGACAT CTTCCAGTTTGC	This paper	N/A
AGX1 mutagenesis primer 381A_R GATGTCAAAGACAGCTTTTTCCATCT TTATTCCATTGGG	This paper	N/A
AGX1 mutagenesis primer 383A_F GAATAAAGATGGAAAAATTTGTCG CTGACATCTTCCAGTTTGC	This paper	N/A
AGX1 mutagenesis primer 383A_R GATGTCAGCGACAAATTTTTCCATCT TTATTCCATTGGGTTTG	This paper	N/A
AGX1 mutagenesis primer 381A/383A_F GATGGAAAAAGCTGTCGCTGACATCT TCCAGTTTGC	This paper	N/A
AGX1 mutagenesis primer 381A/383A_R GATGTCAGCGACAG CTTTTTCCATCTTTATTCCATTGGG	This paper	N/A
AGX1 mutagenesis primer WT_F CCCAATGGAATAAAGATGGAAAAA TTTGTCTTTGACATCTTCC	This paper	N/A
AGX1 mutagenesis primer WT_R GACAAATTTTTCCATCTTTATTCCATTG GGTTTGTCTGGC	This paper	N/A
AGX1 subcloning_F ATGAACATTA ATGACCTCAAACTCACGTTGTCC	This paper	N/A
AGX1 subcloning_R CTTGTCATCGTCTTTGTAGTCAAT ACCATTTTTC	This paper	N/A
AGX1_gBlock_5′ GGCCCGCCTTCCC TGGGGAATCTCTGCGCACGCGCAGAA CGCTTCGACCAATGAAAACACAGGAAG CCGTCCGCGCAACCGCGTTGCGTCACT TCTGCCGCCCCTGTTTCAAGGTATATAG CCGTAGACGGAACTTCGCCTTTCTCTC GGCCTTAGCGCCATTTTTTTGGGTGAG TGTTTTTTGGTTCCTGCGTTGGGATTCC GTGTACAATCCATAGACATCTGACCTC GGCACTTAGCATCATCACAGCAAACTA ACTGTAGCCTTTCTCTCTTTCCCTGTAG AAACCTCTGCACCTGAGGCCACCATGA ACATTAATGACCTCAAACTCACGTTGT CCAAAGCTGGGC	This paper	N/A

(*Continued on next page*)

Continued

REAGENT or RESOURCE	SOURCE	IDENTIFIER
AGX1_gBlock_3′ CATGAGCTGGTGAA AAATGGTATTGACTACAAAGACGATG ACGACAAGGGCAGTGGAGCTACTAA CTTCAGCCTGCTGAAGCAGGCTGGT GACGTCGAGGAGAATCCTGGCCCC ATGTCTAGACTGGACAAGAGCAAAG TCATAAACGGC	This paper	N/A
AGX1 extension_F GGCCCGCCTTCCCTGG	This paper	N/A
AGX1 extension_R GCTCTTGTCCAGTCTAGACATGGGGC	This paper	N/A
FLAG introduction primer_F GACTACAA AGACGATGACGACAAGTGAGCGGCCG CATAGATAACTGATCC	This paper	N/A
FLAG introduction primer_R CTTGTCGTCATCGTCT TTGTAGTCAATACCATTTTT CACCAGCTCATGAACTCCATTC	This paper	N/A
Recombinant DNA		
pU6-sgGAL4-4	Gilbert et al., 2013	RRID:Addgene_46916
pCMV(CAT)T7-SB100	addgene	RRID:Addgene_34879
pSBtet-GH	addgene	RRID:Addgene_60498
pSBtet-WT-hAGX1	This paper	N/A
pSBtet-mut-hAGX1	This paper	N/A
pSBtet-WT-hAGX1-BH-T1	This paper	N/A
pSBtet-mut-hAGX1-WT-T1	This paper	N/A
pSBtet-mut-hAGX1-BH-T1	This paper	N/A
pSBtet-WT-hAGX1-BH-T2	This paper	N/A
pSBtet-mut-hAGX1-WT-T2	This paper	N/A
pSBtet-mut-hAGX1-BH-T2	This paper	N/A
pSBbi-GH	addgene	RRID:Addgene_60514
pSBbi-WT-hAGX1-BH-T1	This paper	N/A
pSBbi-mut-hAGX1-WT-T1	This paper	N/A
pSBbi-mut-hAGX1-BH-T1	This paper	N/A
pSBbi-WT-hAGX1-BH-T2	This paper	N/A
pSBbi-mut-hAGX1-WT-T2	This paper	N/A
pSBbi-mut-hAGX1-BH-T2	This paper	N/A
pIRES-puro-hAGX1^{F383G}	Yu et al., 2012	N/A
pIRES-puro-mut-hAGX1	This paper	N/A
pIRES-puro-WT-hAGX1	This paper	N/A
pIRES-puro-hAGX1^{F381G}	This paper	N/A
pIRES-puro-hAGX1^{F381A}	This paper	N/A
pIRES-puro-hAGX1$^{F381G/F383G}$	This paper	N/A
pIRES-puro-hAGX1$^{F381/AF383A}$	This paper	N/A
pOPING-trunc-BH-T2	This paper	N/A
Software and Algorithms		
XDS	Kabsch, 2010	http://xds.mpimf-heidelberg.mpg.de/
CCP4 software package	Winn et al., 2011	https://www.ccp4.ac.uk/
SCALA	Evans, 2006	Part of CCP4
Phaser	McCoy et al., 2007	Part of CCP4

(*Continued on next page*)

Continued		
REAGENT or RESOURCE	SOURCE	IDENTIFIER
Coot	Emsley et al., 2010	https://www2.mrc-lmb.cam.ac.uk/personal/pemsley/coot/
REFMAC5	Murshudov et al., 1997	Part of CCP4
MOLPROBITY	Chen et al., 2010	http://molprobity.manchester.ac.uk/
Privateer	Agirre et al., 2015	Part of CCP4

RESOURCE AVAILABILITY

Lead Contact

Further information and requests for resources and reagents should be directed to and will be fulfilled by the Lead Contact, Carolyn R. Bertozzi (bertozzi@stanford.edu).

Materials Availability

HepG2-T1-KO, HepG2-T2-KO cells and all cell lines derived from these are subject to an MTA with the University of Copenhagen, and Katrine T. Schjoldager (schjoldager@sund.ku.dk) or Hans H. Wandall (hhw@sund.ku.dk) should be contacted. All other cell lines generated in this study are available through the Lead Author. All plasmids generated in this study are available through the Lead Author; of these, plasmids from addgene are protected by an UBMTA and can be obtained for non-commercial use under an equal UBMTA. Newly synthesized chemicals will be shared while stocks last, and synthetic procedures are in the Supporting Information.

Data and Code Availability

Crystal structures are available in the protein databank (PDB: 6E7I and PDB: 6NQT). The mass spectrometry proteomics and glycoproteomics data have been deposited to the ProteomeXchange Consortium via the PRIDE (Perez-Riverol et al., 2019) partner repository with the dataset identifiers PRIDE: PXD017989 and PRIDE: PXD018048.

EXPERIMENTAL MODEL AND SUBJECT DETAILS

All cell lines were mycoplasma-free, as confirmed using the MycoAlert Kit (Lonza, Basel, Switzerland). Female K-562 cells with stable expression of *Streptococcus pyogenes* Cas9 (K-562-spCas9) were a gift from Michael Bassik (Stanford University, USA) and propagated in RPMI (Thermo Fisher) with 10% (v/v) FBS, penicillin (100 U/mL) and streptomycin (100 μg/mL) at 37 °C with 5% CO_2. Expression of spCas9 was confirmed by western blot. K-562 GALE-KO cells were derived from K-562-spCas9 cells, and were grown under the same conditions, but with addition of 200 μM GalNAc and 20 μM galactose to the culture medium. GALE-KO cells were maintained in standard growth medium supplemented with 20 μM galactose and 200 μM GalNAc. GALE was not detectable by western blot using mouse anti-GALE sc-390407 (Santa Cruz Biotechnology, Dallas, USA) over at least 20 passages. Cell authentication was performed via STR profiling and species authentication. Female K-562 cells were a gift from Jonathan Weissman (University of California, San Francisco). Cells were grown in RPMI with 10% (v/v) FBS, penicillin (100 U/mL) and streptomycin (100 μg/mL). Cell authentication was performed via STR profiling and species authentication. HEK293T (ATCC CRL-3216) of unknown sex were from ATCC and grown in DMEM (Thermo Fisher) with 10% (v/v) FBS (Thermo Fisher), penicillin (100 U/mL) and streptomycin (100 μg/mL, GE Healthcare, Chicago, USA). Cell authentication was performed via STR profiling and species authentication. Freestyle™ 293-F cells of unknown sex were propagated in FreeStyle™ 293 Expression Medium (Thermo Fisher) with 10% (v/v) fetal bovine serum (FBS, Thermo Fisher) at 37 °C and 8% CO_2 with orbital rotation at 135 rpm. Cells were not authenticated. HepG2 cells (ATCC HB-8065), HepG2-T1-KO and HepG2-T2-KO cells (a gift from Katrine T. Schjoldager and Hans H. Wandall, University of Copenhagen, Denmark) were propagated in low-glucose DMEM (Caisson Labs, Smithfield, USA) with 10% (v/v) FBS, penicillin (100 U/mL) and streptomycin (100 μg/mL). Cells were not authenticated.

METHOD DETAILS

The following nomenclature of GalNAc-T and AGX1 genes will be used herein: WT-T1, WT-T2: wild-type constructs; BH-T1: I238A/L295A; BH-T2: I253A/L310A; mut-AGX1: F383A.

Cloning of full length GalNAc-T1 and T2

Primers and gBlocks were from Integrated DNA Technologies (IDT, Coralville, USA) and Elim Biopharm (Hayward, USA). Restriction enzymes were from New England Biolabs except for PasI (Thermo Fisher, Waltham, USA). PfuUltra II (Agilent, Santa Clara, USA) and Advantage Genomic LA polymerase (Takara, Kusatsu, Japan) were used for T1 and T2, respectively. T4 DNA ligase (New England Biolabs, Ipswitch, USA) and LONG DNA ligase (Takara) were used for ligation of T1 and T2, respectively. Q5 high-fidelity DNA

polymerase (New England Biolabs) was used for overlap extension PCR. All plasmids were sequenced before use at Elim Biopharmaceuticals, Inc. (Hayward, USA).

The plasmids pSBtet-GH and pSBbi-GH were a gift from Eric Kowarz (Addgene plasmid #60498; http://addgene.org/60498; RRID:Addgene_60498, and Addgene plasmid # 60514; http://addgene.org/60514; RRID:Addgene_60514) (Kowarz et al., 2015).

Full length human GalNAc-T2 (EBI accession number LC043140.1) in pCMV-NTAP was a kind gift from Lawrence Tabak (National Institutes of Health, Bethesda, MD) (Choi et al., 2019). T2 was first sub-cloned with a C-terminal FLAG tag into a version of pFLAG-CMV-2 (Sigma Aldrich, St. Louis, USA) in which the N-terminal FLAG tag had been removed, using a two-step PCR elongation procedure with the primer T2_F1, T2_R1 (step 1) and T2_R2 (step 2), and a NotI/EcoRI restriction strategy. Site-directed mutagenesis (I253A/L310A) was performed at this step according to a published procedure (Choi et al., 2019). T2 constructs were cloned into pFLAG-CMV-5.1 (Sigma Aldrich) using the primers T2_F2 and T2-R3 and a NotI/BamHI strategy. These constructs were used as templates to clone T2 constructs into pSBtet-GH (Kowarz et al., 2015) with a C-terminal VSV-G tag, using the primers T2_F3 and T2_R4 with an SfiI cloning strategy.

Full length GalNAc-T1 (NCBI GenBank® accession number NM_020474) was used as a gBlock (IDT) with overhangs and NotI/BamHI restriction sites, and amplified with the primers T1_F1 and T1_R1 prior to cloning into pFLAG-CMV-5.1 using a NotI/BamHI strategy. Site-directed mutagenesis (I238A/L295A) was performed at this step according to a published procedure (Choi et al., 2019). These constructs were used as templates to clone T1 constructs into pSBtet-GH with a C-terminal VSV-G tag, using the primers T1_R1 and T1_R2 with an SfiI cloning strategy.

Cloning, expression and crystallization of BH-T2

The plasmid pOPING was a gift from Ray Owens (Addgene plasmid # 26046; http://addgene.org/26046; RRID:Addgene_26046) (Berrow et al., 2007). A secretion construct of BH-T2 (aa 75-571), chosen according to literature precedent of crystallization of the WT enzyme (Lira-Navarrete et al., 2014), with a His_6 tag in pOPING was used for protein expression and crystallization. The coding sequence in pFLAG-Myc-CMV-19 (Choi et al., 2019) was cloned into KpnI/PmeI-digested pOPING using the primers T2_F4 and T2_R5, with In-Fusion HD Cloning Kit (Takara). Freestyle^{TM} 293-F cells were transfected with pOPING-trunc-BH-T2 using 293fectin (Thermo Fisher) according to the manufacturer's specifications, with $5x10^7$ to $1x10^8$ cells and 30 μg plasmid DNA. After 24 h, cells were harvested (5 min, 500 g, room temperature) and medium was renewed (30 mL). Cells were harvested and conditioned supernatant collected after one, three, and five days, and medium was renewed each time. Conditioned supernatant was passed through a freshly-packed column containing 2 mL HisPur NiNTA resin slurry (Thermo Fisher) pre-conditioned with water and Wash Buffer (20 mM imidazole, 50 mM Tris-HCl, 125 mM NaCl, pH 7.5). The resin was treated with Wash Buffer (2x15 mL), and subsequently with Elution Buffer (12.5 mL, 200 mM imidazole, 50 mM Tris-HCl, 125 mM NaCl, pH 7.5, cOmplete protease inhibitors (Roche, Basel, Switzerland)). Eluted protein was dialyzed using Amicon Ultra-15 centrifuge filters (10 kDa MWCO, Millipore) against Crystallization Buffer (25 mM Tris-HCl, 0.5 mM EDTA, 1 mM TCEP, pH 8.0). Protein was stable for one week at 4 °C. For long-term storage at −80 °C, 80% (v/v) aq. glycerol was added to a final concentration of 25% (v/v). Protein was re-buffered to Crystallization Buffer immediately prior to use. Typically, 1.2 mg protein was purified from one 30 mL sample of conditioned supernatant.

Crystals were grown using sitting drop vapor diffusion at room temperature. Drops were set-up using a Douglas Oryx8 Nanodrop Dispensing Robot (Douglas Instruments Ltd, Berkshire, United Kingdom).

Crystals of BH-T2 complexed with UDP, Mn^{2+} and EA2 peptide were obtained through co-crystallization using 0.15 μL of 5 mM $MnCl_2$, 5 mM UDP, 6 mM EA2, and 10 mg/mL protein in Crystallization Buffer and 0.15 μL precipitant solution (11%–32% (v/v) PEG8000, 0.1 mM HEPES, pH 6.5-8.5) against 80 μL of precipitant solution. Crystals were grown for 1 week before being cryoprotected in 16% ethylene glycol and frozen in liquid nitrogen before diffraction.

Crystals of BH-T2 complexed with **1** and Mn^{2+} were obtained through soaking of EA2 and **1** into co-crystals of BH-T2 with UDP and Mn^{2+}. EA2 peptide electron density was too weak to properly discern in crystal structures. Initial drops were created using 0.15 μL of 5 mM $MnCl_2$, 5 mM UDP, 10 mg/mL T2 BH protein in 25 mM Tris-HCl (pH 8.0), 0.5 mM EDTA, and 1 mM TCEP and 0.15 μL precipitant solution (11%–23% PEG8000, 0.1 mM HEPES, pH 7.5-8.5) against 80 μL of precipitant solution. Crystals were grown for 18 days before soaking and diffraction. Crystals were soaked in a solution with EA2 by adding 0.45 μL of a 50 mM EA2 solution in water to the original drop for 30 minutes to 2 hours. Crystals were harvested and soaking was performed with a solution of 20 mM **1** with 16% ethylene glycol, 11%–23% PEG8000, 0.1 mM HEPES, 5 mM $MnCl_2$, pH 7.5-8.5 for 1-2 hours.

Single crystal X-ray diffraction of BH-T2/EA2/UDP/Mn^{2+} (PDB 6E7I) was performed at 95K using ALS Beamline 5.0.1 (Lawrence Berkeley National Labs, Berkeley, USA) and single wavelength of 0.97741 Å, with a Dectris Pilatus3 S 6M Detector (Dectris Ltd., Baden-Daettwil, Switzerland).

Single crystal X-ray diffraction of BH-T2/**1**/Mn^{2+} (PDB 6NQT) was performed at 100K using Stanford Synchrotron Radiation Lightsource Beamline 7-1 (SLAC National Accelerator Laboratory, Menlo Park, CA, USA) and single wavelength of 0.9753 Å, with an ADSC Quantum 315r CCD Detector (Quantum Detectors Ltd, Harwell Oxford, United Kingdom).

Data was processed using XDS (Kabsch, 2010), and scaled with SCALA (Evans, 2006) and other programs implemented with the CCP4 software (Winn et al., 2011). Crystal structures were determined using molecular replacement with Phaser (McCoy et al., 2007), using published structures (PDB: 2FFU for PDB: 67EI and PDB: 4D0T for PDB: 6NQT) as the templates (Fritz et al., 2006; Lira-Navarrete et al., 2014). The initial model was improved through multiple cycles of manual modeling in Coot (Emsley et al., 2010), and refinement using REFMAC5 (Murshudov et al., 1997). The final structural model was validated using MOLPROBITY (Chen et al.,

2010). UDP-GalNAc analog conformation was validated using Privateer (Agirre et al., 2015). Images were prepared using Pymol 2.0.0 (Schrödinger LLC, New York, USA). Electron density was rendered at 1 σ and carved at 1.6 Å. Structure statistics are given in Table S1.

Screening peptide substrate selectivities of GalNAc-Ts using SAMDI-MS

SAMDI-MS screenings were performed similarly to previously reported (Kightlinger et al., 2018). Briefly, 50 μM solutions of 361 peptides of the general formula $AX_{-1}TX_{+1}APRC$, where X_{-1} and X_{+1} are 19 natural amino acids except for Cys, were screened individually with 25 nM purified soluble FLAG-tagged WT-GalNAc-T1 or 50 nM purified soluble FLAG-tagged WT-GalNAc-T2 and 0.25 mM UDP-GalNAc in SAMDI buffer (50 mM Tris-HCl pH 7.4, 50 mM NaCl, 5 mM $MnCl_2$), at 37 °C for 3 h (Choi et al., 2019). For BH-GalNAc-Ts, the same peptide array was screened with 30 nM purified soluble FLAG-tagged BH-GalNAc-T1 and 0.25 mM compound **1** in SAMDI buffer at 37 °C for 21 h, or with 175 nM soluble FLAG-tagged BH-GalNAc-T2 and 0.25 mM compound **1** in SAMDI buffer at 37 °C for 3 h (Choi et al., 2019). After the reaction of each 10 μL volume, 5 mM EDTA were added for quenching and 2.5 μL TCEP reducing gel (Thermo Fisher) were added for reduction incubation for 1 h at 37 °C. Reduced mixture (2 μL) was added to the gold islands of a 384 SAMDI plate, prepared as described before (Kightlinger et al., 2018). The SAMDI plate was incubated at room temperature for 30 min, and washed with water, ethanol and water, nitrogen blow dried, and treated with 10 mg/mL 2′,4’,6’-trihydroxyacetophenone monohydrate (Sigma Aldrich) matrix in acetonitrile. The SAMDI plates were analyzed with a Sciex MALDI-TOF/TOF 5800 (Applied Biosystems, Foster City, USA), equipped with the software Explorer 4.1.0 to analyze data. Percent intensity of glycosylated peptides related to the sum of unreacted and glycosylated peptide was recorded. Preferred X_{-1} amino acids, combined with all X_{+1} amino acids, were chosen to repeat the experiment for BH-GalNAc-Ts.

Cloning and site-directed mutagenesis of AGX1

$AGX1^{F383G}$ in pIRES-puro3 was used as a template to generate AGX1 constructs (Yu et al., 2012). Site-directed mutagenesis was performed using the following primers (positions 381 and 383 underlined): 383G_F and 383G_R for F381G; 381G/383G_F and 381G/383G_R for F381G/F383G. A C-terminal FLAG-tag was then introduced into these constructs using a site-directed mutagenesis strategy with FLAG introduction primer_F and _R. These constructs were then used to produce other AGX1 constructs. Primer pairs (positions 381 and 383 underlined) were: 381A_F/_R and for F381A; F383A_F/_R and for F383A; 381A/383GA_F/_R and for F381A/F383A; WT_F/_R for WT.

Cloning of AGX1 into pSBtet and pSBbi plasmids

WT-AGX1 or mut-AGX1 were cloned into the backbone of pSBtet-GH containing WT-T1, BH-T1, WT-T2, or BH-T2 by replacing the eGFP coding sequence. C-terminally FLAG-tagged AGX1 sequences were amplified from the corresponding pIRES-puro3 constructs (see above) using the primers AGX1 subcloning_F/_R, purified by gel extraction, and subjected to an overlap extension PCR with equimolar amounts (10 fmol each) of the gBlocks (IDT) AGX1_gBlock_5′ and AGX1_gBlock_3′ using 10 mM dNTPs and Q5 polymerase. Briefly, a 50 μL reaction was run for 15 cycles using the following protocol: 95 °C (2 min), 95 °C (30 s), 64 °C (30 s), 72 °C (30 s – return to step 2), 72 °C (5 min), then treated with the primers (500 nmol) AGX1 extension_F and AGX1 extension_R and continued for another 18 cycles with an annealing temperature of 55 °C. The resulting PCR product was used to replace the eGFP cloning sequence of pSBtet-GH containing T1 or T2 using an XcmI/PasI strategy to digest the vector and In-Fusion HD Cloning Kit to insert AGX1 sequences. AGX1-containing pSBtet vectors are named pSBtet-WT-hAGX1 or pSBtet-mut-hAGX1. For constitutive expression of GalNAc-Ts, eGFP coding sequence was accordingly replaced with AGX1 in pSBbi-GH by GeneArt (Thermo).

Vectors herein are pSBtet-GH (eGFP, Luciferase), pSBtet-WT-hAGX (WT-AGX1, Luciferase), pSBtet-mut-hAGX1 (mut-AGX1, Luciferase), pSBtet-GH-WT-T1 (eGFP, WT-T1), pSBtet-GH-BH-T1 (eGFP, BH-T1), pSBtet-GH-WT-T2 (eGFP, WT-T2), pSBtet-GH-BH-T2 (eGFP, BH-T2), pSBtet-mut-hAGX1-WT-T1 (mut-AGX1, WT-T1), pSBtet-WT-hAGX1-BH-T1 (WT-AGX1, BH-T1), pSBtet-mut-hAGX1-BH-T1 (mut-AGX1, BH-T1), pSBtet-mut-hAGX1-WT-T2 (mut-AGX1, WT-T2), pSBtet-WT-hAGX1-BH-T2 (WT-AGX1, BH-T2), pSBtet-mut-hAGX1-BH-T2 (mut-AGX1, BH-T2), pSBbi-WT-hAGX1-BH-T1 (WT-AGX1, BH-T1), pSBbi-mut-hAGX1-WT-T1 (mut-AGX1, WT-T1), pSBbi-mut-hAGX1-BH-T1 (mut-AGX1, BH-T1), pSBbi-WT-hAGX1-BH-T2 (WT-AGX1, BH-T2), pSBbi-mut-hAGX1-WT-T2 (mut-AGX1, WT-T2), pSBbi-mut-hAGX1-BH-T2 (mut-AGX1, BH-T2).

Generation of K-562 GALE-KO cells

Single-guide (sg) RNAs GALE_sgRNA1 and GALE_sgRNA2 targeting human *GALE* were chosen according to Termini et al., 2017. Specificity and minimal off-target effects of sgRNAs were further confirmed *in silico* (www.synthego.com). SgRNAs were cloned into the vector pU6-sgGAL4-4 (Gilbert et al., 2013), a gift from Jonathan Weissman (University of California, San Francisco), using a BstXI-BlpI restriction strategy and annealed oligodeoxynucleotides with the following overhangs as inserts: TTG-[forward sgRNA]-GTTTAAGAGC and TTAGCTCTTAAAC-[reverse complementary sgRNA]-CAACAAG.

HEK293T cells were seeded at 1.2 million cells per well of a 6-well plate. After 24 h, cells were transfected with 1.5 μg sgRNA-coding vector and a third generation lentiviral packaging mix (0.1 μg GAG/POL, 0.1 μg REV, and 0.2 μg VSV-G (Dull et al., 1998), using TransIT-293 (Mirus Bio LLC, Madison, USA). After 16 h, supernatant was aspirated and replaced with fresh growth medium. After another 30 h, supernatant was collected, and filtered by 0.45 μm syringe filtration. Virus-containing supernatant was stored at −80 °C.

For lentiviral infection, 200,000 K-562-spCas9 cells were seeded in 1 mL medium in a 24-well plate. After 24 h, 300 μL virus-containing supernatant and 8 μL polybrene (Sigma Aldrich) were added. After another 24 h, cells were harvested and treated with fresh growth medium. After another 24 h, cells were harvested and treated with growth medium containing 1.25 μg/mL puromycin. Selection was carried out for 96 h, and cells were expanded in puromycin-free growth medium. The knockout cells were fed 100 uM of GalNAc for 2 days before FACS sorting. After 48 h, the cells were stained with 5 μg/mL biotinylated *Vicia villosa* lectin (Vector Labs, Burlingame, USA) and 4 μg/mL of AlexaFluor488 conjugated streptavidin (Thermo Fisher) on ice for 30 min. The cells were washed twice with PBS + 0.5% BSA and viable cells were sorted based on AlexaFluor488 fluorescence intensity and low SYTOX Red (Thermo) staining according to a published procedure (Gaziel-Sovran et al., 2011). UDP-GalNAc was absent in these cells unless GalNAc was supplemented (Figure S3C). Both sgRNAs had the same effect, and only sgRNA_GALE2 was used for further experiments.

Cell transfection

Tet-system approved FBS (Takara) was used to propagate all cell lines transfected with pSBtet-GH-based plasmids. pCMV(CAT)T7-SB100 was a gift from Zsuzsanna Izsvak (Addgene plasmid # 34879; http://addgene.org/34879; RRID:Addgene_34879) (Mátés et al., 2009).

HEK293T cells were transfected with pIRES-puro3 plasmids containing AGX1 constructs using TransIT-293 (Mirus Bio LLC, Madison, USA) according to the manufacturer's instructions and 37.5 μg DNA per 15 cm dish or 15 μg DNA per 10 cm dish. After 24 h, medium was aspirated, and cells were either treated with fresh growth medium and compounds for analysis of nucleotide-sugar biosynthesis (see below), or growth medium containing 5 μg/mL puromycin (Sigma Aldrich) for selection of stable cells for two weeks.

K-562 cells were transfected with pSBtet-based plasmids using Lipofectamine LTX (Thermo Fisher) according to the manufacturer's specifications, with 200000 cells in 1 mL growth medium, 1.25 μg pSBtet and 62.5 ng pCMV(CAT)T7-SB100 plasmid DNA. After 24 h, cells were harvested and selected in growth medium containing 150 μg/mL hygromycin B (Thermo Fisher) for 7-10 days to obtain stable cells.

HepG2, HepG2-T1-KO and HepG2-T2-KO cells were transfected with Lipofectamine 3000 (Thermo Fisher) according to the manufacturer's instructions, e.g., using 2.5 μg pSBtet and 125 ng pCMV(CAT)T7-SB100 plasmid DNA per well of a 6-well plate. After 24 h, medium was aspirated, and cells were treated with fresh growth medium containing 600 μg/mL (HepG2) or 500 μg/mL (HepG2-T1-KO and HepG2-T2-KO) hygromycin B for two weeks to obtain stable cells. Following selection, cells were propagated in 200 μg/mL hygromycin B in growth medium.

GalNAc-T expression analysis and membrane lysate labeling

HepG2 cells stably transfected with pSBtet-GH, pSBtet-GH-WT-T1, pSBtet-GH-BH-T1, pSBtet-GH-WT-T2, or pSBtet-GH-BH-T2 were seeded into 6-well plates at 40% confluency. After 24 h, the supernatant was aspirated and changed to fresh growth medium without hygromycin B, containing the indicated concentration of doxycycline (AppliChem, Maryland Heights, USA). After 24 h, the treatment was repeated in fresh growth medium. After another 24 h, cells were washed with PBS (1 mL), treated with ice-cold PBS containing 1 mM EDTA (1 mL) and incubated for 10 min at 4 °C. Cells were transferred to a 1.5 mL reaction tube, harvested (600 g, 5 min, 4 °C), and resuspended in ice-cold 50 mM Tris-HCl pH 8, 5 mM EDTA, 150 mM NaCl, 1% (v/v) NP-40, 0.5% (v/v) sodium deoxycholate, 0.1% (w/v) SDS (0.2 mL) containing cOmplete protease inhibitors (Roche, Basel, Switzerland). Cells were briefly vortexed, incubated for 30 min at 4 °C with agitation, and centrifuged (18000 g, 30 min, 4 °C). The supernatant was transferred to a new tube, and the protein concentration was measured by BCA (Thermo Fisher). SDS-PAGE and western blot were performed using 15 μg protein per lane, and blots were decorated with mouse anti-VSV-G-tag P5D4 (abcam, Cambridge, UK) and rabbit anti-GAPDH-HRP conjugate ab185059 (abcam).

Membrane protein glycosylation was performed on a membrane fraction from untransfected or stable HepG2 cells. Cells were seeded in a 15 cm dish, grown to 50% confluency and treated with doxycycline (5 μg/mL final concentration). After 24 h, doxycycline treatment was repeated. After another 24 h, cells were washed with PBS (10 mL) and scraped in PBS (5 mL). Cells were harvested by centrifugation (300 g, 5 min, 4 °C), washed once with ice-cold PBS (2 mL) and frozen at −80 °C. Cells were thawed on ice and fractionated using the Subcellular Fractionation Kit for Cultured Cells (Thermo Fisher) according to the manufacturer's instructions, with 0.4 mL of both Cytoplasmic Extraction Buffer and Membrane Extraction Buffer. Protein concentration was measured by BCA (Thermo Fisher), and fractions were frozen after addition of 80% (v/v) glycerol (10% final concentration).

In vitro glycosylation reactions were performed using 10 μg membrane protein of stably transfected HepG2 cell lines in 20 μL reaction volume containing 62.5 mM Tris-HCl pH 7.4, 150 mM NaCl, 10 mM $MnCl_2$, 250 μM UDP-GalNAc analog and 500 μM UDP-GalNAc at 37 °C for 12 h. Reactions were heat-inactivated at 95 °C for 20 s and cooled to 4 °C. Then, alkyne-containing reaction mixtures were sequentially treated with equal volumes (1.25 μL each) of 2 mM biotin-PEG_4-azide (Thermo Fisher), 2 mM BTTAA (Click Chemistry Tools, Scottsdale, USA), 20 mM $CuSO_4$ and 100 mM sodium ascorbate (final concentrations 100 μM biotin probe, 100 μM BTTAA, 1 mM $CuSO_4$ and 5 mM sodium ascorbate). Click reactions were carried out at room temperature for 2 h and quenched by addition of 50 mM EDTA. Reaction mixtures were then subjected to SDS-PAGE and blotted on nitrocellulose membranes. The total protein amount was assessed using the REVERT protein staining kit (LI-COR Biosciences, Lincoln, USA), and biotinylation was detected using IRDye 800CW Streptavidin (LI-COR Biosciences) according to the manufacturer's instructions.

In vitro glycosylations were replicated using a membrane protein fraction from untransfected HepG2 cells that was depleted for internal GalNAc-T activity by three heat (90 °C)-cool (4 °C) cycles of 30 s each. Soluble FLAG-tagged WT- or BH-GalNAc-Ts (*8*) were added at a final concentration of 20 nM (T1) or 10 nM (T2), along with bumped UDP-GalNAc analog (250 μM) and UDP-GalNAc (500 μM). Glycosylation, click reaction and Streptavidin detection were performed as described above.

Fluorescence microscopy

Fluorescence microscopy was performed with stably transfected HepG2 cells. Cells were Dox-induced (T1-transfected cells: 2 μg/mL; T2-transfected cells: 0.2 μg/mL) for 24 h, then trypsinated and transferred in growth medium to a 24 well plate containing circular 12 mm coverslips (Electron Microscopy Services, Hatfield, USA) pre-treated with human fibronectin (5 μg/mL in PBS, Merck & Co., Kenilworth, USA) for 20 min and washed with PBS. Cells were treated with Dox again and incubated for another 24 h. Supernatant was aspirated, cells were washed in PBS with 100 mg/L Ca^{2+} and 100 mg/mL Mg^{2+} (DPBS), and fixed with 4% (v/v) paraformaldehyde (Sigma Aldrich) in PBS for 10 min in the dark. Cells were washed with 50 mM ammonium chloride in DPBS and DPBS with 2% (w/v) BSA (Incubation Buffer). Cells were then permeabilized with 0.5% (v/v) Tween-20 in incubation buffer for 10 min in the dark, and subsequently treated with antibody solutions in the same buffer: mouse anti-VSV-G-tag, (1:200), and rabbit anti-Giantin (1:500, 9B6, abcam). Cells were incubated for 1 h, washed twice with Incubation Buffer with 0.1% Tween-20, and incubated with secondary antibodies in Incubation Buffer containing 0.5% (v/v) Tween-20: Donkey anti-mouse Alexa Fluor 568 (1:200, abcam) and donkey anti-rabbit Alexa Fluor 647 (1:200, abcam). Cells were incubated for 1 h in the dark, washed twice with Incubation Buffer with 0.1% (v/v) tween-20, and mounted on glass slides using ProLong Diamond Antifade mounting solution (Thermo Fisher). Cells were imaged using a Nikon A1R+ Resonant Scanning Confocal Microscope.

Analysis of nucleotide-sugar biosynthesis by High Performance Anion Exchange Chromatography

Cells expressing AGX1-FLAG (transient or stably transfected HEK293T in 20 mL growth medium in a 15 cm dish or 5 million stable K-562 in 4 mL growth medium) were treated with caged GalNAc-1-phosphate analogs (100 μM final concentration from 10 mM or 100 mM stock solutions in DMSO; all samples of an experiment contained equal amounts of DMSO) or DMSO vehicle. After 7 h, K-562 cells were harvested (500 g, 5 min, 4 °C) and the supernatant was aspirated. HEK293T cells were washed once on the plate with cold PBS (8 mL), scraped in cold 1 mM EDTA in PBS (8 mL), transferred to a conical tube and harvested (300 g, 5 min, 4 °C). Cell pellets were resuspended in PBS (1 mL), 0.8 mL of that suspension was transferred to O-ring tubes (1.5 mL, Thermo Fisher) and harvested. Zirconia/silica beads (0.1 mm, BioSpec, Bertlesville, USA) were added at a similar volume as the cell pellet, followed by 1:1 acetonitrile/water (1 mL). Cells were lysed with a bead beater (FastPrep-24, MP Biomedicals, Santa Ana, USA) for 30 s at 6 m/s, and the mixture was left at 4 °C for 10 min. Samples were centrifuged (14000 g, 10 min, 4 °C), and the resulting supernatant was transferred to a new 1.5 mL tube. The solvent was removed by speed vac. The residue was resuspended in a solution of 15 μM ADP-α-D-glucose (Sigma Aldrich) in LCMS-grade water (Thermo Fisher, 0.2-0.4 mL). The solution was membrane-filtered (30 min, 14000 g) using a 3 kDa cut-off filter (Centricon, Merck) and analyzed by high performance anion exchange chromatography. The residual cell suspension in PBS (0.2 mL) was harvested, and the pellet was resuspended in M-PER lysis buffer (Thermo Fisher) containing cOmplete protease inhibitor (0.2 mL). The solution was incubated for 10 min at room temperature and centrifuged (14000 g, 10 min, 4 °C). The protein concentration of the supernatant was measured by BCA, and samples were used for analysis of protein expression.

High performance anion exchange chromatography was performed on an ICS-5000 with a quaternary pump, a CarboPac PA1 4x250 mm column with a corresponding 4x50 mm guard column and pulsed amperometric detection (Thermo Fisher). The gradient used was: A = 1 mM NaOH in degassed water; C = 1 mM NaOH, 1M NaOAc in degassed water; 0 min (A: 95; C: 5); 20 min (A: 60; C: 40); 60 min (A: 60; C: 40); 63 min (A: 50; C: 50); 83 min (A: 50; C: 50); 87 min (A: 0; C: 100); 95 min (A: 0; C: 100); 97 min (A: 95; C: 5); 105 (A: 95; C: 5). Mixtures of commercial or synthetic standards (100-400 μM) were used.

Cell surface labeling, flow cytometry and in-gel fluorescence

K-562 cells stably transfected with pSBtet-WT-hAGX1-BH-T1, pSBtet-mut-hAGX1-WT-T1, pSBtet-mut-hAGX1-BH-T1 pSBtet-WT-hAGX1-BH-T2, pSBtet-mut-hAGX1-WT-T2, pSBtet-mut-hAGX1-BH-T2 were seeded into 6-well plates at a density of 500,000 cells/2 mL in growth medium without hygromycin and treated with 0.5 μg/mL doxycycline or left untreated. Cells were treated with doxycycline again after 24 h. After another 24 h, cells were counted, harvested and seeded at a density of 50,000 cells/0.2 mL (flow cytometry) or 200,000 cells/0.6 mL (in-gel fluorescence) fresh growth medium. Cells were treated with doxycycline again, and either caged GalNAc-1-phosphate analog **5**, Ac_4ManNAl (50 μM final concentration each) or DMSO vehicle. Cells were grown for another 20 h, harvested in a V-shaped 96 well plate and washed twice with 2% FBS in PBS (Cell Buffer, 0.2 mL).

For in-gel fluorescence, cells were resuspended in Cell Buffer (35 μL), treated with a solution of 50 μM $CuSO_4$, 300 μM BTTAA (Click Chemistry Tools, Scottsdale, USA), 2.5 mM sodium ascorbate, 2.5 mM aminoguanidinium chloride and 50 μM CF680 picolyl azide in cell buffer (35 μL), and incubated for 7 min at room temperature on an orbital shaker. The reaction was quenched by addition of 3 mM bathocuproinedisulfonic acid in PBS (35 μL). Cells were harvested, washed twice with Cell Buffer and once with PBS, and resuspended in ice-cold Lysis Buffer (50 mM Tris-HCl pH 8, 150 mM NaCl, 1% (v/v) Triton X-100, 0.5% (v/v) sodium deoxycholate, 0.1% (w/v) SDS, 1 mM $MgCl_2$, and 100 mU/μL benzonase (Merck) containing cOmplete protease inhibitors, 0.1 mL). Cells were lysed for 20 min at 4 °C on an orbital shaker and centrifuged (1500 g, 20 min, 4 °C). The supernatant was transferred to a new plate and

protein concentration was measured by BCA. For enzymatic digest, equal amounts of protein (typically 15 μg) were diluted to 40 μL with Lysis Buffer (PNGase F digest) or PBS (StcE digest), treated with PNGase F (2 U, Promega, Madison, USA) or StcE (50 nM) and incubated for 12 h at 37 °C (Malaker et al., 2019). The reaction was quenched by heating to 95 °C for 10 s with subsequent cooling at 4 °C. Loading buffer (a 1:1:1:0.5 (v/v/v/v) mixture of 1 M Tris-HCl pH 7.0, 10% (w/v) SDS, 80% (v/v) glycerol and 1 M DTT) was added, samples were heated at 95 °C for one minute, run on a 10% Criterion^{TM} gel (Bio-Rad, Hercules, USA) for SDS-PAGE, and imaged on an Odyssey CLx (LI-COR Biosciences, Lincoln, USA). Total protein content was assessed by Coomassie staining using Acquastain (Bulldog Bio, Portsmouth, USA). Another aliquot of each sample was used for protein expression control by western blot, using antibodies against VSV-G-tag, GAPDH (ab128915, abcam) or FLAG tag (mouse anti-FLAG M2, Sigma Aldrich) and secondary antibodies IRDye® 800CW Donkey anti-mouse IgG amd IRDye® 680RD Donkey anti-rabbit IgG. Background and contrast for gel and blot images were linearly and uniformly adjusted using either the LI-COR software or Photoshop 2020 (Adobe, San Jose, USA).

For flow cytometry, cells were resuspended in cell buffer (50 μL) and treated with a solution of 50 μM $CuSO_4$, 300 μM BTTAA, 2.5 mM sodium ascorbate, 2.5 mM aminoguanidinium chloride and 50 μM MB488 picolyl azide (Click Chemistry Tools) in Cell Buffer (50 μL), and incubated for 5 min at room temperature on an orbital shaker. The reaction was quenched by addition of 3 mM bathocuproinedisulfonic acid in PBS (50 μL). Cells were harvested, washed twice with cell buffer and once with PBS, Cells were then fixed with 0.5% (v/v) paraformaldehyde in PBS (100 μL) for 10 min at room temperature in the dark, harvested and washed with cell buffer once. Cells were permeabilized with 0.5% (v/v) Tween-20 in cell buffer (100 μL) for 10 min at room temperature. Cells were harvested, treated with mouse anti-VSV-G-tag (1:200) in 0.5% (v/v) Tween-20 in cell buffer (50 μL) for 30 min at room temperature, and washed with 0.1% (v/v) Tween-20 in cell buffer. Cells were treated with anti-mouse IgG 647 (1:200, Jackson ImmunoResearch, Cambridgeshire, UK) in 0.5% (v/v) Tween-20 in cell buffer (50 μL) for 30 min at room temperature, harvested, washed with 0.1% (v/v) Tween-20 in Cell Buffer and Cell Buffer without detergent. Flow cytometry was performed on an Accuri C6 flow cytometer (Becton Dickinson, Franklin Lakes, USA).

Proteomics

K-562 cells stably transfected with pSBtet-mut-hAGX1-WT-T1, pSBtet-mut-hAGX1-BH-T1, pSBtet-mut-hAGX1-WT-T2, or pSBtet-mut-hAGX1-BH-T2 were seeded into a T75 flask at 5,000,000 cells/15 mL in growth medium without hygromycin, and treated with 0.5 μg/mL doxycycline. Cells were treated with 10 mL growth medium and 0.5 μg/mL doxycycline after 24 h. After another 24 h, cells were counted, harvested and seeded at a density of 10,000,000 cells/25 mL in growth medium without hygromycin. Cells were treated with doxycycline again, and either caged GalNAc-1-phosphate analog **5** (50 μM) or DMSO vehicle. Cells were grown for another 20 h, harvested and washed with PBS (5 mL). Cell pellets were stored at −80 °C until use.

Cell pellets were resuspended in 500 μL Lysis Buffer (PBS with 1% (v/v) RapiGest and 1x Calbiochem Protease Inhibitors, Set III) and homogenized using a finger tip sonicator (output 1.0, 6x10 s strokes with 10 s breaks) on ice. Five to six mg protein per sample were treated with PNGase F (Promega, 5 μL of a 1:10 (v/v) dilution in PBS) and incubated for 4 h at 37 °C. The sample was diluted with PBS to a protein content of 2 mg/mL. Beads (UltraLink PLUS Streptavidin, 150 μL slurry = 75 μL settled resin) were washed with PBS twice (harvest 30 s at 1000 g in table top) in low-bind tubes (Eppendorf) and added to the lysate. Samples were incubated for 16 h at room temperature under rotation. The beads were harvested and the supernatant was discarded. The beads were washed sequentially with 0.1% RapiGest in PBS (3x), 6 M urea in PBS (3x), and PBS (2x), and resuspended in PBS (200 μL). Beads were treated with 10 μL 100 mM DTT (Thermo Fisher) in PBS (10 μL), and incubated at r.t. while shaking (950 rpm) for 30 min. Then, 500 mM iodoacetamide (Sigma Aldrich) in PBS (4 μL) was added and samples were shaken for another 30 min in the dark. Beads were harvested and washed with PBS and 50 mM ammonium bicarbonate in LC/MS-grade water (3x, ABC buffer). Beads were resuspended in ABC buffer (200 μL), treated with RapiGest to a final concentration of 0.05% (v/v), and trypsin (1.5 μg in 3 μL ABC buffer). Samples were shaken at 37 °C for 2-3 h, and another 1.5 μg trypsin was added. The reactions were shaken overnight at 37 °C. The beads were harvested, washed with ABC (200 μL) and with LC-MS grade water (3x200 μL) Supernatants and washes were combined and centrifuged (18000 g, 5 min, room temperature). The supernatants were transferred to new tubes and concentrated by Speedvac. Samples were desalted by C18 Zip tips, using 0.1% aq. formic acid (FA) as washing solution and stepwise elution with 50% MeCN/water with 0.1% FA (v/v) and 100% MeCN with 0.1% (v/v) FA.

Samples were analyzed by online nanoflow LC-MS/MS using an Orbitrap Fusion Tribrid mass spectrometer (Thermo Fisher) coupled to a Dionex Ultimate 3000 HPLC (Thermo Fisher). A portion of the sample (6.5 μL out of 8 μL for glycopeptide fractions and 2 μL out of 10 μL for peptide fractions) was loaded via autosampler isocratically onto a C18 nano pre-column using 0.1% formic acid in water ("Solvent A"). For pre-concentration and desalting, the column was washed with 2% ACN and 0.1% formic acid in water ("loading pump solvent"). Subsequently, the C18 nano pre-column was switched in line with the C18 nano separation column (75 μm x 250 mm EASYSpray (Thermo Fisher) containing 2 μm C18 beads) for gradient elution. The column was held at 40 °C using a column heater in the EASY-Spray ionization source (Thermo Fisher). The samples were eluted at a constant flow rate of 0.3 μL/min using a 90 minute gradient and a 140 minute instrument method. The gradient profile was as follows (min:% solvent B, 2% formic acid in acetonitrile) 0:3, 3:3, 93:35, 103:42, 104:95, 109:95, 110:3, 140:3. The instrument method used an MS1 resolution of 60,000 at FWHM 400 m/z, an AGC target of 3e5, and a mass range from 300 to 1,500 m/z. Dynamic exclusion was enabled with a repeat count of 3, repeat duration of 10 s, exclusion duration of 10 s. Only charge states 2-6 were selected for fragmentation. MS2s were generated at top speed for 3 s. HCD was performed on all selected precursor masses with the following parameters: isolation window of 2 m/z, 28%–30% collision energy, orbitrap (resolution of 30,000) detection, and an AGC target of 1e4 ions. ETD was performed if (a) the

precursor mass was between 300-1000 m/z and (b) 2 of 10 glyco and/or fingerprint ions (126.055, 138.055, 144.07, 168.065, 186.076, 204.086, 274.092, 292.103; 491.2241, 330.1554) were present at ± 0.1 m/z and greater than 5% relative intensity. ETD parameters were as follows: calibrated charge-dependent ETD times, 2e5 reagent target, precursor AGC target 1e4.

Data evaluation was performed with Byonic^{TM} (Protein Metrics, Cupertino, USA). For protein IDs, Uniprot human proteome (downloaded June 26, 2016) was used as a reference database. Search parameters included semi-specific cleavage specificity at the C-terminal site of R and K, with two missed cleavages allowed. Mass tolerance was set at 10 ppm for MS1s, 0.1 Da for HCD MS2s, and 0.35 Da for ETD MS2s. Methionine oxidation (common 2), asparagine deamidation (common 2), and N-terminal acetylation (rare 1) were set as variable modifications with a total common max of 3, rare max of 1. Cysteine carbamidomethylation was set as a fixed modification. Peptide hits were filtered using a 1% FDR. Additionally, a cut-off value of Log Prob = 5 was set for protein hits.

For glycopeptide analysis, search parameters included semi-specific cleavage specificity at the C-terminal site of R and K, with two missed cleavages allowed. Mass tolerance was set at 10 ppm for MS1s, 0.1 Da for HCD MS2s, and 0.35 Da for ETD MS2s. Methionine oxidation (common 2), asparagine deamidation (common 2), and N-terminal acetylation (rare 1) were set as variable modifications with a total common max of 2, rare max of 1. O-glycans were also set as variable modifications (common 2), using a custom database, whereby HexNAc, HexNAc-NeuAc, HexNAc-Hex, HexNAc-Hex-NeuAc, and HexNAc-Hex-NeuAc2 were searched with an additional 287.1371 m/z to account for the chemical modification. HCD was used to confirm that the peptides were glycosylated, whereas ETD spectra were used for site-localization of glycosylation sites. All spectra with these modifications were manually annotated.

Click & enrichment of HepG2 secretome:

HepG2-T1-KO and HepG2-T2-KO cells stably transfected with pSBtet-mut-hAGX1-WT-T1, pSBtet-mut-hAGX1-BH-T1, pSBtet-mut-hAGX1-WT-T2, or pSBtet-mut-hAGX1-BH-T2 were seeded into one 10 cm dish per treatment, using 8 mL growth medium without hygromycin B. Cells were directly induced with 0.5 μg/mL Dox. After 24 h, cells were Dox-induced again and fed with either caged GalNAc-1-phosphate analog **5** (25 μM) or DMSO vehicle. After another 24 h, the medium was aspirated, and cells were washed with pre-warmed serum-free low-Glc DMEM. Serum-free medium was added, cells were treated with Dox again, and either GalNAc-1-phosphate analog **5** or DMSO vehicle again. Cells were incubated for 20 h. Conditioned supernatant was collected and centrifuged for 5 min at 3000 g. The supernatant was concentrated to 2 mL using an Amicon Ultra-15 centrifuge filter (3 kDa MWCO, Millipore). Samples were stored at −80 °C until further use.

In a repeat experiment, HepG2-T1-KO stably transfected with pSBtet-hAGX13A-T1BH and HepG2-T2-KO cells stably transfected with pSBtet-mut-hAGX1-BH-T2 were treated with GalNAc-1-phosphate analog **5** and treated the same way.

Samples were treated with PNGase F (Promega, 5 μL of a 1:10 dilution in PBS) and incubated for 4 h at 37 °C. Samples were then treated in that order with 600 μM BTTAA, 300 μM $CuSO_4$, 2.5 mM sodium ascorbate, 2.5 mM aminoguanidinium chloride, and 50 μM biotin-DADPS-picolyl azide **10**.

The click reaction was allowed to proceed for 3 h at r.t. under inversion. The samples were transferred into 15 mL Falcon tubes and treated with 5 mL cold (−20 °C) methanol. Samples were left at −80 °C overnight, when a white precipitate had formed. Samples were centrifuged (3700 g, 20 min, 4 °C), the supernatant was discarded, and pellets were washed with 5 mL methanol twice, with centrifugation each time.

The supernatant was completely removed and the pellet air-dried. Samples were treated with 250 μL 0.1% (w/v) Rapigest. Samples were sonicated in a water bath for 25 min and centrifuged (3700 g, 5 min, 4 °C). The supernatants were transferred to a fresh tube, and pellets were treated with 250 μL 6 M urea in PBS. Samples were sonicated and centrifuged again, the pellets treated with 250 μL PBS, sonicated and centrifuged again. All supernatants from solubilization steps were combined, and samples were diluted with PBS to 2 mL. Protein concentration was determined by BCA.

Beads (UltraLink PLUS Streptavidin, 100 μL slurry = 50 μL settled resin per sample) were washed with PBS twice (harvest 30 s at 1000 g in table top) in low-bind tubes (Eppendorf) and treated with the protein solutions. Samples were incubated for 16 h at room temperature under rotation. The beads were harvested and the supernatant was discarded. The beads were treated as described (*27*). Briefly, beads were washed sequentially with 0.1% RapiGest in PBS (2x), 6 M urea in PBS (3x), and PBS (3x), and resuspended in PBS (200 μL). Beads were treated with 100 mM DTT (Thermo Fisher) in PBS (10 μL), and incubated at r.t. while shaking (950 rpm) for 30 min. Then, 500 mM iodoacetamide (Sigma Aldrich) in PBS (4 μL) was added and samples were shaken for another 30 min in the dark. Beads were harvested and washed with PBS and 50 mM ammonium bicarbonate in LC/MS-grade water (3x, ABC buffer). Beads were resuspended in ABC buffer (200 μL), treated with RapiGest to a final concentration of 0.05% (w/v), and trypsin (0.5 μg in 1 μL ABC buffer). Samples were shaken at 37 °C for 2-3 h, and another 0.5 μg trypsin was added. The reactions were shaken overnight at 37 °C. The beads were harvested, washed with ABC (200 μL) and with LC-MS grade water (3x200 μL). Supernatants were discarded, and the beads were treated with 2% (v/v) formic acid in water (150 μL). Samples were shaken at r.t. for 30 min, and the cleavage step was repeated. The beads were washed with water (100 μL) and 1:1 MeCN/2% aq. FA (100 μL). All supernatants were combined for each sample, centrifuged (18000 g, 5 min, room temperature) and concentrated by Speedvac. Samples were dissolved in 0.1% (v/v) formic acid in water and used for mass spectrometry (see above).

General organic synthesis

For spectral characterization, please see Methods S1.

Solvents and reagents were of commercial grade. Anhydrous solvents were obtained from a Dry Solvent System. Moisture-sensitive reactions were carried out in heat-dried glassware and under a nitrogen atmosphere. Thin layer chromatography was performed on Kieselgel 60 F254 glass plates pre-coated with silica gel (0.25 mm thickness). Spots were developed with sugar stain (0.1% (v/v) 3-methoxyphenol, 2.5% (v/v) sulfuric acid in EtOH) or ceric ammonium molybdate stain (5% (w/v) ammonium molybdate, 1% (w/v) cerium(II) sulfate and 10% (v/v) sulfuric acid in water) dipping solutions. Flash chromatography was carried out on Fluka Kieselgel 60 (230-400 mesh). Solvents were removed under reduced pressure using a rotary evaporator and high vacuum (1 mbar).

^{1}H, ^{13}C and 2D NMR spectra were measured with a Varian AS400 spectrometer or a Varian AS600 spectrometer at 25°C. Chemical shifts (σ) are reported in parts per million (ppm) relative to the respective residual solvent peaks ($CDCl_3$: σ 7.26 in ^{1}H and 77.16 in ^{13}C NMR; acetone-D_6: σ 2.05 in ^{1}H and 29.84 in ^{13}C NMR). Two-dimensional NMR experiments (HH-COSY, CH-HSQC) were performed to assign peaks in ^{1}H spectra. The following abbreviations are used to indicate peak multiplicities: s singlet; d doublet; dd doublet of doublets; dt doublet of triplets; m multiplet. Coupling constants (*J*) are reported in Hertz (Hz). High resolution mass spectrometry by electrospray ionization (ESI-HRMS) was performed at Stanford University Mass Spectrometry, with a micrOTOF-Q II hybrid quadrupole time-of-flight mass spectrometer (Bruker) equipped with an Agilent 1260 UPLC.

Synthesis of bis(*S*-acetyl-2-thioethyl) 3,4,6-tri-*O*-acetyl-2-deoxy-2-(5-hexynoyl)amido-α-D-galactopyranosyl phosphate (5)

To a stirred solution of lactol **SI-1** (100 mg, 250 μmol) in MeCN (0.5 mL) were added at 0°C phosphoramidite **SI-2** (Yu et al., 2012) (133 mg, 362 μmol) in 0.75 mL MeCN and 1*H*-tetrazole (27.1 mg, 388 μmol, 873 μL of a 3% (w/v) solution in MeCN). The reaction was warmed to room temperature and stirred for 1 h. Phosphoamidite **SI-2** (34 mg, 93 μmol) and 1*H*-tetrazole (235 μL, 104 μmol) were added to drive the reaction to completion. The mixture was stirred for 20 min at room temperature, cooled to 0 °C and treated with mCPBA (64.7 mg, 616 μmol). The reaction was stirred for 2 h at that temperature and quenched with 10% aq. Na_2SO_3 (10 mL). The solution was extracted with CH_2Cl_2 (5x10 mL), the combined organic layers were dried over $MgSO_4$, filtered and concentrated. The residue was purified by flash chromatography (hexanes/EtOAc 1:0 to 1:1 with 0.5% (v/v) NEt_3) to give phosphotriester **5** (101 mg, 147 μmol, 59%) as a clear oil. R_f (hexanes/EtOAc with 0.5% (v/v) NEt_3, TLC plates pre-neutralized with hexanes/2% NEt_3) = 0.25.

Synthesis of Bis(*S*-acetyl-2-thioethyl) 3,4,6-tri-*O*-acetyl-2-deoxy-2-(2-(*S*)-methyl-5-hexynoyl)amido-α-D-galactopyranosyl phosphate (6)

To a stirred solution of lactol **SI-3** (30 mg, 73 μmol) in MeCN (0.73 mL) were added at 0 °C phosphoramidite **SI-2** (30 mg, 81 μmol) in 0.4 mL toluene and 1*H*-tetrazole (9.1 mg, 130 μmol, 252 μL of a 3% (w/v) solution in MeCN). The reaction was warmed to room temperature and stirred for 1 h. Phosphoamidite **SI-2** (30 mg, 81 μmol) was added to drive the reaction to completion. The mixture was stirred for 20 min at room temperature, cooled to −20 °C and treated with mCPBA (18.6 mg, 108 μmol). The reaction was stirred for 2 h at that temperature and quenched with 10% aq. Na_2SO_3 (10 mL). The solution was extracted with CH_2Cl_2 (5x10 mL), the combined organic layers were dried over $MgSO_4$, filtered and concentrated. The residue was purified by flash chromatography (hexanes/EtOAc 1:0 to 5:1 to 3:1to 1:1 with 0.5% (v/v) NEt_3) and size exclusion chromatography (Sephadex LH-20, solvent CH_2Cl_2 4:1) to give phosphotriester **6** (12.5 mg, 18 μmol, 25%) as a clear oil.

Synthesis of Bis(*S*-acetyl-2-thioethyl) 3,4,6-tri-*O*-acetyl-2-deoxy-2-(2-(*R*)-methyl-5-hexynoyl)amido-α-D-galactopyranosyl phosphate (7)

To a stirred solution of lactol **SI-4** (71 mg, 171 μmol) in MeCN (0.73 mL) were added at 0 °C phosphoramidite **SI-2** (75 mg, 202 μmol) in 0.4 mL toluene and 1*H*-tetrazole (21.5 mg, 307 μmol, 716 μL of a 3% (w/v) solution in MeCN). The reaction was warmed to room temperature and stirred for 1 h. Phosphoamidite **SI-2** (30 mg, 81 μmol) was added to drive the reaction to completion. The mixture was stirred for 20 min at room temperature, cooled to −20 °C and treated with mCPBA (50 mg, 290 μmol). The reaction was stirred for 2 h at that temperature and quenched with 10% aq. Na_2SO_3 (10 mL). The solution was extracted with CH_2Cl_2 (5x10 mL), the combined organic layers were dried over $MgSO_4$, filtered and concentrated. The residue was purified by flash chromatography (hexanes/EtOAc 1:0 to 5:1 to 3:1to 1:1 with 0.5% (v/v) NEt_3) and size exclusion chromatography (Sephadex LH-20, solvent CH_2Cl_2 4:1) to give phosphotriester **7** (11 mg, 16 μmol, 9%) as a clear oil.

3-Hydroxypropyl (6-azidomethyl)nicotinate (SI-7)

To a stirred solution of ester **SI-5** (58 mg, 0.30 mmol) in 5:2 MeOH/water (1.5 mL) was added at room temperature triethylamine (0.3 mL). The reaction was warmed to 50 °C and stirred for 20 h at that temperature, when the starting material was converted to a lower-running spot (TLC CH_2Cl_2/MeOH 10:1). The solvents were evaporated, and the residue was co-evaporated with toluene (3x5 mL) to give the intermediate triethylammonium salt as a yellow oil.

To a stirred solution of the intermediate triethylammonium salt in CH_2Cl_2 (1.5 mL) were added at room temperature triethylamine (164 μL, 1.18 μmol), EDC hydrochloride (116 mg, 0.6 mmol) and *N*-hydroxysuccinimide (52 mg, 0.45 mmol). The mixture was stirred for 2 h, and 3-amino-1-propanol **SI-6** (72 mg, 0.95 mmol) was added. The reaction was stirred for 20 h, diluted with CH_2Cl_2 (5 mL), and filtered through cotton wool. The solution was concentrated, and the residue was purified by flash chromatography (CH_2Cl_2/MeOH 99:1 to 95:5) to give amide **SI-7** (26 mg, 111 μmol, 37% over two steps) as a clear oil.

1,3,4,6-Tri-*O*-acetyl-2-deoxy-2-(5-hexynoyl)amido-αβ-D-glucopyranoside (9)

To a stirred solution of ammonium salt **SI-10** (200 mg, 0.52 mmol) in 5:2 CH_2Cl_2 (2.6 mL) were added at room temperature active ester **SI-9** (130 mg, 0.63 mmol) and triethylamine (145 µL, 1.04 mmol). The reaction was stirred for 16 h and treated with EDC hydrochloride (99.8 mg, 0.52 mmol). The mixture was stirred for 48 h, the reaction was diluted with EtOAc (10 mL), washed with 1 N HCl (2x5 mL), sat. aq. $NaHCO_3$ (5 mL) and brine (5 mL). The combined organic layers were dried over $MgSO_4$, filtered and concentrated. The residue was purified by flash chromatography (hexanes/EtOAc 2:1 to 1:2 to 1:4 to give amide **9** (72 mg, 0.16 mmol, 31%) as a clear oil.

Biotin-PEG$_4$-dialkoxydiphenylsilane-picolyl azide (10)

To a stirred solution of alcohol **SI-8** (10 mg, 17.7 µmol) in CH_2Cl_2 (0.5 mL) were added at room temperature triethylamine (50 µL, 360 µmol) and dichlorodiphenylsilane (22.4 mg, 88.5 µmol). The mixture was stirred for 3 h, and alcohol **SI-7** (36 mg,153 µmol in 0.2 mL CH_2Cl_2) was added. The reaction was stirred for 20 h, quenched with sat. aq. $NaHCO_3$ (3 mL) and diluted with CH_2Cl_2 (5 mL). The layers were separated, and the aqueous phase was extracted with CH_2Cl_2 (5x5 mL). The combined organic layers were dried over Na_2SO_4, filtered and concentrated. The residue was purified by flash chromatography (CH_2Cl_2/MeOH 1:0 to 95:5 with 1% NEt_3, then EtOAc/MeOH 1:0 to 9:1 with 1% NEt_3) and size exclusion chromatography (Sephadex LH-20, CH_2Cl_2/MeOH 1:1) to give diphenyldisiloxane **10** (5 mg, 5.1 µmol, 29%) as a clear oil.

QUANTIFICATION AND STATISTICAL ANALYSIS

The number of replicates for each experiment is specified in Figure legends. Flow cytometry experiments were run in three independent replicates on three different days. Statistical analysis was performed by two-tailed ratio paired t test, and *P value*s are indicated in figures.

Molecular Cell, Volume *78*

Supplemental Information

Bump-and-Hole Engineering Identifies Specific Substrates of Glycosyltransferases in Living Cells

Benjamin Schumann, Stacy Alyse Malaker, Simon Peter Wisnovsky, Marjoke Froukje Debets, Anthony John Agbay, Daniel Fernandez, Lauren Jan Sarbo Wagner, Liang Lin, Zhen Li, Junwon Choi, Douglas Michael Fox, Jessie Peh, Melissa Anne Gray, Kayvon Pedram, Jennifer Jean Kohler, Milan Mrksich, and Carolyn Ruth Bertozzi

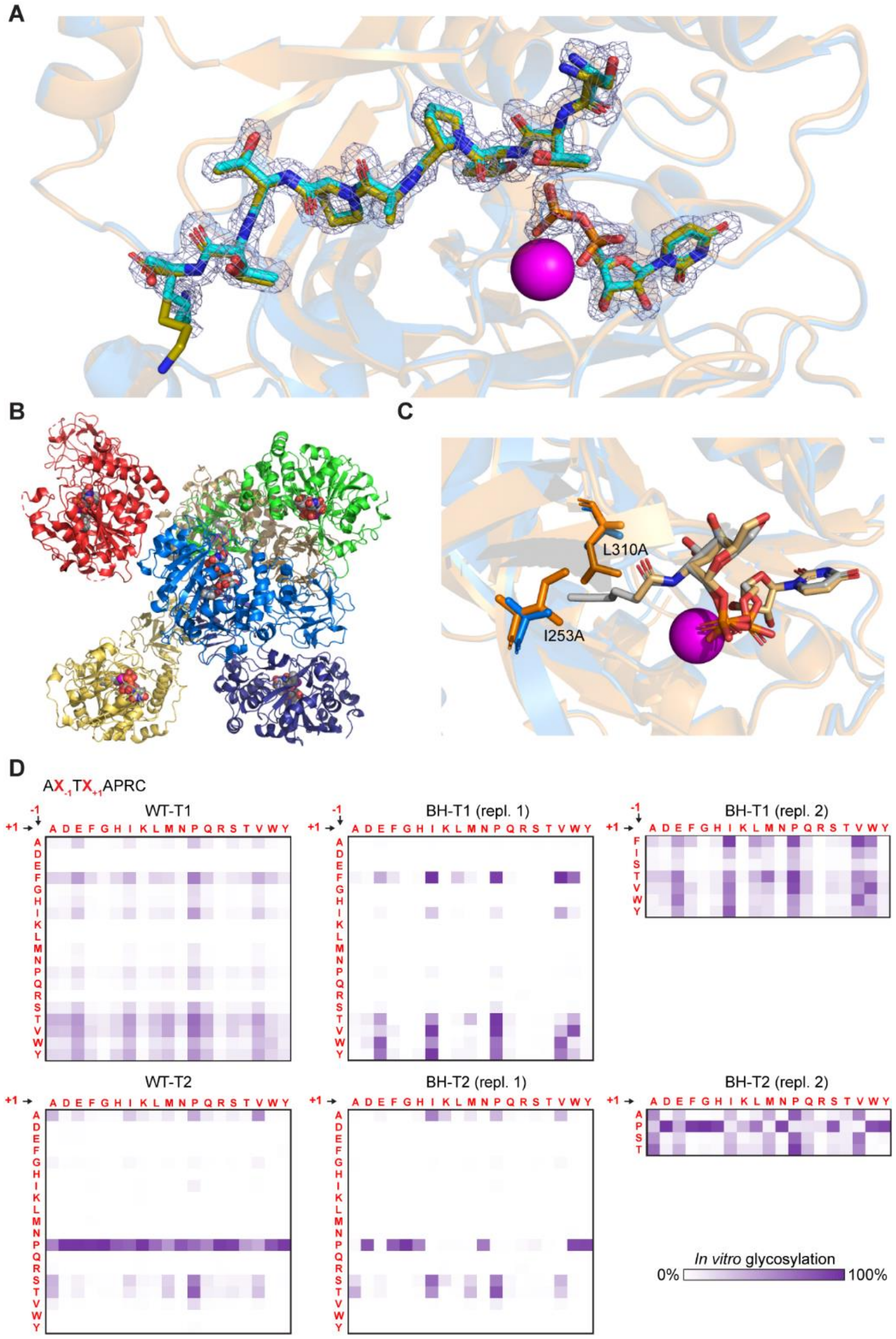

Figure S1. Validation of BH-GalNAc-Ts, Related to Figure 2 and Table 1.

(A) Overlay of ligands bound in BH-T2/EA2/UDP/Mn2+ (ligands cyan) and a published crystal structure of WT-T2/EA2/UDP/Mn2+ (ligands olive, PDB 2FFU), with electron density (map rendered at 1 σ and carved at 1.6 Å) taken from the BH-T2 co-crystal structure. Mn2+ ions are colored in magenta and overlay completely.

(B) Crystal structure of BH-T2/1/Mn2+ in the hexameric unit cell. UDP-GalNAc analog **1** is rendered in sphere representation.

(C) Superposition of the active sites of WT-T2 (PDB 4D0T, orange) with UDP-GalNAc (light brown) and BH-T2 (blue) with UDP-GalNAc analog **1** (grey). Gatekeeper residues are rendered in stick representation. Mn^{2+} ions are magenta and overlay completely.

(D) Substrate specificities of BH-T1 and BH-T2 and comparison with WT enzymes as determined in an *in vitro* glycosylation assay with detection by SAMDI-MS. WT data corresponds with reported substrate specificities, and two replicates are shown for BH enzymes with the full library or a focused sub-library.

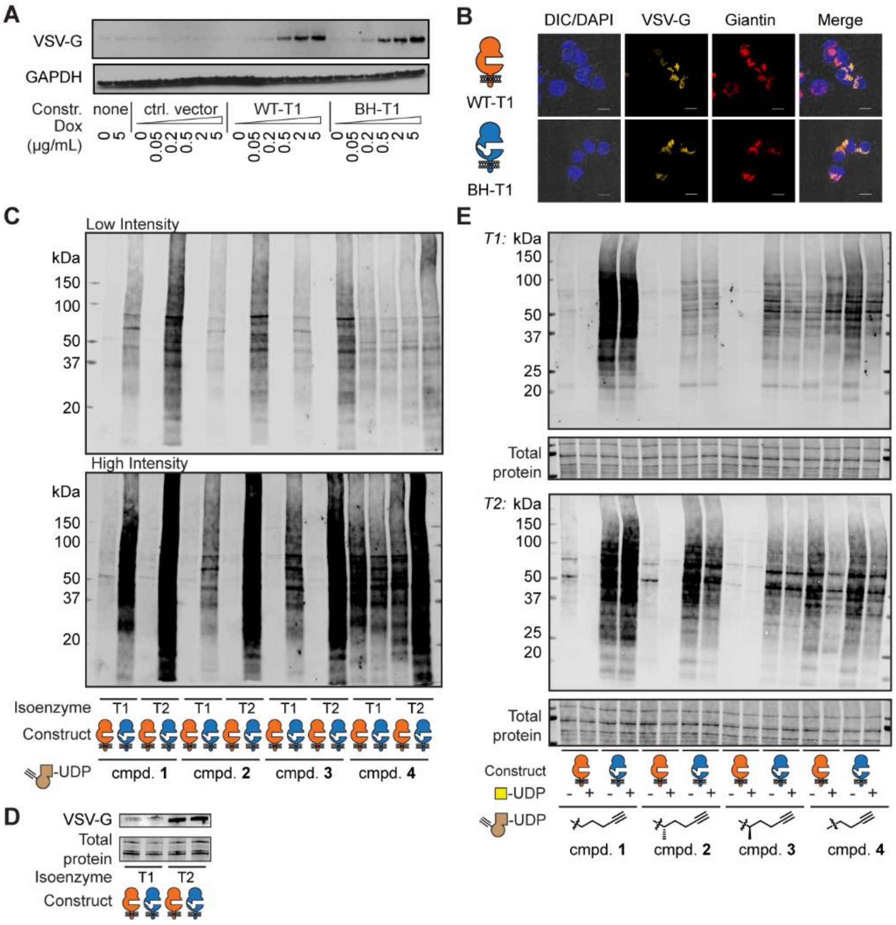

Figure S2. Localization of BH-GalNAc-T1 to the Golgi compartment and glycosylation of proteins, Related to Figure 3.

(A) WT-T1 and BH-T1 are expressed by stably transfected HepG2 cells in a Dox-inducible fashion.

(B) Fluorescence microscopy of HepG2 cells stably transfected with T1 constructs, induced with 2 µg/mL Dox and subsequently stained. Inset: magnification of a single cell. Scale bar, 10 µm.

(C) Streptavidin blot from Figure 3C depicted in two different intensities.

(D) Expression of GalNAc-Ts in lysates used in (C).

(E) *In vitro* glycosylation was repeated with a membrane fraction from untransfected HepG2 cells, using soluble GalNAc-Ts to perform glycosylations (Choi et al., 2019). Reactions were performed with or without a two-fold excess of UDP-GalNAc over UDP-sugars **1**, **2**, **3** and **4**.

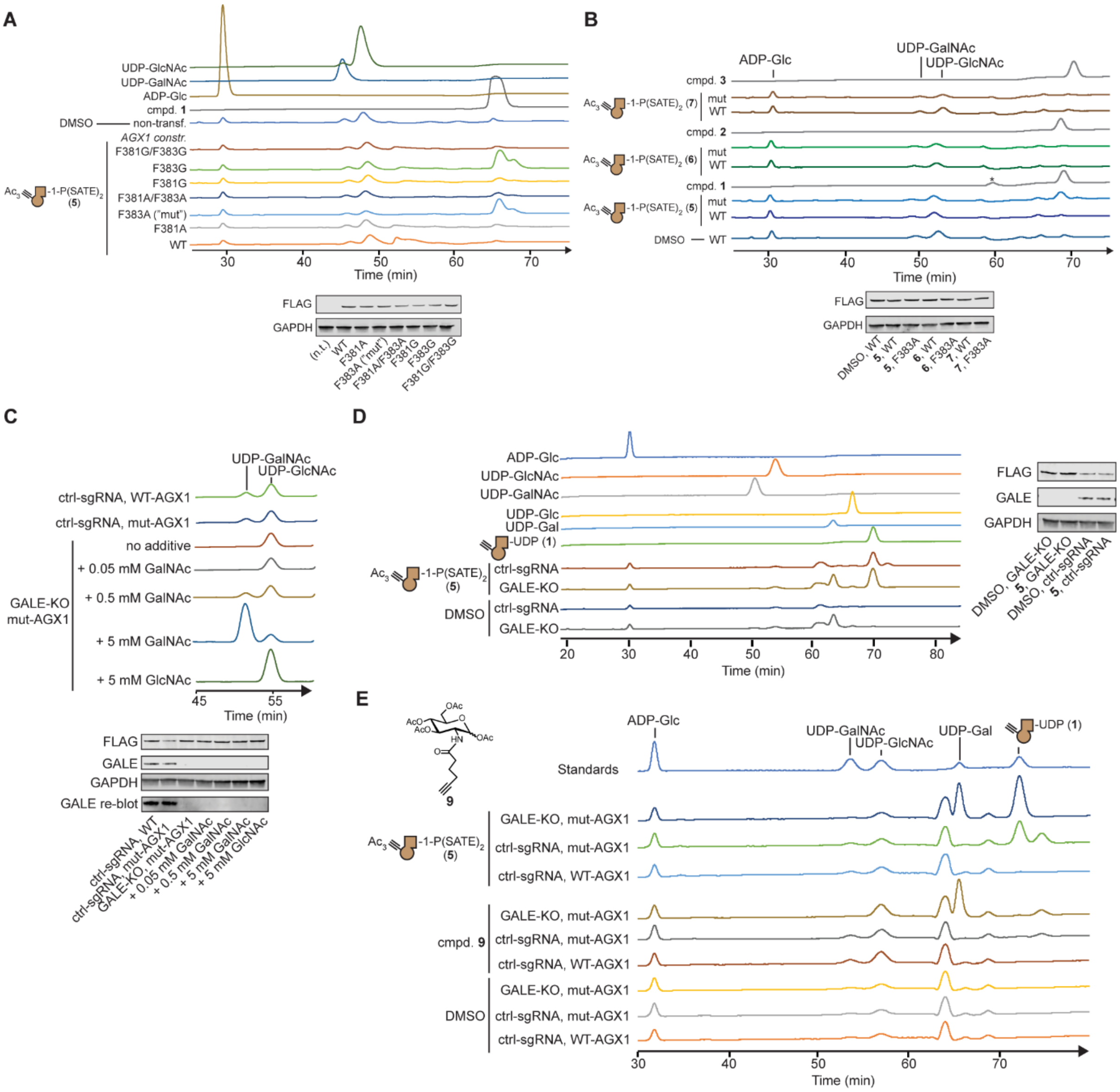

Figure S3. Biosynthesis of UDP-GalNAc analog 1 by mut-AGX1 and epimerization by GALE, Related to Figure 4.

(A) HPAEC-PAD traces of extracts from HEK293T cells transiently expressing AGX1 constructs, and fed with GalNAc-1-phosphate analog **5** or DMSO. All traces are normalized to the retention time of ADP-Glc as an external standard. Data is of a single experiment.

(B) Full traces of the data displayed in Fig. 4B. Asterisk indicates an artefact from solvent filling.

(C) HPAEC-PAD traces of GALE-KO or control sgRNA-transduced K-562 cells stably transfected with the indicated FLAG-tagged AGX1 constructs and fed with different concentrations of GalNAc or GlcNAc. Expression of AGX1 (FLAG) and GALE were analyzed by Western blot. Samples were re-blotted with a higher concentration of GALE antibody to assure absence of GALE in KO cells. Data are from one experiment.

(D) Cells were fed with DMSO or compound **5**, and UDP-sugar production was measured by HPAEC-PAD. GALE-KO contain elevated levels of UDP-Gal as cells are supplemented with galactose to maintain viability and UDP-Gal cannot be epimerized to UDP-Glc. Expression levels of AGX1 (FLAG) and GALE are analyzed by Western blot. Data are of one representative out of two independent experiments.

(E) GALE-KO or control sgRNA-transduced

(F) K-562 cells stably transfected with the indicated AGX1 constructs were fed with the indicated compounds, and UDP-sugar production was measured by HPAEC-PAD. Data are from one experiment.

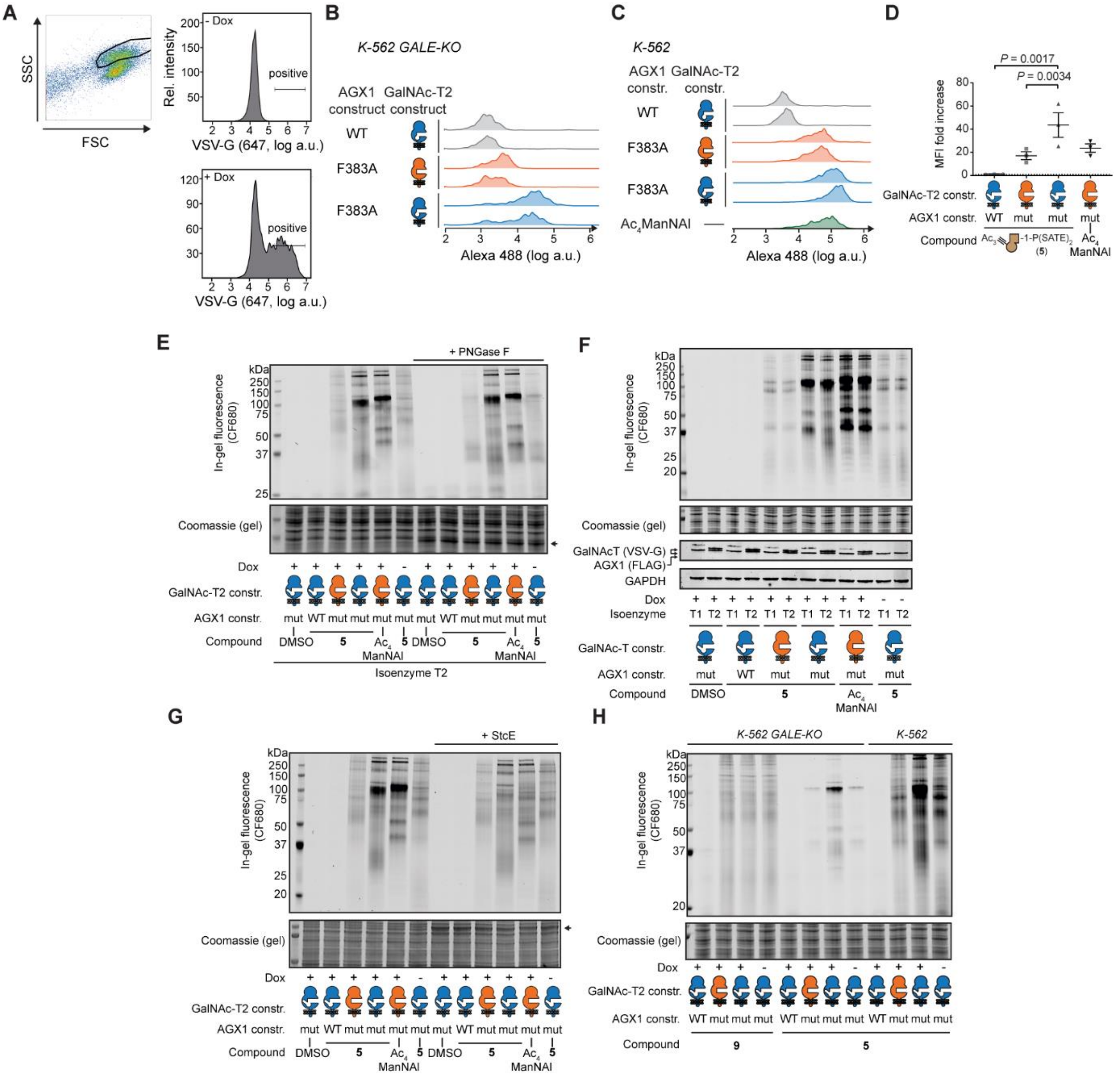

Figure S4. Bioorthogonal, GalNAc-T-selective cell surface labeling, Related to Figure 5.

(A) Gating scheme for flow cytometry experiments, and in-gel fluorescence experiments.

(B) Primary flow cytometry data of the experiment in Figure 5C. Two technical replicates are shown.

(C) Primary flow cytometry data of K-562 cells after induction with 0.5 µg/mL Dox and gating on VSV-G-positive cells. Two technical replicates are shown for cells treated with compound **5**.

(D) Statistical analysis of the experiment in (C). Data are represented as individual values from three independent experiments, mean ± SEM of MB488 median fluorescence intensity of VSV-G positive cells. Statistical analysis was performed by two-tailed ratio paired t-test.

(E) Cells expressing AGX1 and GalNAc-T2 constructs were labeled as in Figure 5C and analyzed by in-gel fluorescence. Treatment of lysates with PNGase F significantly shifts certain background bands to lower molecular weight. Arrow indicates PNGase F band in Coomassie stain.

(F) Full gel of the experiment depicted in Figure 5C. The sialic acid precursor $Ac_4ManNAl$ was used as a positive control, and the effect of omitting Dox was investigated. In-gel fluorescence and Coomassie staining are from one gel and expression analyses are from one separate Western Blot.

(G) Treatment of lysates prepared as in (A) with the glycoprotease StcE. Arrow indicates StcE band in Coomassie stain.

(H) Dissecting GlcNAc vs. GalNAc labeling by using probes **5** and **9**. K-562 GALE-KO cells expressing AGX1 and GalNAc-T2 constructs were treated with Dox or left untreated, and fed with compounds **5** or **9**. Labeling was performed as in Figure 5C. K-562 cells prepared as in (A) were used to compare labeling patterns. Data are from one representative out of three independent experiments.

At least 500 gated cells were used for analysis per sample in (B) and (C).

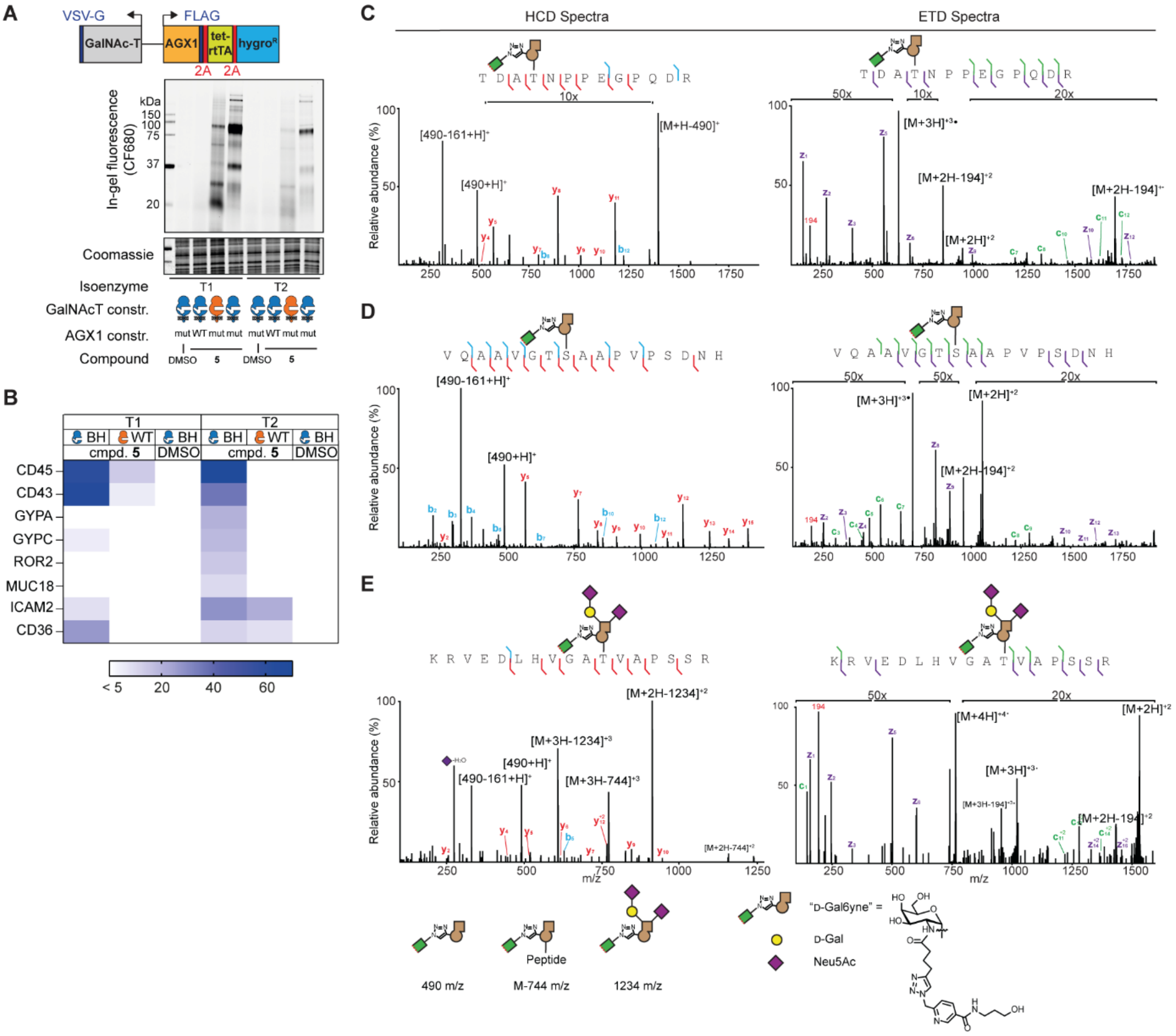

Figure S5. GalNAc-T bump-and-hole pairs selectively label O-glycosylated proteins, Related to Figure 5.

(A) In-gel fluorescence of cell surface proteins in K-562 cells stably expressing GalNAc-T and AGX1 constructs. Cells and samples were treated as in Figure 5C.

(B) Mass spectrometry proteomics. Heat map represents log prob of detected proteins after enrichment of cell surface proteins of K-562 cells expressing mut-AGX1 and indicated GalNAc-T constructs and fed with the indicated compounds.

(C-E) Exemplary mass spectra from glycopeptides after modification by BH-T2. HCD (mainly glycan fragmentation) and ETD (mainly peptide fragmentation) spectra are shown, and ions are annotated. y and b ions in HCD spectra were devoid of glycan.

(C) Glycosylation at Thr28 of STC2 as a newly-identified T2-specific modification (Luo et al., 2005).

(D) Confirmation of Ser308 as a T2-specific modification of ApoE.

(E) Extension of chemically tagged GalNAc by elaborating glycosyltransferases on T39 of SERPIN5A. Legend depicts tentative structural assignments of neutral loss fragments. Loss of 161 m/z in HCD and 194 m/z peak in ETD likely depicts fragment masses of the triazole-based linker.

Methods S1, Related to STAR Methods. Characterization of synthetic compounds.

Bis(*S*-acetyl-2-thioethyl) 3,4,6-tri-*O*-acetyl-2-deoxy-2-(5-hexynoyl)amido-α-D-galactopyranosyl phosphate (5)

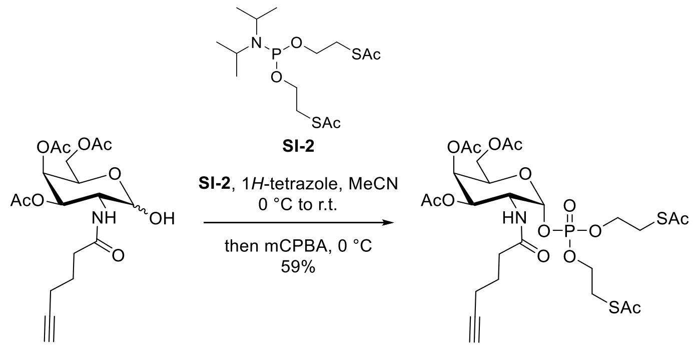

SI-1 **5**

^{1}H NMR (600 MHz, $CDCl_3$) δ 6.24 (d, *J* = 9.2 Hz, 1H), 5.75 (dd, *J* = 5.6, 3.3 Hz, 1H), 5.44 (dd, *J* = 3.2, 1.4 Hz, 1H), 5.18 (dd, *J* = 11.5, 3.2 Hz, 1H), 4.81 – 4.60 (m, 1H), 4.40 (td, *J* = 6.6, 1.4 Hz, 1H), 4.29 – 3.93 (m, 4H), 3.34 – 3.06 (m, 6H), 2.37 – 2.30 (m, 8H), 2.28 – 2.17 (m, 2H), 2.15 (s, 3H), 2.03 (s, 3H), 1.99 (s, 3H), 1.95 (t, *J* = 2.6 Hz, 1H), 1.88 – 1.76 (m, 2H); ^{13}C NMR (150 MHz, $CDCl_3$) δ 195.3, 194.9, 172.8, 170.7, 170.4, 170.2, 97.3, 97.3, 83.4, 69.5, 69.3, 68.8, 67.4, 67.3, 66.9, 66.8, 66.6, 66.5, 66.5, 66.5, 61.5, 47.6, 47.6, 47.5, 47.4, 34.8, 34.7, 30.8, 30.7, 30.6, 29.2, 29.2, 29.2, 24.1, 24.0, 20.8, 20.7, 20.6, 17.8, 17.7; HRMS (ESI) calcd. for $C_{26}H_{38}NO_{14}PS_2Na$ (M+Na$^+$) 706.1369 found 706.1349 *m/z*.

Bis(*S*-acetyl-2-thioethyl) 3,4,6-tri-*O*-acetyl-2-deoxy-2-(2-(*S*)-methyl-5-hexynoyl)amido-α-D-galactopyranosyl phosphate (6)

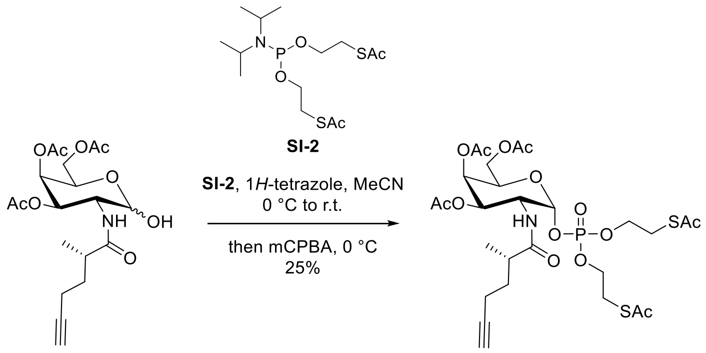

SI-3 **6**

^{1}H NMR (400 MHz, acetone-D_6) δ 7.36 (d, *J* = 8.3 Hz, 1H), 5.78 (dd, *J* = 6.0, 3.4 Hz, 1H), 5.50 (dd, *J* = 3.2, 1.4 Hz, 1H), 5.17 (dd, *J* = 11.8, 3.2 Hz, 1H), 4.71 – 4.44 (m, 2H), 4.31 – 4.03 (m, 6H), 3.22 (t, *J* = 6.5 Hz, 4H), 2.62 – 2.44 (m, 1H), 2.44 – 2.28 (m, 7H), 2.19 – 2.11 (m, 5H), 2.01 (s, 3H), 1.94 (s, 3H), 1.90 – 1.75 (m, 1H), 1.61 – 1.49 (m, 1H), 1.12 (d, *J* = 6.9, 1.6 Hz, 3H); ^{13}C NMR (100 MHz, acetone-D_6) δ 195.2, 177.5, 176.5, 170.7, 170.6, 170.4, 97.6, 84.3, 70.2, 69.4, 67.9, 67.7, 66.9, 62.4, 48.1, 40.3, 33.8, 20.7, 20.6, 18.0, 16.8; HRMS (ESI) calcd. for $C_{27}H_{40}NO_{14}PS_2Na$ (M+Na$^+$) 720.1526 found 720.1523 *m/z*.

Bis(*S*-acetyl-2-thioethyl) 3,4,6-tri-*O*-acetyl-2-deoxy-2-(2-(*R*)-methyl-5-hexynoyl)amido-α-D-galactopyranosyl phosphate (7)

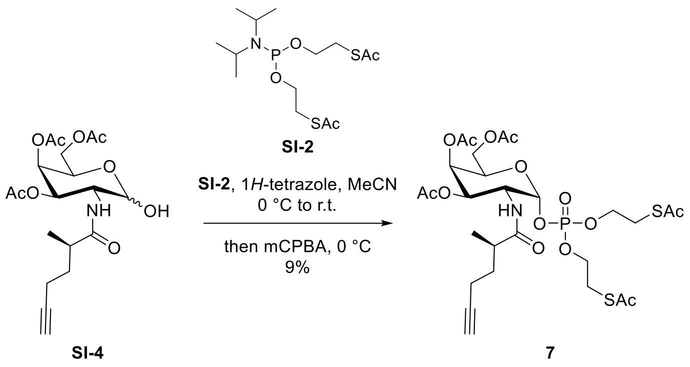

^{1}H NMR (600 MHz, acetone-D$_6$) δ 7.35 (d, *J* = 8.2 Hz, 1H), 5.81 (m, 1H), 5.50 (s, 1H), 5.18 (m, 1H), 4.59 – 4.43 (m, 2H), 4.33 – 4.01 (m, 6H), 3.31 – 3.07 (m, 4H), 2.55 – 2.45 (m, 1H), 2.40 – 2.30 (m, 7H), 2.25 – 2.12 (m, 5H), 2.01 (s, 3H), 1.97 – 1.85 (m, 4H), 1.57 – 1.48 (m, 1H), 1.09 (d, *J* = 7.0 Hz, 3H); ^{13}C NMR (150 MHz, acetone-D$_6$) δ 195.2, 176.6, 170.6, 170.4, 97.6, 97.6, 84.7, 70.1, 69.5, 68.0, 67.8, 67.1, 66.9, 62.4, 48.3, 40.4, 33.4, 30.6, 30.6, 20.3, 20.6, 18.3, 16.9; HRMS (ESI) calcd. for $C_{27}H_{40}NO_{14}PS_2Na$ (M+Na$^+$) 720.1526 found 720.1515 *m/z*.

3-Hydroxypropyl (6-azidomethyl)nicotinate (SI-7)

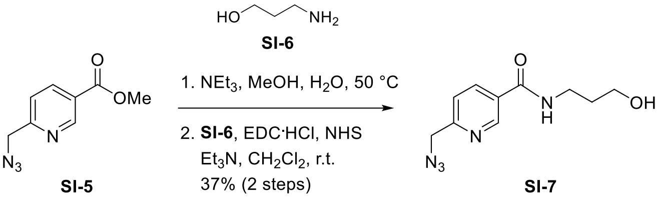

R$_f$ (CH_2Cl_2/MeOH 20:1) = 0.3. ^{1}H NMR (400 MHz, $CDCl_3$) δ 9.28 (d, *J* = 2.3 Hz, 1H), 8.49 (dd, *J* = 8.1, 2.3 Hz, 1H), 7.77 (d, *J* = 8.1 Hz, 1H), 7.44 (br s, 1H), 4.88 (s, 2H), 4.13 (t, *J* = 5.5 Hz, 2H), 3.99 (q, *J* = 5.9 Hz, 2H), 2.70 (br s, 1H), 2.25 – 2.11 (m, 2H); ^{13}C NMR (100 MHz, $CDCl_3$) δ 165.9, 158.8, 147.9, 136.4, 129.4, 121.8, 60.8, 55.4, 38.2, 31.7. HRMS (ESI) calcd. for $C_{10}H_{13}N_5O_2Na$ (M+Na$^+$) 258.0966 found 258.0968 *m/z*.

1,3,4,6-Tri-*O*-acetyl-2-deoxy-2-(5-hexynoyl)amido-αβ-D-glucopyranoside (9)

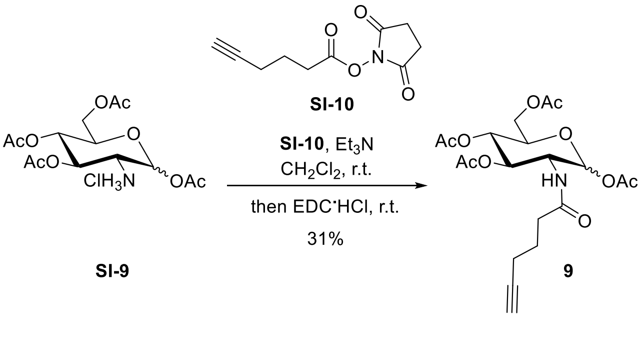

^{1}H NMR (400 MHz, $CDCl_3$) δ 5.88 (d, *J* = 10.2 Hz, 1H), 5.69 (d, *J* = 8.7 Hz, 1H), 5.30 – 5.01 (m, 2H), 4.43 – 4.20 (m, 2H), 4.11 (dd, *J* = 12.5, 2.2 Hz, 1H), 3.83 (ddd, *J* = 9.9, 4.7, 2.2 Hz, 1H), 2.32 – 2.15 (m, 4H), 2.10 (s, 3H), 2.07 (s, 3H), 2.03 (d, *J* = 1.5 Hz, 6H), 1.95 (t, *J* = 2.6 Hz, 1H), 1.77 (p, *J* = 7.0 Hz, 2H). ^{13}C NMR (100 MHz, $CDCl_3$) δ 172.4, 171.3, 170.8, 169.6, 169.5, 92.6, 83.2, 72.9, 72.6, 69.6, 68.1, 61.8, 52.8, 34.9, 24.0, 21.0, 20.8, 20.8, 20.7, 17.6. HRMS (ESI) calcd. for $C_{20}H_{27}NO_{10}Na$ (M+Na$^+$) 464.1532 found 464.1513 *m/z*.

Biotin-PEG$_4$-dialkoxydiphenylsilane-picolyl azide (10)

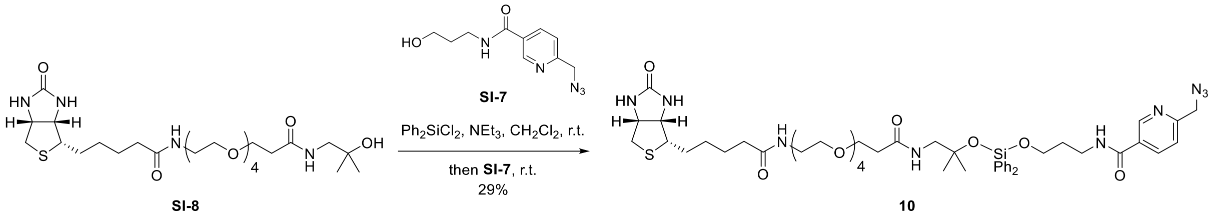

R_f (CH_2Cl_2/MeOH 10:1 with 1% NEt_3) = 0.55. ^1H NMR (600 MHz, $CDCl_3$) δ 8.93 (d, *J* = 2.2 Hz, 1H), 8.05 (dd, *J* = 8.1, 2.3 Hz, 1H), 7.66 – 7.59 (m, 4H), 7.44 – 7.40 (m, 2H), 7.39 – 7.33 (m, 4H), 7.28 (d, *J* = 7.5 Hz, 1H), 7.24 – 7.20 (m, 1H), 6.68 (s, 1H), 6.58 (s, 1H), 5.55 (s, 1H), 4.68 (s, 1H), 4.52 (s, 2H), 4.49 – 4.43 (m, 1H), 4.32 – 4.25 (m, 1H), 3.90 (t, *J* = 5.8 Hz, 2H), 3.70 (t, *J* = 6.1 Hz, 2H), 3.61 – 3.52 (m, 16H), 3.43 – 3.38 (m, 2H), 3.36 (d, *J* = 6.1 Hz, 2H), 3.13 (td, *J* = 7.3, 4.5 Hz, 1H), 2.90 (dd, *J* = 12.8, 5.0 Hz, 1H), 2.69 (d, *J* = 12.7 Hz, 1H), 2.43 (t, *J* = 6.0 Hz, 2H), 2.20 (td, *J* = 7.3, 3.1 Hz, 2H), 1.99 – 1.90 (m, 2H), 1.78 – 1.67 (m, 4H), 1.49 – 1.41 (m, 2H), 1.25 (s, 6H). HRMS (ESI) calcd. for $C_{47}H_{67}N_9O_{10}SSiNa$ (M+Na$^+$) 1000.4399 found 1000.4363 *m/z*.

NMR Spectra

^{1}H NMR, $CDCl_3$, 600 MHz

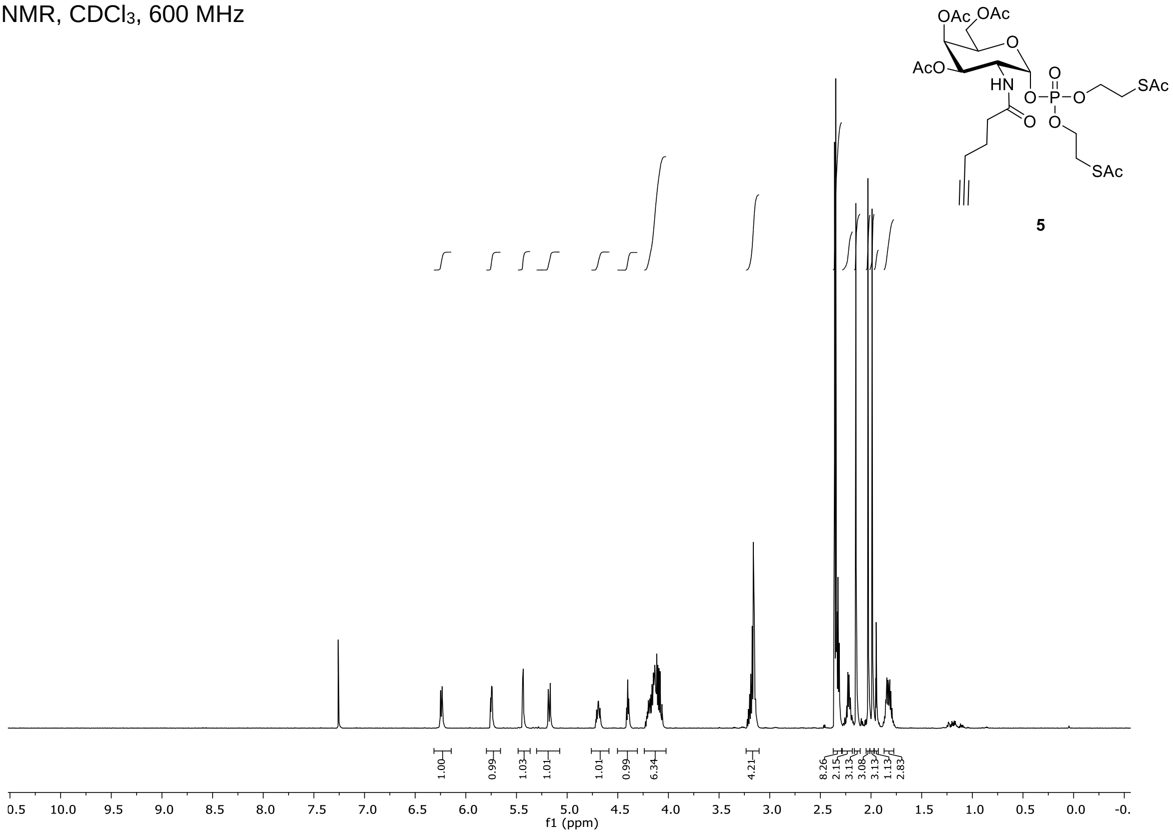

^{13}C NMR, $CDCl_3$, 150 MHz

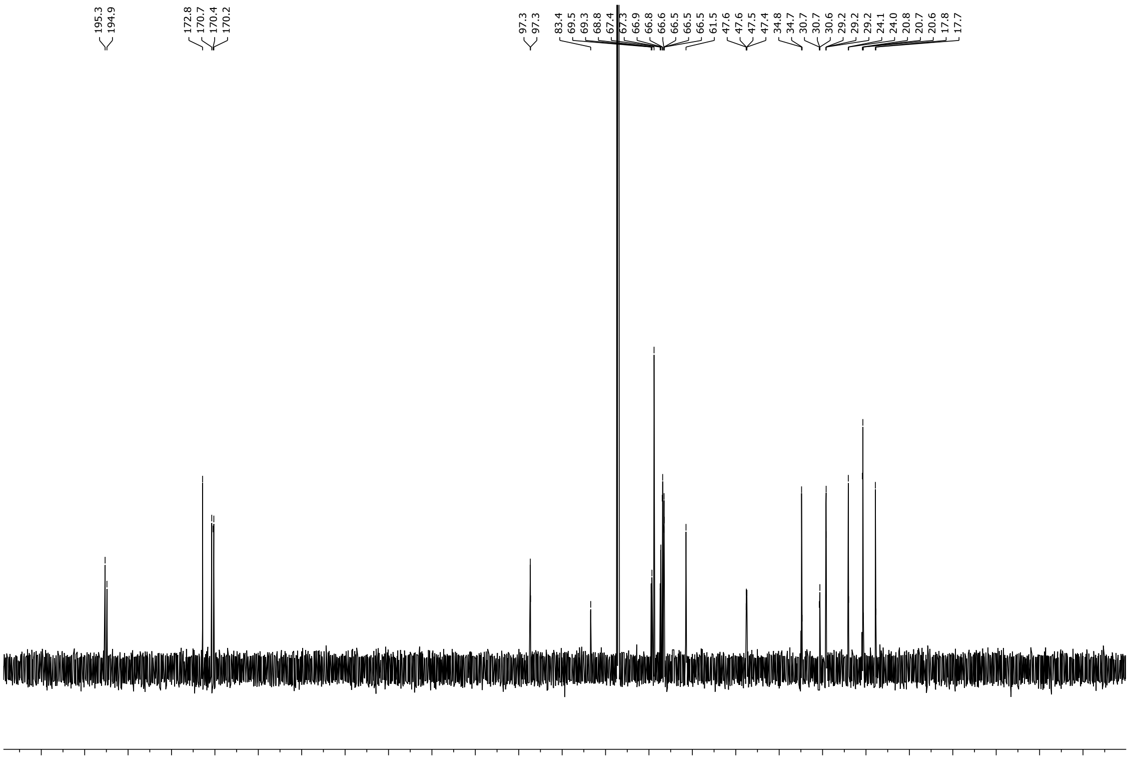

^{1}H NMR, acetone-D_6, 400 MHz

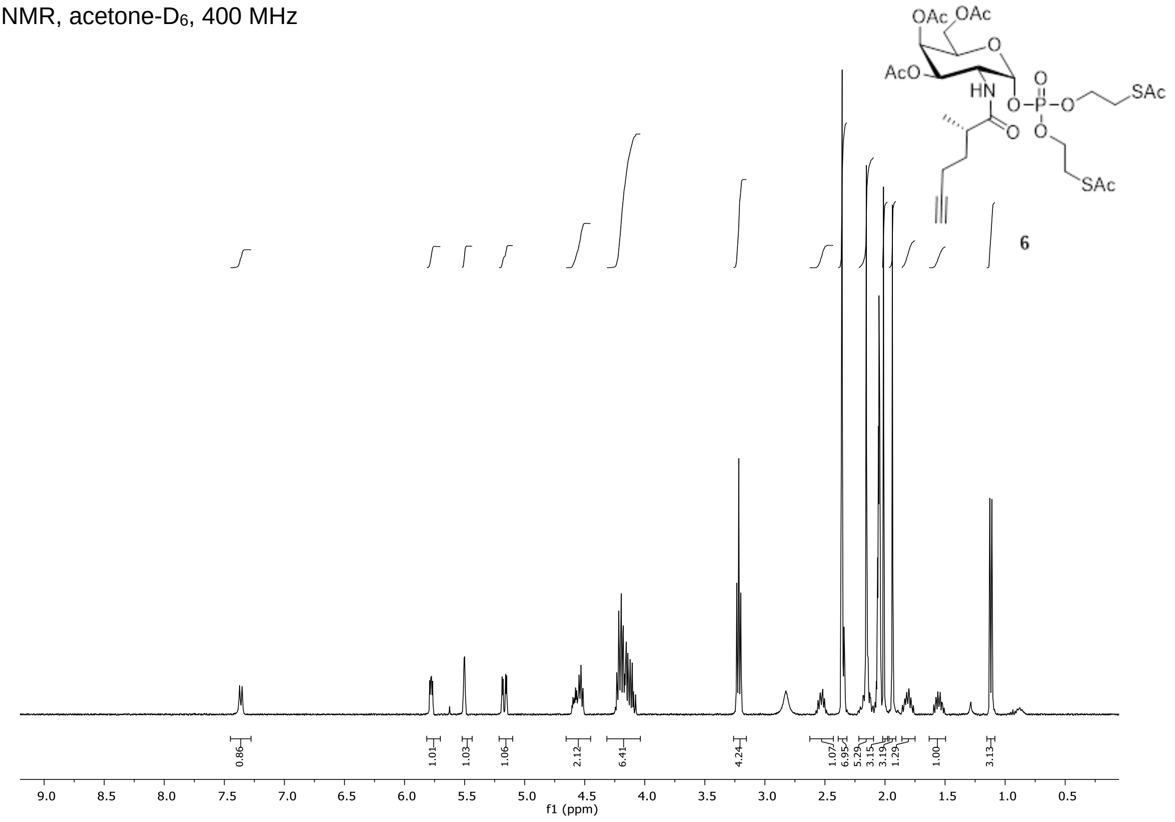

^{13}C NMR, acetone-D_6, 100 MHz

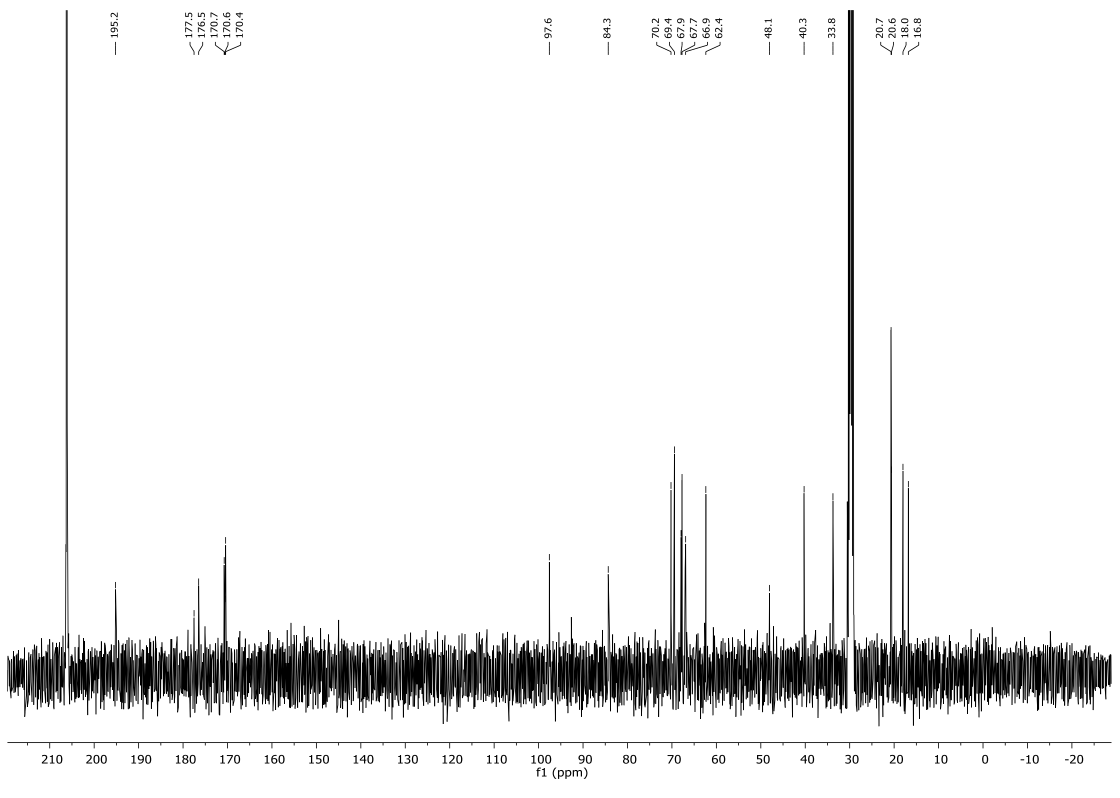

^{1}H NMR, acetone-D$_6$, 400 MHz

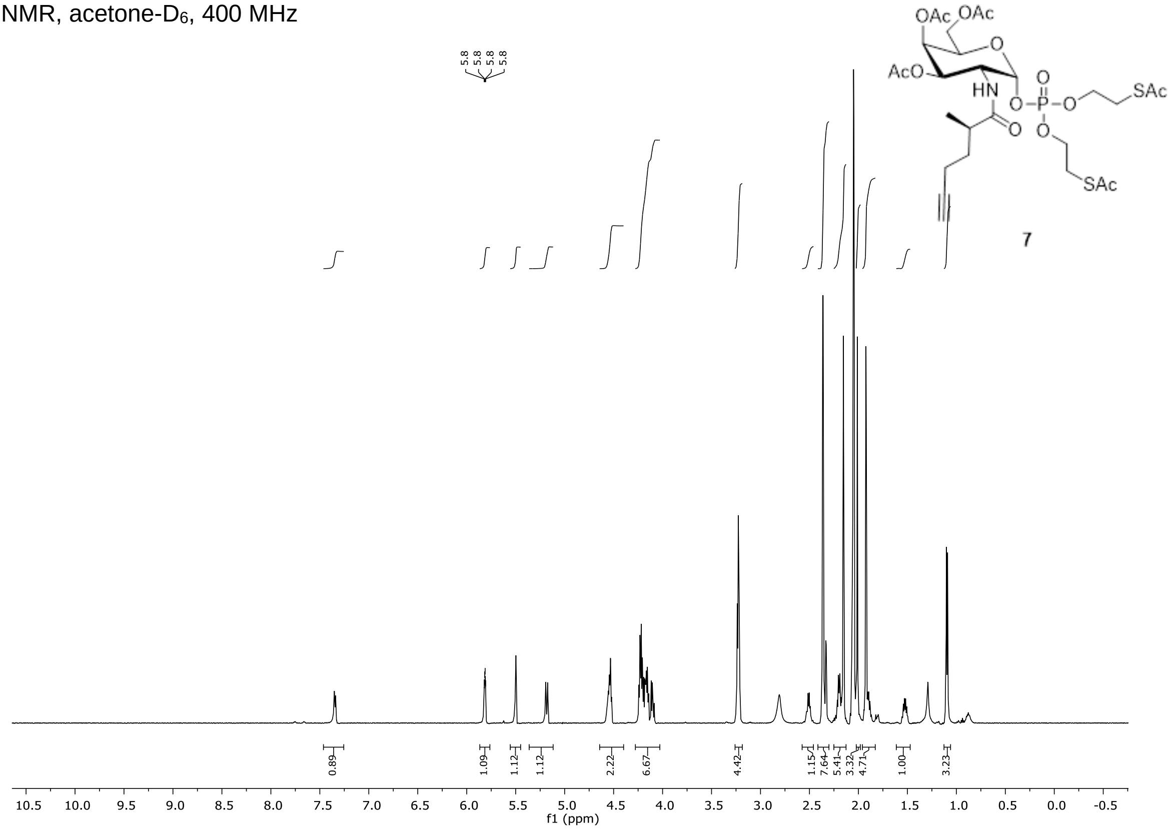

7

^{13}C NMR, acetone-D$_6$, 100 MHz

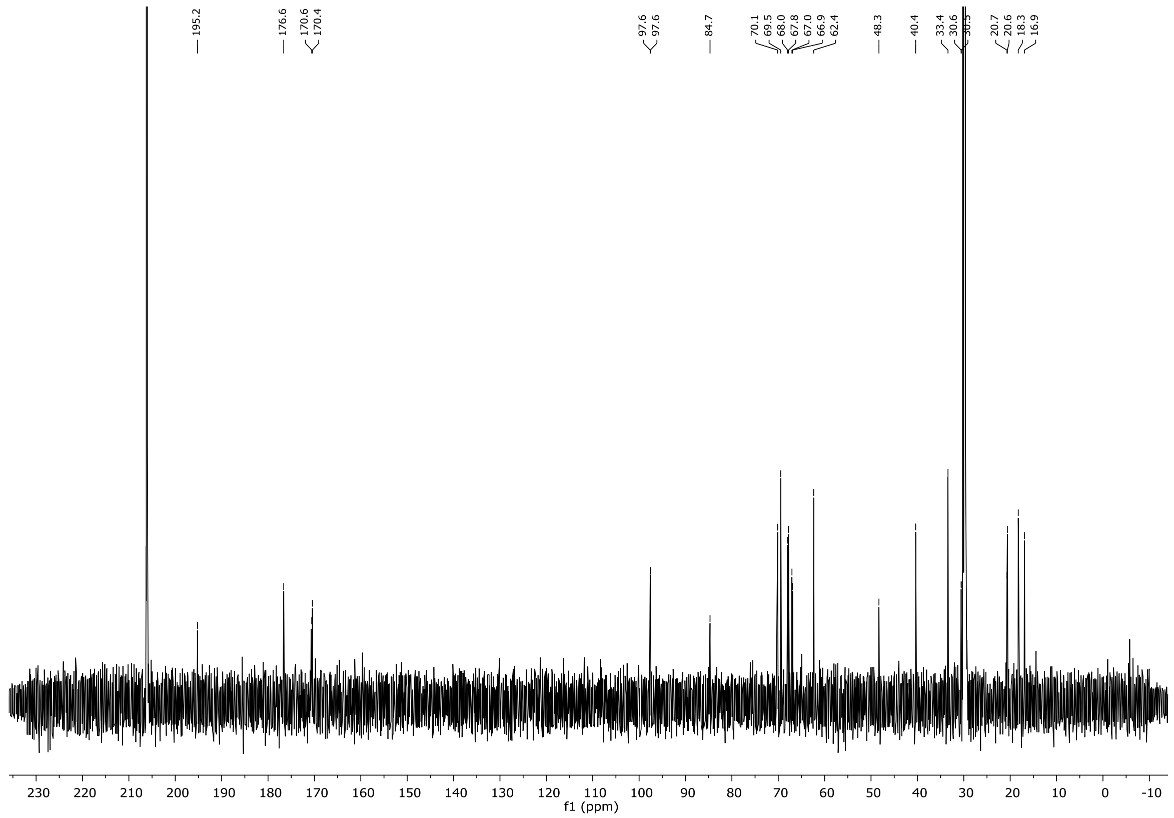

^{1}H NMR, $CDCl_3$, 400 MHz

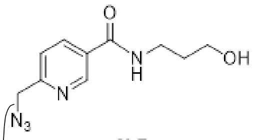

SI-7

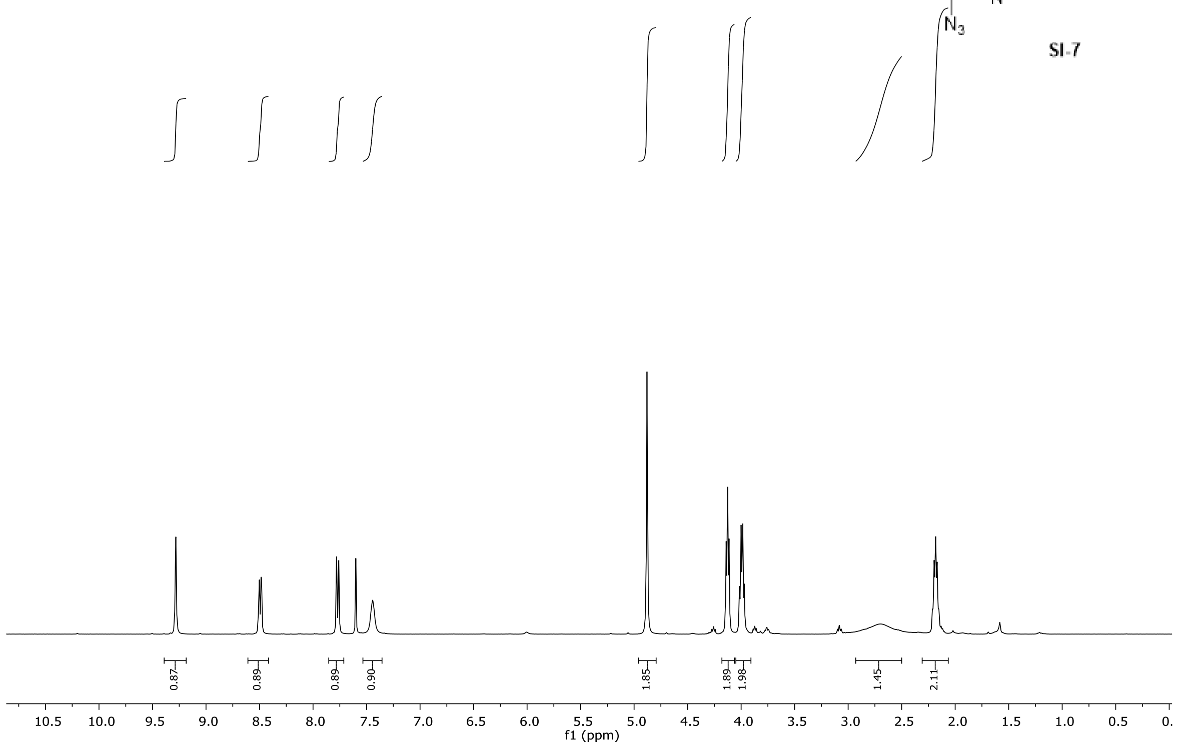

^{1}H NMR, $CDCl_3$, 100 MHz

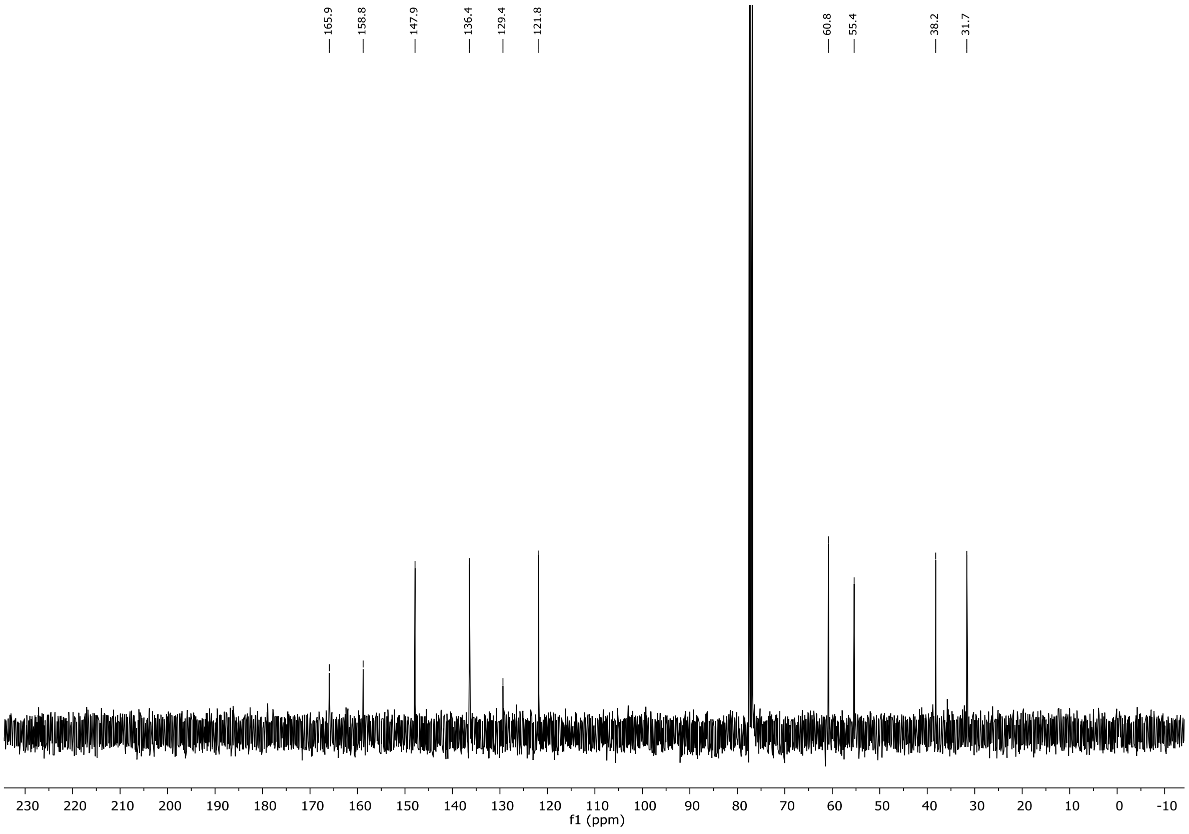

^{1}H NMR, $CDCl_3$, 400 MHz

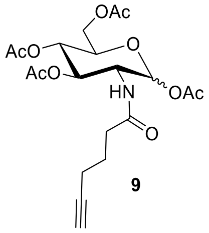

9

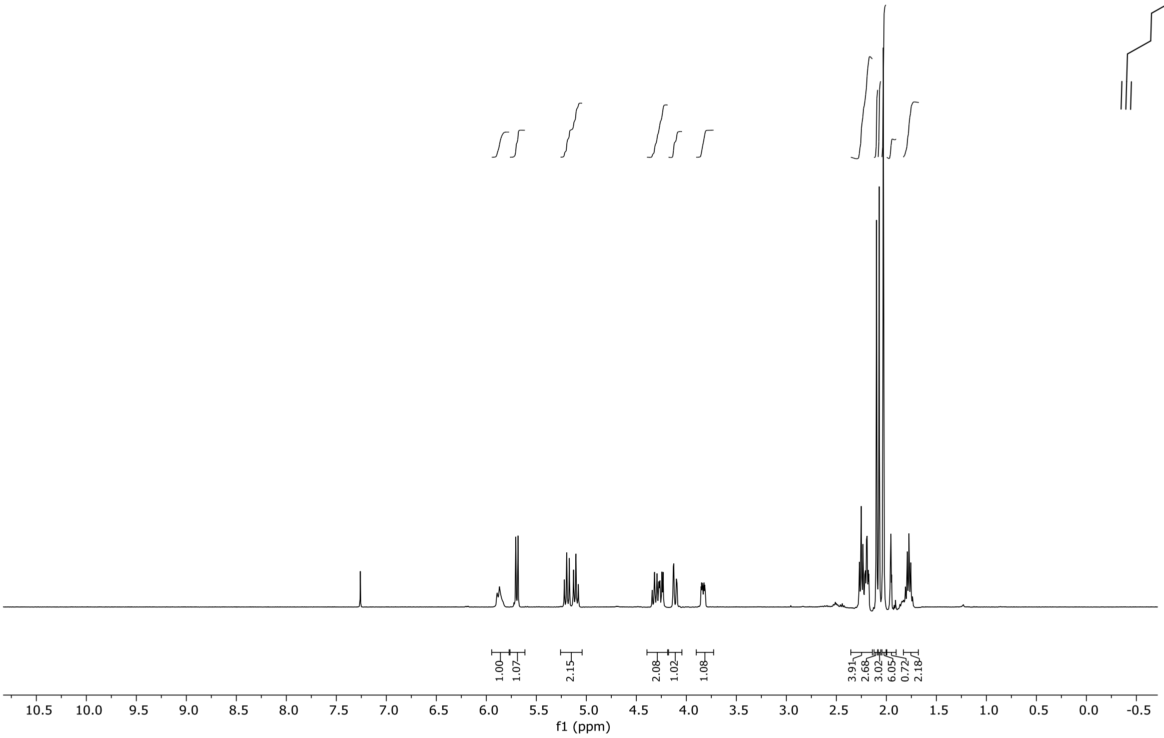

^{1}H NMR, $CDCl_3$, 600 MHz

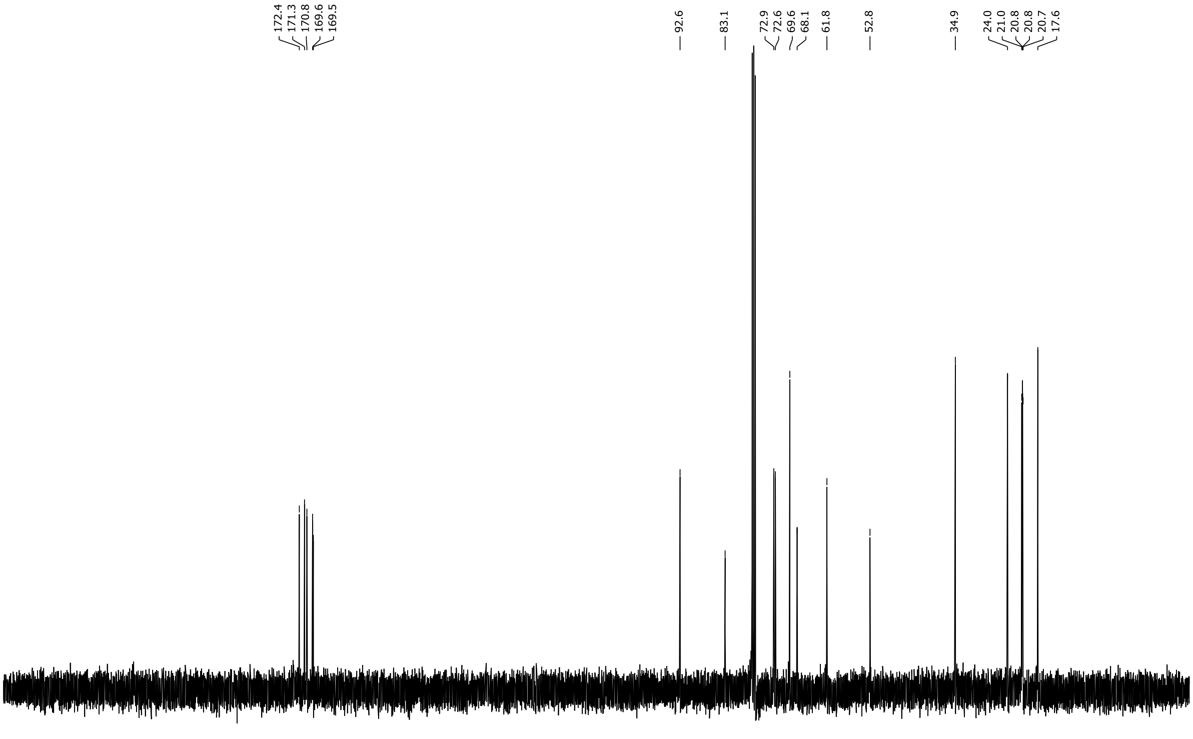

^{1}H NMR, $CDCl_3$, 600 MHz

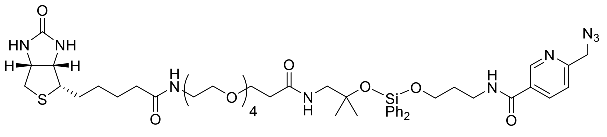

10

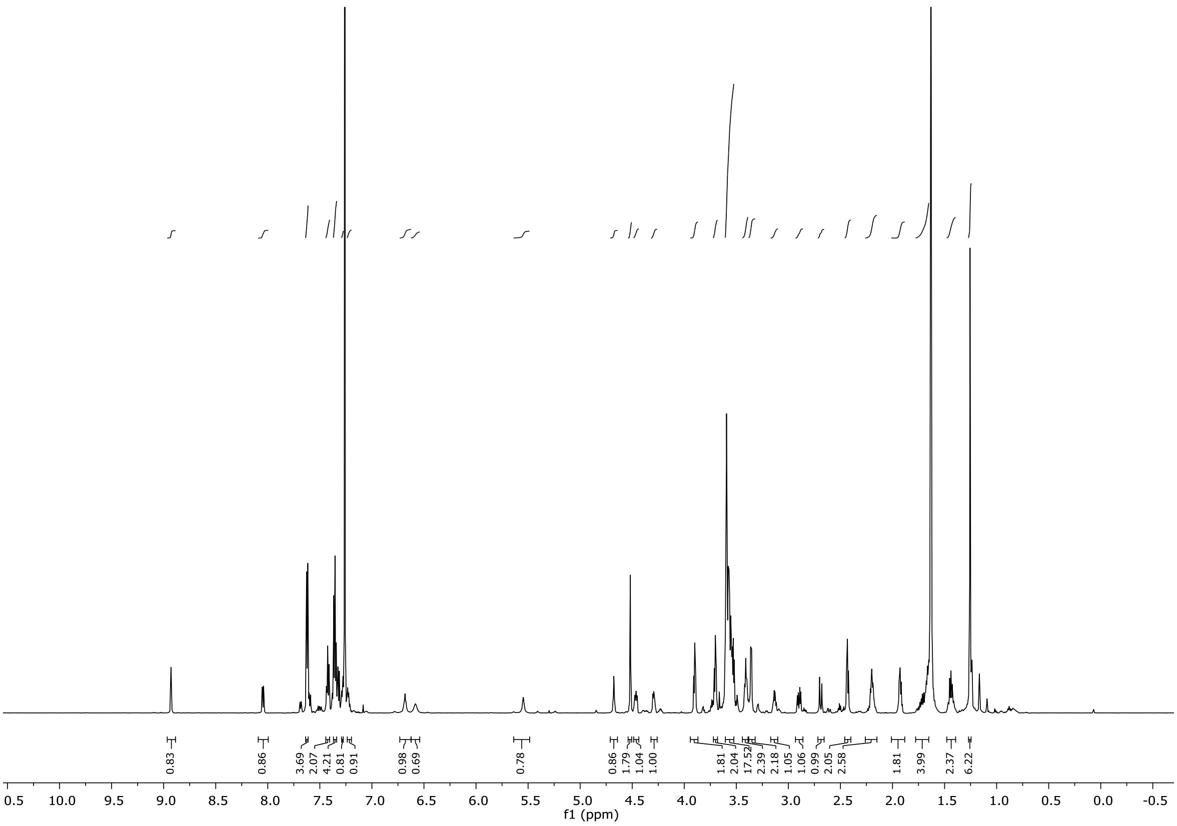

Supplement: Document S2. Article plus Supplemental Information [file mmc4.pdf]
